# Design and Synthesis of Eugenol Derivatives Bearing a 1,2,3-Triazole Moiety for Papaya Protection against *Colletotrichum gloeosporioides*

**DOI:** 10.1021/acs.jafc.4c00440

**Published:** 2024-05-21

**Authors:** Ângela Maria Almeida lima, Luíza Carvalheira Moreira, Poliana Rodrigues Gazolla, Mariana Belizario Oliveira, Róbson Ricardo Teixeira, Vagner Tebaldi Queiroz, Matheus Ricardo Rocha, Willian Bucker Moraes, Nayara Araújo dos Santos, Wanderson Romão, Valdemar Lacerda, Pedro Alves Bezerra Morais, Osmair Vital de Oliveira, Waldir Cintra de Jesus Júnior, Luiz C. A. Barbosa, Cláudia Jorge Nascimento, Jochen Junker, Adilson Vidal Costa

**Affiliations:** † Departamento de Química e Física, Universidade Federal do Espírito Santo, Alto Universitário, s/n, Guararema, Alegre 29500-000, Espírito Santo, Brazil; ‡ Departamento de Química, Universidade Federal de Viçosa, Av. P.H. Rolfs, s/n, Viçosa 36570-900, Minas Gerais, Brazil; § Departamento de Agronomia, Universidade Federal do Espírito Santo, Alto Universitário, s/n, Guararema, Alegre 29500-000, Espírito Santo, Brazil; ∥ Laboratório de Petroleômica e Forense, Departamento de Química, Universidade Federal do Espírito Santo, Av. Fernando Ferrari 514, Vitória 29075-910, Espírito Santo, Brazil; ⊥ Instituto Federal de São Paulo, Campus Catanduva, Catanduva 15808-305, São Paulo, Brazil; # Universidade Federal de São Carlos, Campus Lagoa do Sino, Buri 18290-000, São Paulo, Brazil; ¶ Departamento de Química, Universidade Federal de Minas Gerais, Av. Pres. Antônio Carlos 6627, Belo Horizonte 31270-901, Minas Gerais, Brazil; ∇ Departamento de Ciências Naturais, Instituto de Biociências, Universidade Federal do Estado do Rio de Janeiro (UNIRIO), Av. Pauster, Rio de Janeiro 22290-240, Rio de Janeiro, Brazil; ○ Centro de Desenvolvimento Tecnológico em Saúde, Fundação Oswaldo Cruz, Av. Brasil, 4365, Rio de Janeiro 21040-900, Rio de Janeiro, Brazil

**Keywords:** Colletotrichum gloeosporioides, fungicide activity, 1,2,3-triazole, papaya, molecular docking, eugenol

## Abstract

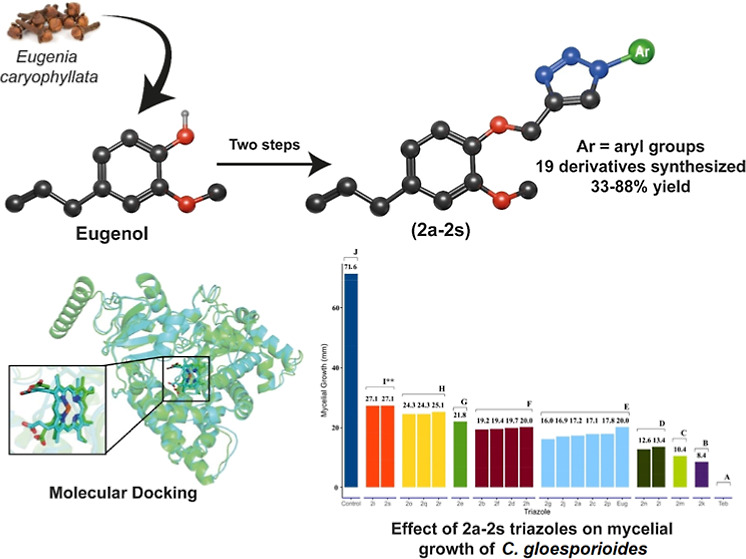

A series of 19 novel eugenol derivatives containing a 1,2,3-triazole moiety was synthesized via a two-step process, with the key step being a copper(I)-catalyzed azide–alkyne cycloaddition reaction. The compounds were assessed for their antifungal activities against *Colletotrichum gloeosporioides*, the causative agent of papaya anthracnose. Triazoles **2k**, **2m**, **2l**, and **2n**, at 100 ppm, were the most effective, reducing mycelial growth by 88.3, 85.5, 82.4, and 81.4%, respectively. Molecular docking calculations allowed us to elucidate the binding mode of these derivatives in the catalytic pocket of *C. gloeosporioides* CYP51. The best-docked compounds bind closely to the heme cofactor and within the channel access of the lanosterol (**LAN**) substrate, with crucial interactions involving residues Tyr102, Ile355, Met485, and Phe486. From such studies, the antifungal activity is likely attributed to the prevention of substrate **LAN** entry by the 1,2,3-triazole derivatives. The triazoles derived from natural eugenol represent a novel lead in the search for environmentally safe agents for controlling *C. gloeosporioides*.

## Introduction

The pursuit of healthier and more nutritious foods, such as fruits, has witnessed significant growth over the years.^1^ Papaya (*Carica papaya* L.), a staple crop in tropical and subtropical countries, holds substantial economic importance and is prominently cultivated and traded in Brazil, particularly in the regions of Espírito Santo, Bahia, and Ceará States.^2,3^ In acknowledging its nutritional and medicinal value, papaya is widely consumed, boasting high levels of vitamins (A, B1, B2, C, and E), minerals (potassium, calcium, phosphorus, and iron), and dietary fibers that contribute to intestinal regulation.^4,5^

Nevertheless, like other fruit crops, papaya is susceptible to fungal infections, resulting in significant production and economic detriment to farmers. *Colletotrichum gloeosporioides*, the causative agent of anthracnose in papaya, stands out as a primary postharvest disease, compromising the nutritional quality and productivity of the fruit, rendering it unfit for consumption and marketability.^5,6^ Small necrotic and circular lesions are among the primary symptoms observed in anthracnose-infected fruit tissues.^7^

In recent years, despite the development of numerous agricultural techniques to control phytopathogenic fungi, including biological control, utilization of botanical fungicides (such as plant extracts and essential oils), agronomy technology, and the application of ultraviolet light,^8,9^ the use of chemical agents known as fungicides remains a predominant choice.^10^ The preference for these chemical agents can be justified by their low cost, ease of application, and efficiency compared with other techniques. However, the indiscriminate application of fungicides poses health and environmental risks due to their residual chemical presence in fruits, soil, and water. Furthermore, the emergence of pathogen resistance necessitates the exploration of novel, more efficient, and environmentally friendly compounds.^11,12^

In light of these challenges, our research group has focused on developing efficient fungicides using molecular hybridization, a widely explored strategy for the pursuit of bioactive compounds.^13,14^ This technique involves combining two bioactive molecules, such as 1,2,3-triazole and eugenol, to enhance the fungicidal activity of the resulting hybrid compounds.

Compounds containing 1,2,3-triazole moieties have attracted attention for their diverse biological activities including antibacterial,^15^ antiviral,^16^ leishmanicidal,^17^ antimalarial,^18^ anticancer,^19^ anticholinesterase,^20^ insecticide,^21^ and antifungal.^22^ It has been shown that the inclusion of a 1,2,3-triazole ring in the structure of different compounds enhances their bioactivities.^23^ Our research group has published compelling investigations on the fungicidal action of 1,2,3-triazole compounds against *C. gloeosporioides*, demonstrating the influential role of such group in their biological activities.^24−26^

Eugenol (C_10_H_12_O_2_, 4-allyl-2-methoxyphenol), a natural metabolite found abundantly in plants of *Eugenia caryophyllata* (also known as clove) species, presents various biological activities and has attracted considerable attention of researchers.^27^ Its derivatives exhibit leishmanicidal,^17^ insecticidal,^28^ anti-inflammatory,^29^ antibacterial,^30^ antioxidant^31^ properties. Notably, extensive chemical modification of eugenol has resulted in substances with antifungal activity.^32−35^

In continuation of our research to develop new fungicides for controlling *C. gloeosporioides*, this work details the synthesis and antifungal activities of 19 1,2,3-triazoles derived from eugenol. Additionally, this presents an in silico study involving molecular docking, offering insights into the mode of action of these compounds.

## Materials and Methods

### Chemicals and Instruments

The solvents were purchased from Êxodo Cientfica (Sumaré, São Paulo City, São Paulo State, Brazil) and Qumica Moderna (Barueri, São Paulo State, Brazil). Reagents and acetonitrile were procured from Sigma-Aldrich (St. Louis, MO, USA). Reaction progress was monitored by thin-layer chromatography (TLC) on silica-gel plates, that were visualized under ultraviolet light (λ = 254 nm), and further revealed with a potassium permanganate solution. Compounds were purified through silica-gel (70–230 mesh, Sigma-Aldrich) column chromatography, eluting with mixtures of hexane and ethyl acetate. Melting points were determined using an MQAPF-302 apparatus (Micro Qumica, Cotia, São Paulo, Brazil) and were not corrected. Infrared (IR) spectra were acquired using the attenuated total reflectance technique on a Varian 660-IR instrument equipped with a GladiATr accessory (Varian, Palo Alto, CA, USA) in the region of 4000–500 cm^–1^. Nuclear magnetic resonance, encompassing hydrogen (^1^H NMR) at 300, 400, and 600 MHz and carbon (^13^C NMR) at 75, 100, and 150 MHz were recorded, respectively, on Varian Mercury 300 MHz (Varian, Palo Alto, CA, USA), Bruker AVANCE III 400 MHz (Bruker, Billerica, MA, USA), and Premium Compact 600 MHz (Bruker, Billerica, MA, USA) spectrometers. Chloroform (CDCl_3_) and dimethyl sulfoxide (DMSO-*d*_6_) served as deuterated solvents. Coupling constants (*J*) were expressed in Hertz (Hz) and chemical shift (δ) in ppm. Signal multiplicities were denoted as multiplet (m), singlet (s), broad singlet (brs), doublet (d), doublet of doublets (dd), doublet of doublets of triplets (ddt), triplet (t), triplet of doublets (td), and quartet (q). Chromatographic analysis was conducted on a Vanquish Flex ultraefficiency liquid chromatograph coupled to the LTQ-XL mass spectrometer (both from Thermo Scientific, Bremen, Germany). Separation occurred on a Luna Omega C18 column, 1.6 μm, 150 × 2.1 mm (Phenomenex, São Paulo City, São Paulo State, Brazil), with 2 μL samples injected at a flow rate of 350 μL min^–1^. The sample was eluted with a mixture of water and methanol, both containing 0.1% formic acid, with a gradient ranging from 5.0 to 95.0% water over 7 min at 60 °C. Mass spectra were recorded in the *m*/*z* = 100–1500 range, employing positive ionization mode. Heated ESI source parameters included a heater temperature of 350 °C, sheath gas flow rate of 30 arb, auxiliary gas flow rate of 10 arb, spray voltage of 4.0 kV, and capillary voltage of 44.0 V. Calibration of the LTQ-XL equipment was performed using a CalMix LTQ solution, positive mode, within the *m*/*z* = 100–2000 mass range, with ion accumulation time of 0.005 s and capillary voltage of 4.0 kV. Spectra were processed using the Xcalibur program, version 2.2 (Thermo Scientific, Bremen, Germany). MS/MS experiments utilized 20.0% normalized energy.

### Extraction and Purification of Eugenol

Eugenol was extracted and purified following the methodology described in the literature.^36^

### Synthetic Procedures

#### Synthesis of 4-Allyl-2-methoxy-1-(prop-2-yn-1-yloxy)benzene (**1**)

A 50 mL round-bottom flask was charged with eugenol (1.20 g, 7.32 mmol), sodium hydroxide (0.313 g, 7.38 mmol), and 20.0 mL of methanol. The resulting mixture was stirred at 40 °C for 30 min. Subsequently, methanol was removed under reduced pressure. Under a nitrogen atmosphere, 25.0 mL of acetonitrile and propargyl bromide (800 μL, 8.79 mmol) were slowly added to the same flask. The reaction mixture was then stirred magnetically at room temperature for 24 h. After completion of the reaction, as indicated by TLC analysis, the solvent was removed under reduced pressure using a rotary evaporator. To the residue obtained, an aqueous solution of sodium hydroxide (25.0 mL, 0.1 mol L^–1^) was added, and the resulting mixture was transferred to a decanting funnel. The aqueous phase was extracted with dichloromethane (3 × 20.0 mL) and the combined organic extracts was washed with NaCl aqueous solution (20.0 mL), dried over anhydrous sodium sulfate, filtered, and concentrated under reduced pressure. The obtained residue was fractioned by silica gel column chromatography to afford compound **1**. Structural characterization data of compound **1** along with spectra used in its characterization can be found in the Supporting Information (Figures S1–S3).

#### General Procedure for the Synthesis of 1,2,3-Triazole Compounds (**2a**–**2s**)

A 25 mL round-bottom flask was charged with the required azide (1.0 equiv), alkyne **1** (1.0 equiv), sodium ascorbate (0.40 equiv), 2.0 mL of distilled water, and 2.0 mL of ethanol. To this flask was added CuSO_4_·5H_2_O (0.20 equiv), and the resulting mixture was vigorously stirred for 24–48 h at room temperature. Upon completion, as revealed by TLC analysis, the resulting mixture was extracted with dichloromethane (3 × 20.0 mL). The organic extract was washed with saturated Na_2_CO_3_ solution (10 mL) and subsequently dried over anhydrous sodium sulfate, filtered, and concentrated under reduced pressure to afford a residue. This crude residue was purified by silica gel column chromatography to afford eugenol derivatives **2a**–**2s**. Structural characterization data of compound **2a**–**2s** along with spectra used in the characterization of them can be found in the Supporting Information (Figures S4–S98).

### Biological Assays

#### Fungicidal Effect of Triazoles **2a**–**2s** on *C. gloeosporioides*

To assess the fungicidal efficacy of triazoles in inhibiting the mycelial growth of *C. gloeosporioides*, an initial experiment was carried out using a completely randomized design with five replicates. Each treatment involved the application of triazole at a concentration of 100 ppm. Commercial tebuconazole (100 ppm) served as the positive control, while a 3.4% (v v^–1^) solution of dimethyl sulfoxide (DMSO) was employed as the negative control. In the experiment, triazoles were incorporated into a potato-dextrose-agar (PDA) culture medium that was still in a molten state and contained 3.5% DMSO (v v^–1^). The resulting culture medium for each treatment was transferred to Petri dishes. Upon solidification, a PDA disk containing *Colletotrichum gloeosporiodes*, cultured at 25 °C for 10 days, was placed in the center of each plate. The Petri dishes of each treatment were maintained at 25 ± 1 °C with a 12 h photoperiod. The evaluation of the effect of each triazole on mycelial growth occurred after *C. gloeosporiodes* covered the entire diameter of the Petri dishes of the positive control. The experiment was repeated twice over time.

The four most efficient triazoles, which caused a mycelial growth reduction ≥80% at 100 ppm, were selected for a second experiment. The mycelial growth of *C. gloeosporiodes* was assessed according to the methodology described above but at concentrations of 3.125, 6.25, 12.5, 25, 50, and 100 ppm. Tebuconazole and DMSO at the same concentrations served as the positive and negative control treatments, respectively. The second experiment was also carried out in a completely randomized design with five replicates and repeated twice over time.

### Data Analysis

The data obtained from the fungicidal activity tests of the triazoles were analyzed using R Software version 4.3.1 (2023). In the first experiment, which focused on the relationship between the mycelial growth of *C. gloeosporiodes* and the interaction of triazoles **2a**–**2s** at 100 ppm (μg mL^–1^), the data were subjected to analysis of variance. Subsequently, the Skott–Knott mean test at 5% probability was applied to delineate the most and least efficient groups, utilizing the “ExpDes.pt” package.^37^ For the second experiment involving the most efficient triazoles, the data were subjected to regression analysis. The EC_50_ (concentration of the compound to inhibit 50% of the mycelial growth) was estimated for each triazole using the “ec50estimator” package,^38^ along with the determination of the coefficients of the regression equation. Finally, the results were visually represented through graphics created with the assistance of the “tideverse” package.^39^ To summarize, the efficiency of the triazoles was characterized based on the scale proposed by Edgington et al. (1971).^40^

### Molecular Docking

The 3D structure of the sterol 14α-demethylase (CYP51A) from *C. gloeosporioides* was retrieved from the UniProtKB database (https://www.uniprot.org/uniprotkb/) with accession code T0KUT7. The heme cofactor was absent in this enzyme, and it was acquired by superimposing it with the crystal structure of *Aspergillus fumigatus* sterol 14α-demethylase (PDB code: 4UYM).^41^ Subsequently, this enzyme–heme complex, hereafter termed CgCYP51A, was employed in the docking calculations. Additionally, the heme was docked into the catalytic activity pocket of the apoenzyme to validate our docking procedure. The AutoDockTools (ADT) software^42^ was utilized to convert the enzyme into PDBQT format, where nonpolar hydrogen was merged onto carbon atoms, and Gasteiger charges were assigned to each atom. The structures of the triazole derivatives were drawn and preoptimized using the Avogadro software [Avogadro: an open-source molecular builder and visualization tool. Version 1.93.0. http://avogadro.cc/]. For the refinement of their molecular conformations, they were reoptimized using the semiempirical Hamiltonian PM7 method^43^ with the MOPAC2016 package.^44^ The OBABEL^45^ program was employed to convert the optimized structures into PDBQT format. The compounds **2a**–**2s** were docked onto the activity site and substrate access channel of CgCYP51A. The docking was performed within a grid box of dimensions 34 × 34 × 34 Å, with a grid spacing of 1 Å, centered at coordinates 5.088 × −6.378 × 0.51.

All molecular conformations of the ligands were treated as flexible, while CgCYP51A was considered a rigid body. A total of 20 binding models with an exhaustiveness of eight were considered for each ligand. It is noteworthy that a similar approach was recently used by our research group to study the CYP51 from *Saccharomyces cerevisiae*.^46^ All molecular docking calculations were performed using the AutoDock Vina package.^47^ The results were analyzed using the PyMOL software version 2.0 [The PyMOL Molecular Graphics System, Version 2.0 Schrödinger, LLC.] and Discovery Studio (DS) Visualizer 21.1.0.20298 (https://discover.3ds.com/discovery-studio-visualizer-download).

## Results and Discussion

### Chemistry

**Scheme 1:**
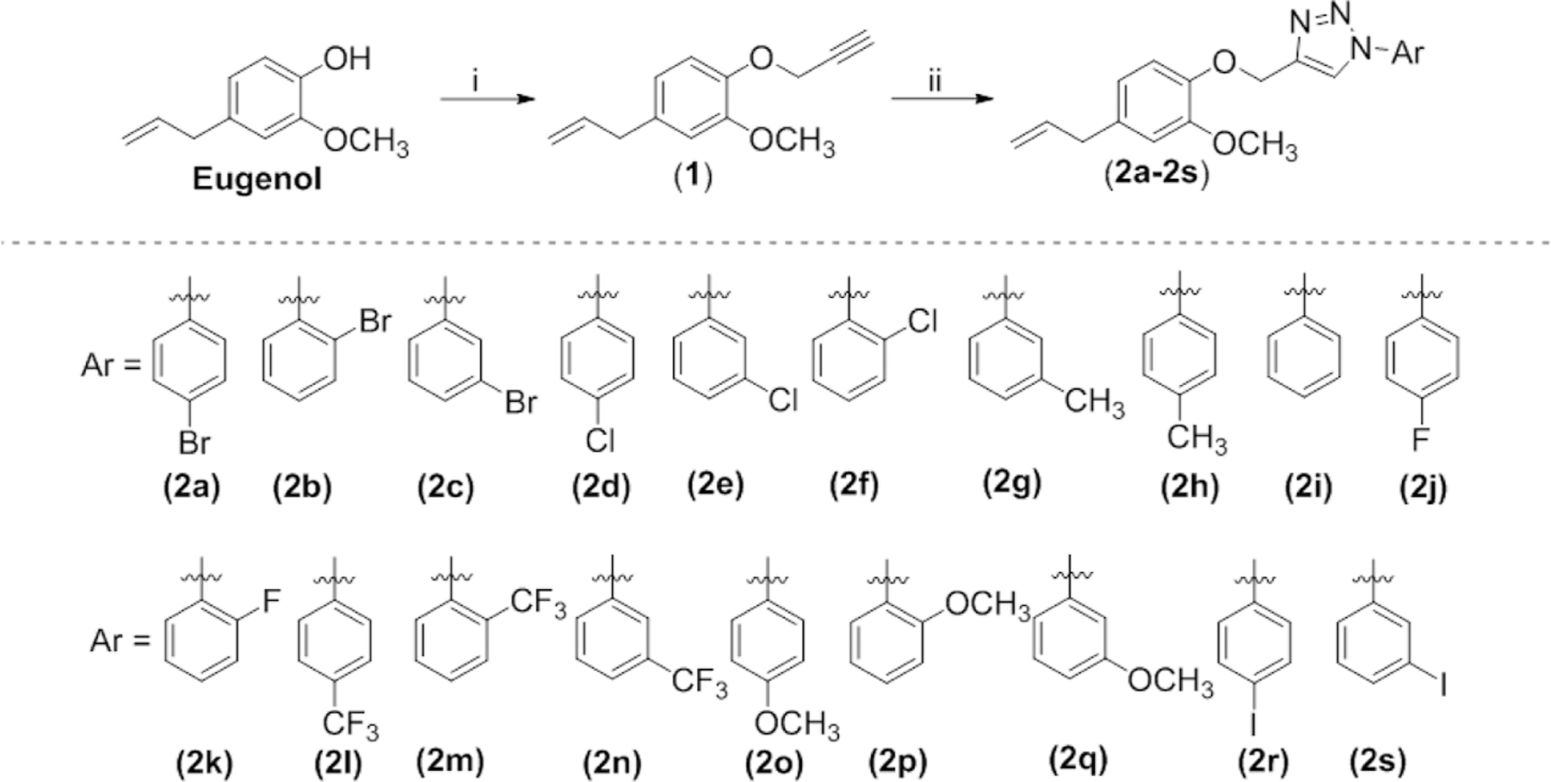
Preparation of 1,2,3-Triazole Compounds Derived from **Eugenol 2a–2s** Reagents and conditions: (i) NaOH, MeOH, HC≡CCH_2_Br, CH_3_CN, 40 °C → r.t., (81.0% yield); (ii) Ar–N_3_, Sodium Ascorbate, EtOH, H_2_O, CuSO_4_·5H_2_O, 50 °C, (33.0–88.0% yield).

The 1,2,3-triazole compounds, designated as **2a**–**2s** and derived from eugenol, were synthesized by following the reaction sequence outlined in Scheme 1. Initially, a bimolecular nucleophilic substitution reaction between eugenol and propargyl bromide, in the presence of sodium hydroxide, yielded terminal alkyne **1** in 81.0% yield after purification by silica gel column chromatography. Subsequently, to investigate the impact of diverse aromatic substituents linked to the triazole moiety on the bioactivity, 19 eugenol derivatives containing 1,2,3-triazole fragments **2a**–**2s** were synthesized. These new compounds, obtained with yields of 33.0–88.0%, were prepared from the 1,3-dipolar cycloaddition reaction catalyzed by Cu(I) between alkyne **1** and various commercially available aromatic azides. As previously mentioned, all the compounds were purified by silica gel column chromatography, and they were obtained with purity higher than 95% as determined by high-performance liquid chromatography chromatographic analysis (data not shown). Following synthesis, the compounds were characterized using spectroscopic techniques (IR, ^1^H NMR, and ^13^C NMR) and mass spectrometry. The IR spectra exhibited characteristic bands associated with the triazole structures, including stretching vibrations of =CH and C=C bonds in aromatic rings at 3094–3072 and 1590–1462 cm^–1^, respectively. The intense band around 1230 cm^–1^ was associated with the C–O–C stretching of ether moiety, and the stretching of the N=N bond of the triazole ring was observed between 1638–1634 cm^–1^, a distinctive feature of triazole-containing compounds. In the ^1^H NMR spectra, the singlets observed at 3.73 ppm (integrated for three hydrogens each) and 8.90 ppm (integrated for one hydrogen each) were attributed, respectively, to the methoxy group (−OCH_3_) and the hydrogen atom of the triazole ring. The doublet of doublet of triplet signals at 5.96 ppm (*J* = 16.8, 10.2, and 6.6 Hz), integrated for one hydrogen each, were assigned to vinyl hydrogens. In the ^13^C NMR spectra, the signals observed at around 39.5 ppm were attributed to the allylic carbon, while the carbons of the methoxy groups (–OCH_3_) appeared at 55.3 ppm. Triazole ring carbons were observed at 120.6 and 144.6 ppm. Additionally, signals at 124.2 ppm (q, *J*_C–F_ = 270.0 Hz, –CF_3_) and 162.1 ppm (d, *J*_C–F_ = 244.5 Hz) were detected in some compounds, corresponding to the carbon couplings with fluorine atoms. Mass spectra obtained by LC–MS/MS confirmed the molecular formulas expected for the triazoles, providing further validation of the synthesis process and compound identities.

Several commercial 1,2,4-triazoles feature an aromatic ring with various substituents, similar to those present in the compounds investigated in our study. This fact influenced our choice of alkynes for the synthesis of eugenol derivatives bearing 1,2,3-triazolic functionalities. By utilizing alkynes with structural motifs similar to those of commercially available 1,2,4-triazoles, we aimed to explore potential structural similarities and differences that could impact biological activity.

Furthermore, the incorporation of different substituents at various positions on the aromatic ring introduces modifications to the physical and chemical properties of the compounds. These alterations encompass parameters such as polarity, total surface area, and volume, among others. Such variations often lead to diverse biological responses. Thus, by systematically varying the substituents, we sought to gain insights into the structure–activity relationships of the compounds. This knowledge not only aids in understanding the underlying mechanisms of biological activity but also paves the way for the development of more effective compounds with tailored properties.

### Fungicide Activity of Triazoles **2a–2s** on *C. gloeosporioides*

**Figure 1:**
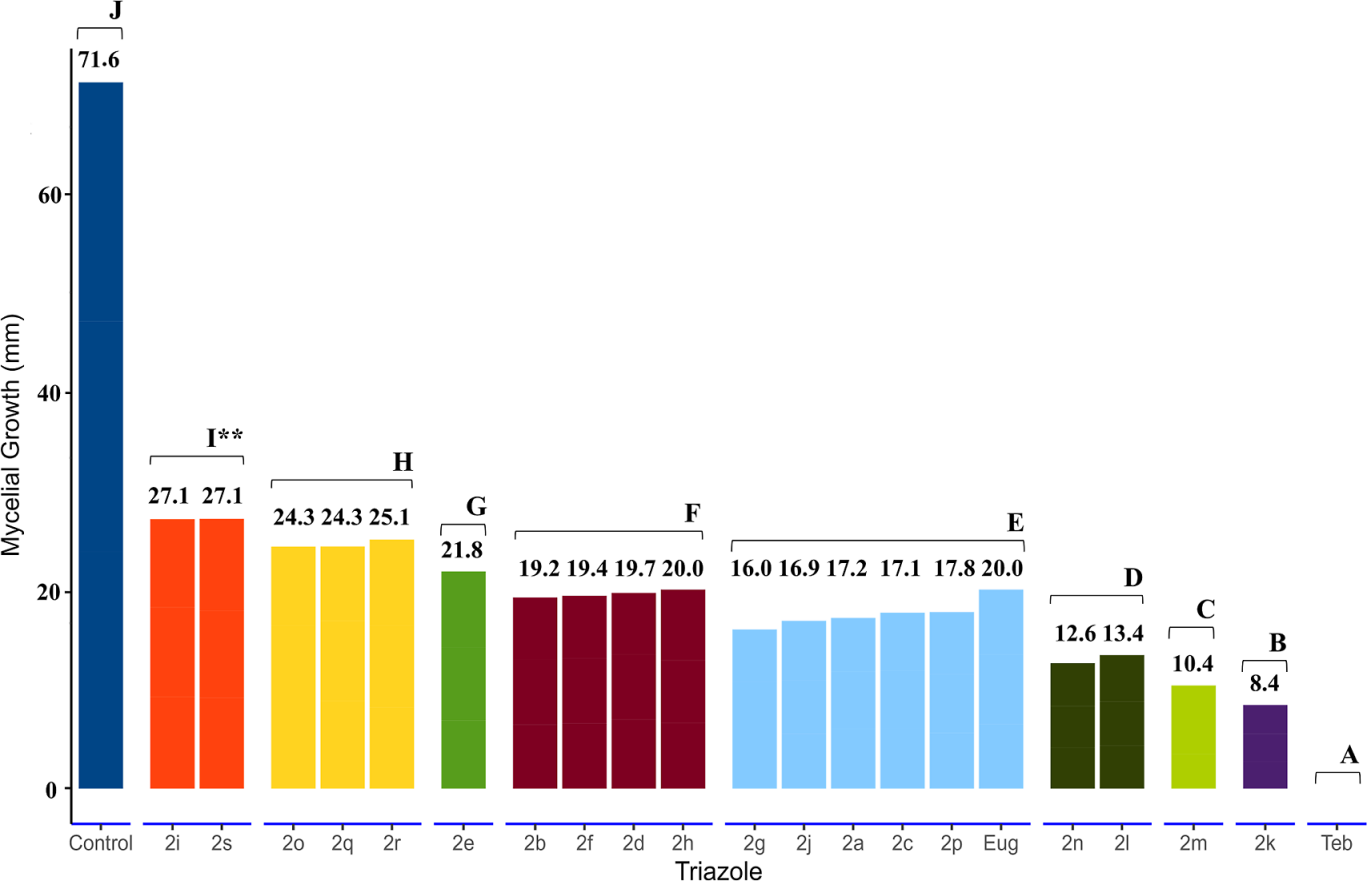
Effect of triazoles **2a**–**2s** and **eugenol** (**Eug**) at 100 ppm on mycelial growth of *C. gloeosporioides* (mm). The values above each bar represent the mean mycelial growth values (mm) for each triazole*. The letters above the bar and color variation represent the division of more and less efficient groups according to the Skott–Knott test at the 5% probability level**. **Teb**—**Tebuconazole** (100 ppm).

The fungicide potential of triazoles **2a**–**2s** was assessed on the mycelial growth of *C. gloeosporioides*, and all compounds demonstrated effectiveness in reducing pathogen growth at 100 ppm (Figure 1).

Considering the halogens (F, Cl, Br, and I) and the ortho, meta, and para positions that these substituents can occupy in the aromatic ring of a phenyl group, the data in Figure 1 reveal that the presence of a fluorine atom in the ortho position not only produced the most active derivative among the halogenated compounds (**2a**–**2f**, **2j**, **2k**, **2r**, and **2s**) but also resulted in the derivative with the highest fungicidal activity among all synthesized compounds. Another important aspect is that the presence of the strongly electron-withdrawing CF_3_ group in the ortho, meta, or para positions resulted in three derivatives that are among the most effective ones.

The presence of electron-donating groups (CH_3_ or OCH_3_) produced derivatives that showed better efficacy in inhibiting the mycelial growth of the *C. Gloesporioides* species as compared to **eugenol** (**Eug**). In this regard, compound **2p** presenting a methoxy group at the meta position showed the best inhibitory effect among the compounds with electron-donating groups.

**Figure 2:**
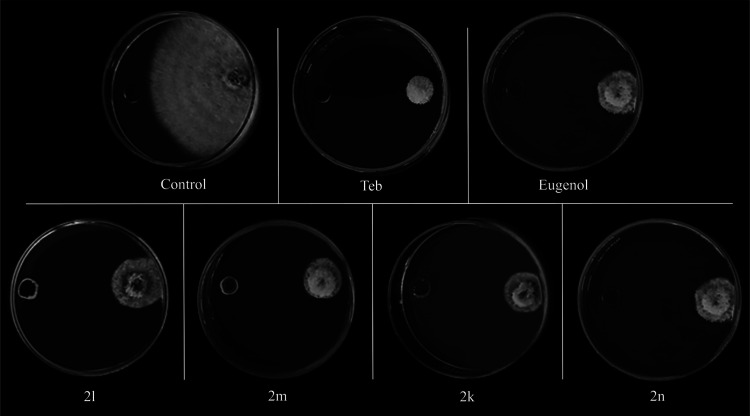
Effect of compounds **2k–2n** and **eugenol** (**Eug**) at 100 ppm on mycelial growth of *C. gloeosporioides*. The first two plates on the top represent the negative control (DMSO, 3.4% v/v) and positive control [**Tecobunazole** (**Teb**), 100 ppm)].

Notably, the most active triazoles **2k**, **2m**, **2l**, and **2n** (Figures 1 and 2) inhibited mycelial growth by, respectively, 88.3, 85.5, 82.4, and 81.4%.

Conversely, for those less efficient, **2s**, **2i**, **2r**, and **2q**, the observed variations in mycelial growth reduction were 61.7, 62.3, 65.5, and 66.2%, respectively. The positive control, featuring **Tebuconazole** (**Teb**), exhibited complete inhibition of pathogen growth at 100 ppm.

Based on the Skott–Knott test, eight groups of triazoles were distinguished according to the average change in mycelial growth of *C. gloeosporioides*, excluding the positive control (indicated by letter J in Figure 1). Triazoles that caused reductions exceeding 80% were deemed the most promising among the studied compounds and were subsequently employed to determine the effective doses for inhibiting 50 and 90% of the mycelial growth of *C. gloeosporioides*.

**Figure 3:**
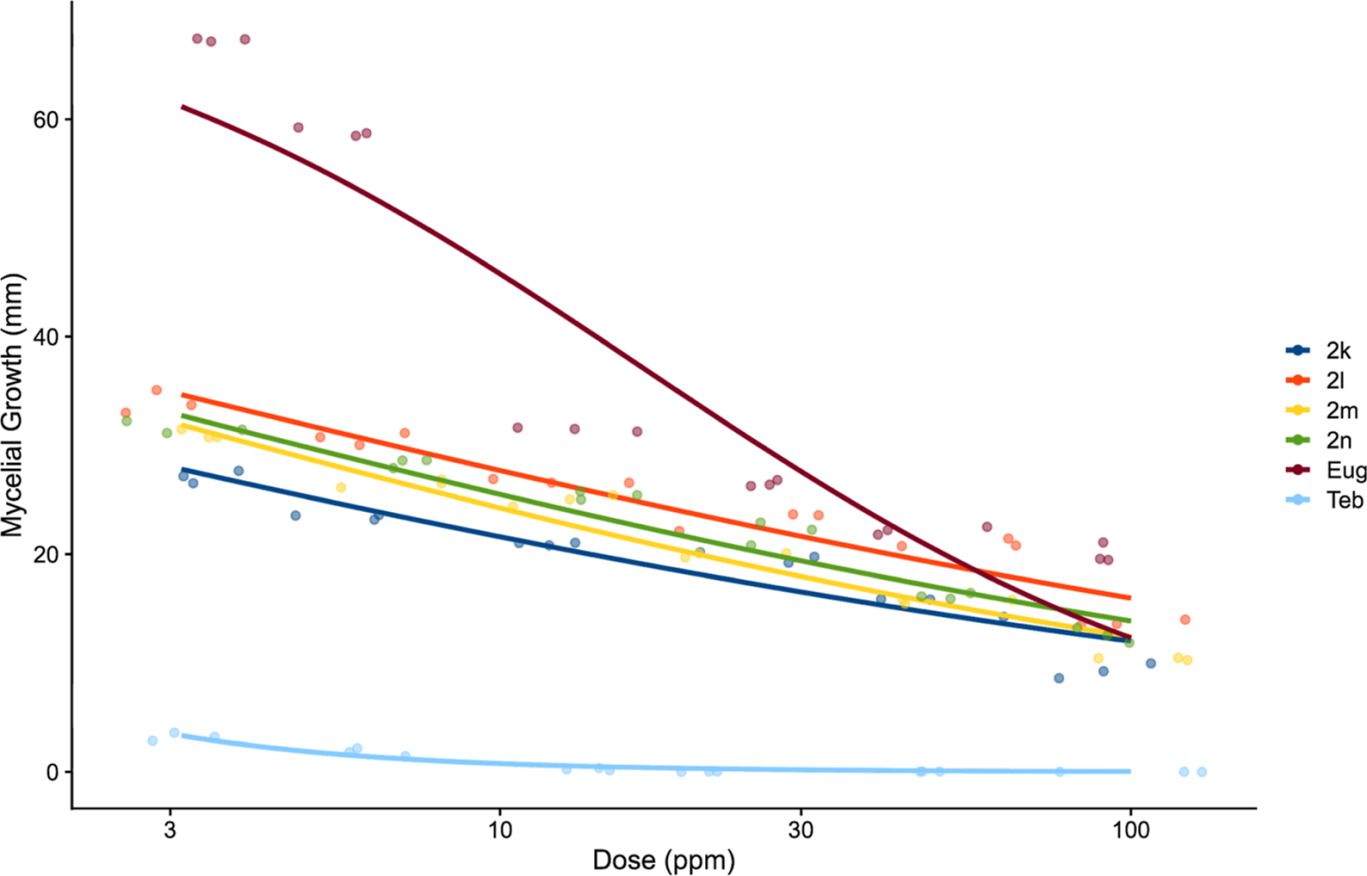
Effect of doses 3.125, 6.25, 12.5, 25, 50, and 100 ppm of triazoles **2k–2n, eugenol** (**Eug**) and **tebuconazole** (**Teb**) on mycelial growth of *C. gloeosporiodes* (mm).

The relationship between the dosage of triazoles (**2k**–**2n**) and Tebuconazole and the corresponding reduction in mycelial growth is depicted in Figure 3. This graphical representation indicates a dose-dependent trend, suggesting an inverse proportionality between the variables. As can be observed from this figure, the inhibition of mycelial growth decreases at a lower concentration of the triazoles. Based on these data, the EC_50_ was calculated and presented in Table 1. It is noteworthy that pathogen inhibition did not reach 100% at the doses investigated except for the positive control featuring Tebuconazole. The consistency in the regression behaviors observed for all four compounds further supports the similarity in their effectiveness.

**Table 1:** Effective Doses of Triazoles **2k–2n**, **Eugenol** (**Eug**) and **Tebuconazole** (**Teb**) to Inhibit 50 and 90% of Mycelial Growth of *C. gloeosporioides* and Regression Models^a^

compound	regression equation	aEC_50_ (ppm)	aEC_90_ (ppm)	effectiveness (E)
**2k**	*y* = 43.2 – 1.14*x* + 0.00082*x*^2**^	1.83	343.36	bME
**2l**	*y* = 47.4 – 1.08*x* + 0.00766*x*^2**^	12.13	682.20	cLE
**2m**	*y* = 45.9 – 1.2*x* + 0.00868*x*^2**^	4.81	292.35	ME
**2n**	*y* = 46.6 – 1.19*x* + 0.00087*x*^2**^	5.92	421.38	ME
**Eug**	*y* = 66.5 – 1.7*x* + 0.0126*x*^2**^	1.20	146.57	ME
**Teb**	*y* = 28.8 – 1.4*x* + 0.0114*x*^2**^	1.04	1.35	ME

a EC_50_ and EC90: Concentration of the compound to inhibit 50 and 90% of the mycelial growth of *C. gloeosporioides*, respectively.

b ME and.

c LE according to Edgington et al. (1971). Regression equation followed by ^**^ represents significance at a 1% probability.

According to the scale proposed by Edgington et al. (1971),^40^ the efficacy of a compound in reducing a variable associated with the studied pathogen is delineated by the estimated value of its EC_50_, with categorizations as follows: when EC_50_ < 1 ppm, it is classified as high efficiency (HE); if EC_50_ is in the range of 1–10 ppm, it is considered moderate efficiency (ME); for EC_50_ values falling within 10–50 ppm, it is designated as low efficiency (LE); and if EC_50_ exceeds 50 ppm, it is labeled as not efficient (NE). In our investigation, three of the four triazoles exhibited moderate efficiency in reducing the mycelial growth of *C. gloeosporioides*, with EC_50_ values ranging between 1.83 and 5.92 ppm, including eugenol (**Eug**, EC_50_ = 1.2 ppm). Furthermore, **2n** displayed low efficiency, while the molecule used in the control treatment, Tebuconazole, showed moderate efficiency (refer to Table 1). Despite both triazoles **2l** and **2n** being grouped by the Skott–Knott test, the EC_50_ value of triazole **2l** was twice that estimated for **2n**. This discrepancy led to the differentiation of the two compounds into distinct groups in terms of the efficiency categories proposed by Edgington et al. (1971).^40^

The variation in mycelial growth inhibitory activity of the compounds herein investigated may be attributed to their interaction with the physiological and structural characteristics of fungi. This fact has been demonstrated across different groups of phytopathogens, including *Sclerotinia scleotiorium*, *Botrytis cinerea*, and *C. gloeosporioides*.^24−26,48^ The relevance of triazoles in agricultural disease management extends to phytopathogen genera of global importance including Fusarium, *Aspergillus*, *Corynespora*, *Pseudocercospora*, and *Asperisporum*. Triazoles exhibit notable effectiveness against fungal diseases.^49−51^

The primary hypothesis explaining the effect of triazoles on fungi involves the inhibition of the enzyme lanosterol 14α-demethylase (CYP51). This inhibition results in the reduction of ergosterol, which is a crucial component of the plasma membrane responsible for maintaining fluidity, distributing integral proteins, and regulating their activity. The decrease in ergosterol leads to an accumulation of 14α-demethylated sterols, causing membrane disruption and eventual cell death.^52^ Results from this study suggest that compounds **2k**, **2m**, and **2n** exhibit significant potential for future applications, including their incorporation into new pesticides for anthracnose control in papaya (*C. papaya*) compared to the commercial fungicide Tebuconazole. The diversification of molecules with antifungal potential addresses challenges associated with the emergence of resistant phytopathogen populations, diminishing the efficacy of existing market compounds, and heightening the demand for innovative technologies. Consequently, characterizing the antifungal potential of 1,2,3-triazoles, such as those examined here, holds global importance.^53,54^ The new triazoles not only demonstrate high efficiency in reducing the mycelial growth of *C. gloeosporioides* but, due to their possible involvement in ergosterol biosynthesis, also exhibit potential for use in controlling other plant diseases caused by fungi in future studies. In the following section, we present the results of docking calculations illustrating the binding mode of triazole derivatives investigated herein with sterol 14α-demethylase (CgCYP51A) in *C. gloeosporioides*.

### Molecular Docking Analysis

**Figure 4:**
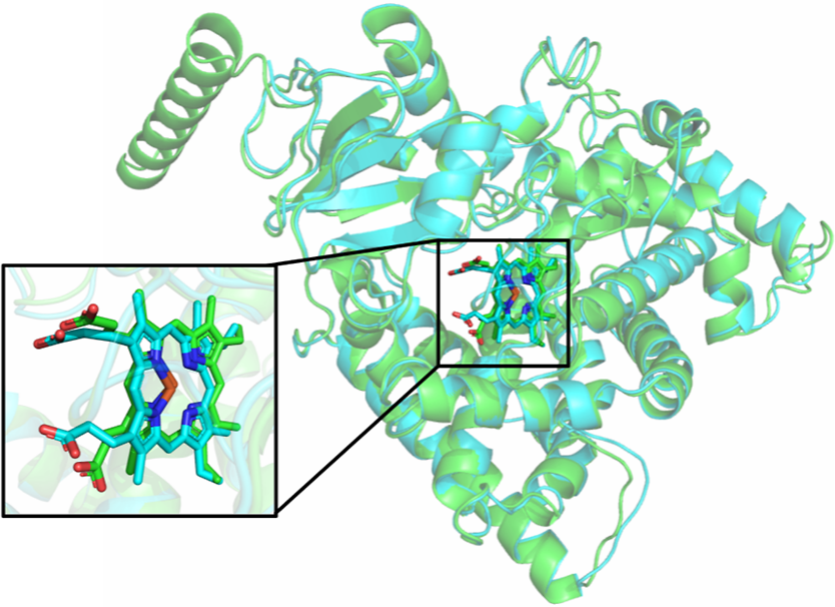
Overlapping between the CgCYP51A (green) and crystal structure (cyan) of the *A. fumigatus* CYP51 (PDB code: 4UYM). The zoom shows the alignment of the best-docked heme with its cocrystallized structure.

This in silico study employed docking calculations to elucidate molecular interactions and the binding mode between CgCYP51A and the triazole compounds herein investigated. As the enzyme structure complexed with the heme cofactor was not available in the UniProtKB open protein database, it was sourced from the *A. fumigatus* CYP51 (PDB code: 4UYM).^41^ Notably, this enzyme exhibits a high degree of similarity to *C. gloeosporioides* CYP51A, with a predicted amino acid sequence similarity of 57%, as determined by the online software tool CLUSTALW (https://www.genome.jp/tools-bin/clustalw). During the superposition of the structures of CgCYP51A and *A. fumigatus* CYP51, the root-mean-square deviation (rmsd) was 0.5 Å, indicating substantial structural similarity between the two enzymes. Consequently, the heme coordinates from *A. fumigatus* CYP51 were incorporated into the *C. gloeosporioides* CYP51A structure, resulting in the formation of the holo CgCYP51A enzyme. In an alternative approach, we also docked the heme in apo CYP51A and obtained a binding energy of −10.1 kcal mol^–1^. When comparing the structure of the best-docked conformation with the heme crystallographic structure no significant difference was noticed, with a rmsd value of 1.4 Å. Figure 4 shows the superposition of both enzymes and the best-docked heme cofactor complexed in the catalytic region.

As depicted in Figure 4, both enzymes exhibit a high degree of structural similarity, evident from the lower rmsd value. This similarity is further confirmed between the best-docked heme and its crystallographic structure. Taken together, these results validate the docking procedure and the software employed in this study.

**Figure 5:**
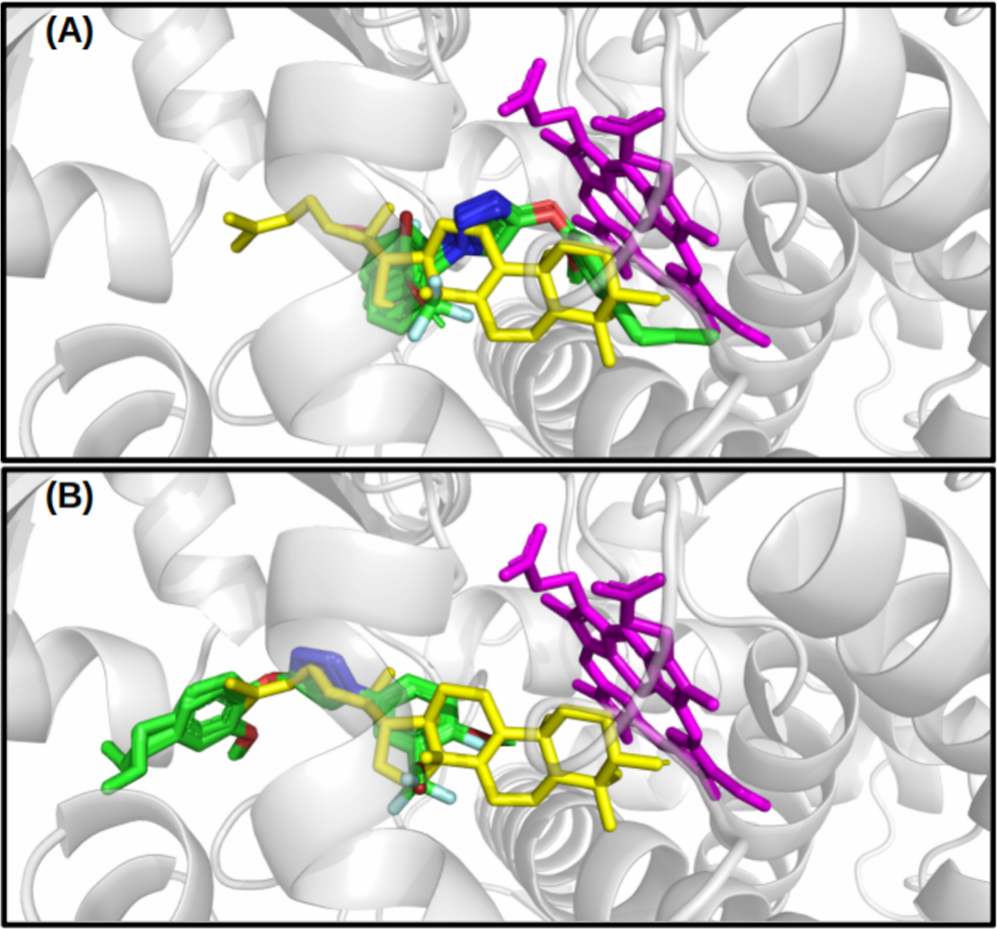
Best-docked compound on the catalytic pocket of the CgCYP51A. (A,B) are the compounds of G1 and G2 groups, respectively. In magenta, the heme cofactor, and in yellow, the lanosterol substrate.

The docking results led to the categorization of the compounds into two distinct groups: Group 1 (G1), comprising compounds **2b**, **2f**, **2i**, **2k**, **2m**, **2p**, and **2q**, situated close to the heme cofactor; and Group 2 (G2), encompassing compounds **2a**, **2c**, **2d**, **2e**, **2g**, **2h**, **2j**, **2l**, **2n**, **2o**, **2r**, and **2s**, which are docked within the channel access of the **LAN** substrate in CgCYP51. Figure 5 provides a visual representation of the CgCYP51A region where the best-docked compounds were obtained.

Figure 5 was constructed on the same scale for consistent comparison. To enhance clarity, the fungicide tebuconazole was not included in the illustration, although it is positioned near the heme cofactor. As depicted in Figure 5, the eugenol ring is directed toward the heme, and the entrance of the **LAN** channel accesses the G1 and G2 groups, respectively. Table S1 provides information on the docking energy and the residues involved in interactions with 1,2,3-triazole derivatives.

In addition, the key residues located at the CgCYP51A active site (Tyr102, Leu105, Thr106, Phe110, Val115, Tyr116, Phe209, Thr281, Gly285, Ser289, Ile355, Ser357, Ile358, Met485, and Phe486) are essential for interacting with substrate **LAN**. Therefore, as can be seen in Table S1 (Supporting Information), most of the residues in the CgCYP51A catalytic domain are interacting with the current derivatives.

The interacting residues, as presented in Table S1, were extracted using Discovery Studio (DS) Visualizer 21.1.0.20298 (https://discover.3ds.com/discovery-studio-visualizer-download). Negative values of the binding energy indicate favorable interactions between all triazole derivatives and CgCYP51A. It is noteworthy that the binding energies of all derivatives are lower than that of the fungicide **Tebuconazole** (**Teb**), elucidating their fungicidal action. While the binding energy of all derivatives is marginally higher than that of **LAN**, it suggests a competition between the triazole herein investigated and **LAN** for channel access and the active site of CgCYP51A, providing insights into their mechanism of action.

Experimental results revealed that triazoles **2k**, **2l**, **2m**, and **2n** were the most effective in reducing the mycelial growth of *C. gloesporiodes*. Except for compound **2k**, the molecular docking study corroborates this finding. The triazoles **2l**, **2m**, and **2n** exhibited the lowest binding energy (Table S1, Supporting Information). Conversely, compounds **2a**, **2i**, and **2q** displayed the highest docking energy, at −9.1 kcal/mol. Notably, **2q** and **2i** were the least efficient at reducing mycelial growth. In Table S1, compounds marked with an asterisk interact with the heme cofactor through their eugenol moiety, maintaining a distance of 3.3 Å between its oxygen (−OCH_3_) and the Fe atom in the heme factor.

**Figure 6:**
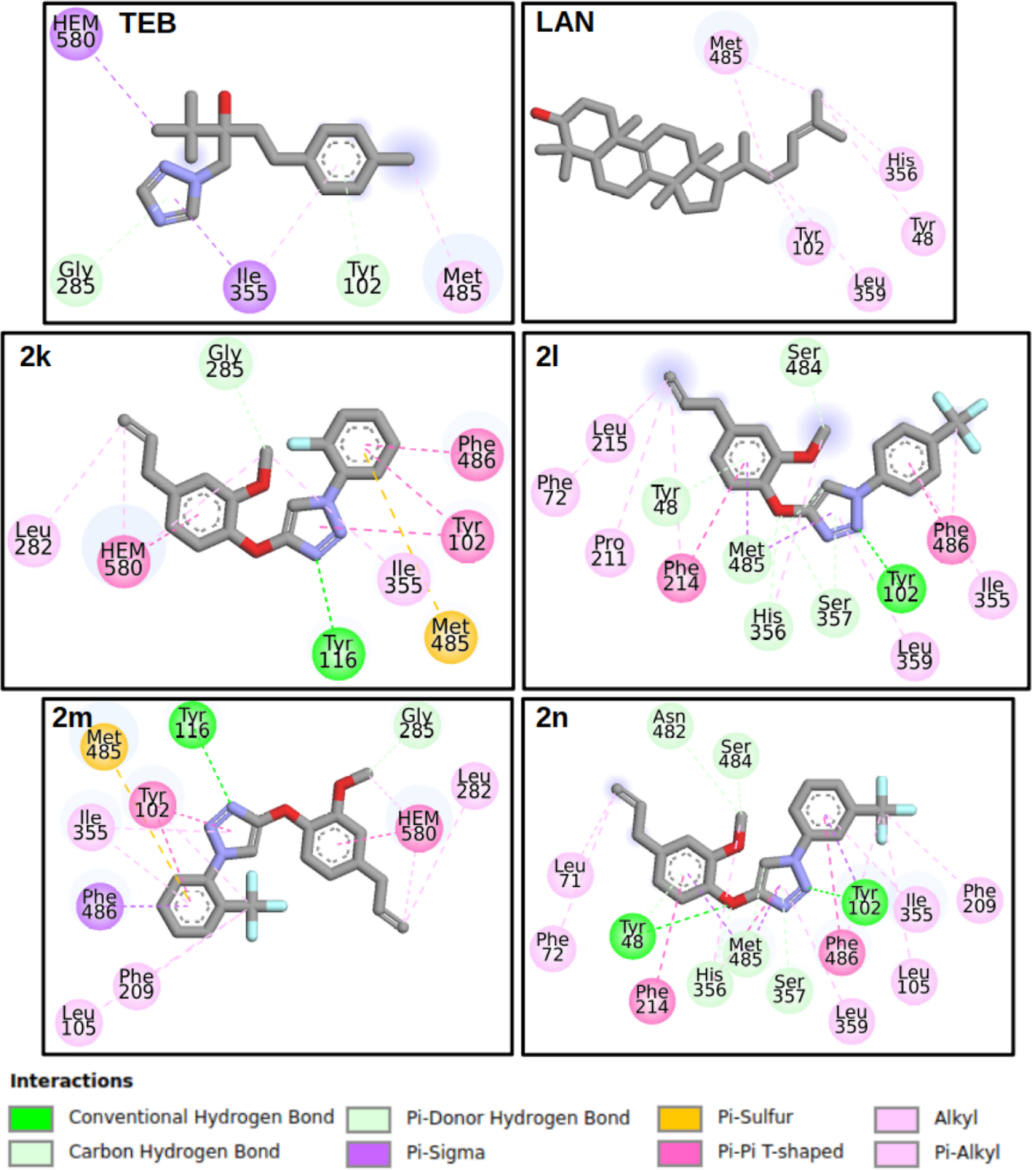
2D diagram illustrating the intermolecular interactions of the best docking poses for the antifungal TEB, substrate LAN, and compounds **2k**, **2l**, **2m**, and **2n**. The diagrams were created by using the Discovery Studio Visualizer.

Overall, most residues interacting with the compounds in each group are consistent due to the similarity in docked ligand poses (Figure 6). Polar residues, such as Tyr102 and Met485, interact with all derivatives, including **Teb** and **LAN** molecules. Within the apolar group, Ile355 and Phe486 interact with all of the molecules except for **2i** and **2b**, respectively. Therefore, these residues play a crucial role in interactions with the present derivatives, warranting consideration in molecular design based on our compounds.

For optimal visualization of the interactions between the ligands and the enzyme, Figure 6 presents the 2D interaction map for **Teb**, **LAN**, and triazoles **2k**, **2l**, **2m**, and **2n**. The complete set of 2D ligand interaction diagrams is presented in the Supporting Information (Figure S99).

As presented in Table S1 (Supporting Information), residue Met485 engages in interactions with all triazole derivatives, the antifungal **Teb**, and the substrate **LAN**. Specifically, Met485 forms a Pi–Sulfur interaction with the Ar ring (refer to Scheme 1 and Figure 5-**2k** and **2m**) in the G1 group. For the G2 group, it simultaneously interacts with the 1,2,3-triazole and eugenol rings through Pi–Sigma interactions (Figure 5-**2l** and **2n**). Within this group, another crucial residue is Tyr48, which interacts with the eugenol ring. Additionally, Tyr102 plays a significant role, forming a hydrogen bond with the derivative’s 1,2,3-triazole ring. Regarding apolar residues, Ile355 and Phe486 predominantly engage in Pi–Pi interactions with the aromatic ring. To summarize, the comprehensive CgCYP51A structure presented here holds potential value for future research aimed at designing potent inhibitors against *C. gloeosporioides*.

In summary, 1,2,3-triazole compounds derived from eugenol prove effective in reducing the mycelial growth of *C. gloeosporioides*, with **2k**, **2l**, **2m**, and **2n** exhibiting the highest efficiency. These new compounds could potentially be utilized in the management of papaya anthracnose, given their efficacy at low dosages against the disease’s etiological agent. Additionally, molecular docking calculations were conducted to elucidate the binding mode of the current derivatives in the catalytic pocket of *C. gloeosporioides* CYP51 (CgCYP51A). Our findings indicate that the seven best-docked compounds are situated near the heme cofactor, while the others bind in the channel access of the **LAN** substrate. The mechanism of action for these derivatives involves competition with the active region of CgCYP51A, preventing the entry of substrate **LAN**. Finally, we underscored the significance of residues Tyr102, Ile355, Met485, and Phe486 in their interactions with 1,2,3-triazole compounds.

## Supporting Information Available

Structural characterization data of compound **1** and triazoles **2a–2s**; 2. IR, NMR (^1^H AND ^13^C), and LC–MS/MS spectra of compounds **2a–2s**; complementary molecular docking data; mechanism of the cuaac reaction (PDF)
