# Supplementary material for: Design and Synthesis of Eugenol Derivatives Bearing a 1,2,3-Triazole Moiety for Papaya Protection against Colletotrichum gloeosporioides
Source: J Agric Food Chem. 2024 May 21;72(22):12459–68. doi: 10.1021/acs.jafc.4c00440 (PMC11157534; doi:10.1021/acs.jafc.4c00440)
Supplement: Supplementary file 1 — jf4c00440_si_001.pdf [file jf4c00440_si_001.pdf]

Supplementary Information

Design and Synthesis of Eugenol Derivatives Bearing a 1,2,3-Triazole Moiety for Papaya Protection against Colletotrichum gloeosporioides

Ângela Maria Almeida Lima[a§], Luíza Carvalheira Moreira[b§], Poliana Rodrigues Gazolla[a], Mariana Belizario Oliveira[a], Róbson Ricardo Teixeira[b*], Vagner Tebaldi Queiroz[a], Matheus Ricardo Rocha[c], Willian Bucker Moraes[c], Nayara Araújo dos Santos[d], Wanderson Romão[d], Valdemar Lacerda Jr.[d], Pedro Alves Bezerra Morais[a], Osmair Vital de Oliveira[e], Waldir Cintra de Jesus Júnior[f], Luiz C. A. Barbosa[g], Cláudia Jorge Nascimento[h], Jochen Junker[i] and Adilson Vidal Costa[a*]

[a] Departamento de Química e Física, Universidade Federal do Espírito Santo, Alto Universitário, s/n, Guararema, 29500-000 Alegre, ES, Brazil

[b] Departamento de Química, Universidade Federal de Viçosa, Av. P.H. Rolfs, s/n, 36570-900 Viçosa, MG, Brazil

[c] Departamento de Agronomia, Universidade Federal do Espírito Santo, Alto Universitário, s/n, Guararema, 29500-000 Alegre, ES, Brazil

[d] Laboratório de Petroleômica e Forense, Departamento de Química, Universidade Federal do Espírito Santo, Av. Fernando Ferrari 514, 29075-910 Vitória, ES, Brazil

[e] Instituto Federal de São Paulo, Campus Catanduva, 15808-305 Catanduva, São Paulo State, Brazil

[f] Universidade Federal de São Carlos, Campus Lagoa do Sino, 18290-000 Buri, SP, Brazil

[g] Departamento de Química, Universidade Federal de Minas Gerais, Av. Pres. Antônio Carlos 6627, 31270-901 Belo Horizonte, MG, Brazil

[h] Departamento de Ciências Naturais, Instituto de Biociências, Universidade Federal do Estado do Rio de Janeiro (UNIRIO), Av. Pauster, 22290-240, Rio de Janeiro, RJ, Brazil

[i] Centro de Desenvolvimento Tecnológico em Saúde, Fundação Oswaldo Cruz, Av. Brasil, 4365, 21040-900 Rio de Janeiro, RJ, Brazil

[§]These authors contributed equaly to this work.

*Corresponding authors: (e-mails: avcosta@hotmail.com; robsonr.teixeira@ufv.br)

ORCID: 0000-0002-7968-8586 (Adilson Vidal Costa); 0000-0003-3181-1108 (Róbson Ricardo Teixeira)

SUMMARY

	Page
1. STRUCTURAL CHARACTERIZATION DATA OF COMPOUND **1** AND TRIAZOLES **2a–2s**..	3
2. IR, NMR (^{1}H AND ^{13}C) AND LC-MS/MS SPECTRA OF COMPOUNDS **2a–2s**…	13
3. COMPLEMENTARY MOLECULAR DOCKING DATA..	62
4. MECHANISM OF THE CuAAC REACTION..	68

1. STRUCTURAL CHARACTERIZATION DATA OF COMPOUND 1 AND TRIAZOLES 2a-2s

Data for 4-allyl-2-methoxy-1-(prop-2-yn-1-yloxy)benzene (**1**)

Compound **1** was obtained as a yellow oil in 81.0% yield (1.20 g, 7.30 mmol). The structure of **1** was secured by the following data. TLC: R_f = 0.65 (hexane/ethyl acetate 4:1 v v^{-1}); IR (ATR) ν / cm^{-1} 3291, 3076, 3002, 2935, 2905, 2834, 1638, 1594, 1507, 1452, 1419, 1374, 1334, 1257, 1214, 1138, 1023, 995, 914, 851, 802, 750; ^1H NMR (300 MHz, $CDCl_3$) δ 2.49 (t, 1H, J = 2.4 Hz), 3.35 (d, 2H, J = 6.7 Hz), 3.86 (s, 3H), 4.73 (d, 2H, J = 2.4 Hz), 5.06-5.12 (m, 2H), 5.96 (ddt, 1H, J = 16.8, 10.2, 6.7 Hz), 6.72─6.74 (m, 2H), 7.00 (d, 1H, J = 8.6 Hz); ^{13}C NMR (75 MHz, $CDCl_3$) δ 39.8, 55.8, 56.9, 75.5, 78.7, 112.3, 114.6, 115.7, 120.3, 134.2, 137.4, 145.0, 149.6.

Data for 4-((4-allyl-2-methoxyphenoxy)methyl)-1-(4-bromophenyl)-1H-1,2,3-triazole (***2a***)

Compound **2a** was obtained as a white solid in 87.6% yield (0.135 g, 0.337 mmol) from 0.076 g (0.385 mmol) of 1-azido-4-bromobenzene, 0.078 g (0.385 mmol) of **1**, 0.030 g (0.154 mmol) of sodium ascorbate and 0.019 g (0.077 mmol) of $CuSO_4{\cdot}5H_2O$. Reaction time: 24 hours. TLC: R_f = 0.37 (hexane/ethyl acetate 4:1 v v^{-1}); mp 124.3─124.8 °C; IR (ATR) ν / cm^{-1} 3128, 3094, 2957, 2924, 2871, 1638, 1590, 1510, 1493, 1462, 1259, 1230, 1138, 1068, 1034, 1025, 987, 926, 824, 804, 749, 650, 605, 517, 453; ^1H NMR (600 MHz, DMSO-d_6) δ 3.29 (d, 2H, J = 6.6 Hz), 3.73 (s, 3H), 5.08─5.01 (m, 2H), 5.16 (s, 2H), 5.96 (ddt, 1H, J = 6.6, 9.6, 16.8 Hz), 6.70─6.69 (m, 1H), 6.81─6.80 (m, 1H), 7.07 (d, 1H, J = 7.8 Hz), 7.80 (d, 2H, J = 9.0 Hz), 7.90 (d, 2H, J = 9.0 Hz), 8.93 (s, 1H); ^{13}C NMR (150 MHz, DMSO-d_6) δ 39.5, 55.8, 62.2, 113.0, 114.6, 116.0, 120.6, 121.8, 122.4, 123.3, 133.2, 133.6, 136.2, 138.2, 144.6, 146.1, 149.5; LC-MS (ESI) *m/z*, calcd. for $C_{19}H_{18}BrN_3NaO_2$ $[M + Na]^+$: 422.05, found: 422.07.

Data for 4-((4-allyl-2-methoxyphenoxy)methyl)-1-(2-bromophenyl)-1H-1,2,3-triazole (***2b***)

Compound **2b** was obtained as a white solid in 73.0% yield (0.143 g, 0.360 mmol) from 0.098 g (0.494 mmol) of 1-azido-2-bromobenzene, 0.100 g (0.494 mmol) of the alkyne **1**, 0.039 g (0.198

mmol) of sodium ascorbate and 0.025 g (0.099 mmol) of $CuSO_4{\cdot}5H_2O$. Reaction time: 24 hours. TLC: R_f = 0.72 (hexane/ethyl acetate 3:2 v v^{-1}); mp 64.6 - 65.4 °C; IR (ATR) ν / cm^{-1} 3135, 3072, 2935, 2868, 2838, 1634, 1588, 1508, 1456, 1378, 1256, 1222, 1139, 1021, 1009, 995, 913, 845, 802, 753, 640, 605, 553, 450; ^1H NMR (600 MHz, DMSO-d_6) δ 3.30 (d, 2H, J = 6.6 Hz), 3.74 (s, 3H), 5.02─5.09 (m, 2H), 5.17 (s, 2H), 5.96 (ddt, 1H, J = 6.6, 10.2, 16.8 Hz), 6.71 (dd, 1H, J = 1.8, 8.4 Hz), 6.81─6.82 (m, 1H), 7.08 (d, 1H, J = 8.4 Hz), 7.56 (td, 1H, J = 1.8, 7.8 Hz), 7.63 (td, 1H, J = 1.2, 7.8 Hz), 7.65 (dd, 1H, J = 1.8, 7.8 Hz), 7.91 (d, 1H, J = 9.0 Hz), 8.62 (s, 1H); ^{13}C NMR (150 MHz, DMSO-d_6) δ 39.5, 55.8, 62.2, 113.0, 114.9, 115.9, 119.2, 120.6, 127.2, 129.1, 129.3, 132.4, 133.6, 134.0, 136.5, 138.3, 143.3, 146.1, 149.6; LC-MS (ESI) *m/z*, calcd. for $C_{19}H_{18}$ BrN_3NaO_2 [M + Na]$^+$: 422.05, found: 422.05.

Data for 4-((4-allyl-2-methoxyphenoxy)methyl)-1-(3-bromophenyl)-1H-1,2,3-triazole ***(2c)*** Compound **2c** was obtained as a light brown solid in 70.0% yield (0.38 g, 0.346 mmol) from 0.098 g (0.494 mmol) of 1-azido-3-bromobenzene, 0.100 g (0.494 mmol) of the alkyne **1**, 0.040 g (0.200 mmol) of sodium ascorbate and 0.025 g (0.098 mmol) of $CuSO_4{\cdot}5H_2O$. Reaction time: 24 hours. TLC: R_f = 0.50 (hexane/ethyl acetate 4:1 v v^{-1}); mp 102.2─102.6 °C; IR (ATR) ν / cm^{-1} 3139, 3076, 2991, 2916, 2849, 1679, 1634, 1588, 1513, 1493, 1460, 1423, 1337, 1258, 1235, 1211, 1136, 1046, 1027, 992, 911, 865, 780, 675, 646, 598, 542, 438; ^1H NMR (600 MHz, DMSO-d_6) δ 3.28 (d, 2H, J = 6.6 Hz), 3.71 (s, 3H), 5.99-5.06 (m, 2H), 5.14 (s, 2H), 5.88-5.95 (m, 1H), 6.68 (d, 1H, J = 8.4 Hz), 6.78 (s, 1H), 7.04 (d, 1H, J = 8.4 Hz), 7.54 (t, 1H, J = 8.4, 7.8 Hz), 7.68 (d, 1H, J = 8.4 Hz), 7.94 (d, 1H, J = 8.4 Hz), 8.15 (s, 1H), 8.96 (s, 1H); ^{13}C NMR (150 MHz, DMSO-d_6) δ 39.5, 55.8, 62.2, 113.0, 114.7, 116.0, 119.5, 120.6, 122.8, 123.1, 123.5, 131.9, 132.3, 133.6, 138.1, 138.3, 144.6, 146.1, 149.6; LC-MS (ESI) *m/z*, calcd. for $C_{19}H_{18}$ BrN_3NaO_2 [M + Na]$^+$: 422.05, found: 422.13.

Data for 4-((4-allyl-2-methoxyphenoxy)methyl)-1-(4-chlorophenyl)-1H-1,2,3-triazole ***(2d)*** Compound **2d** was obtained as a white solid in 83.0% yield (0.145 g, 0.410 mmol) from 0.076 g

(0.494 mmol) of 1-azido-4-chlorobenzene, 0.100 g (0.494 mmol) of the alkyne **1**, 0.040 g (0.200 mmol) of sodium ascorbate and 0.025 g (0.098 mmol) of $CuSO_4·5H_2O$. Reaction time: 24 hours. TLC: $R_f = 0.48$ (hexane/ethyl acetate 4:1 v v^{-1}); mp 124.8-125.3 °C; IR (ATR) ν / cm^{-1} 3132, 3098, 2998, 2924, 2875, 2834, 1638, 1590, 1510, 1497, 1463, 1404, 1337, 1260, 1231, 1218, 1140, 1090, 1036, 1026, 992, 926, 826, 751, 648, 605, 520, 470; ^1H NMR (600 MHz, DMSO-d_6) δ 3.29 (d, 2H, J = 6.6 Hz), 3.73 (s, 3H), 5.01-5.08 (m, 2H), 5.16 (s, 2H), 5.96 (ddt, 1H, J = 6.6, 10.2, 16.8 Hz), 6.69-6.70 (m, 1H), 6.80–6.81 (m, 1H), 7.07 (d, 1H, J = 7.8 Hz), 7.67 (d, 2H, J = 9.0 Hz), 7.95 (d, 2H, J = 9.0 Hz), 8.94 (s, 1H); ^{13}C NMR (150 MHz, DMSO-d_6) δ 39.5, 55.8, 62.2, 113.0, 114.7, 116.0, 120.6, 122.2, 123.4, 130.3, 133.4, 133.6, 135.8, 138.3, 144.6, 146.1, 149.5; LC-MS (ESI) *m/z*, calcd. for $C_{19}H_{18}ClN_3NaO_2$ $[M + Na]^+$: 378.10, found: 378.09.

Data for 4-((4-allyl-2-methoxyphenoxy)methyl)-1-(3-chlorophenyl)-1H-1,2,3-triazole ***(2e)*** Compound **2e** was obtained as a white solid in 76.0% yield (0.133 g, 0.375 mmol) from 0.076 g (0.494 mmol) of 1-azido-3-chlorobenzene, 0.100 g (0.494 mmol) of the alkyne **1**, 0.040 g (0.200 mmol) of sodium ascorbate and 0.025 g (0.098 mmol) of $CuSO_4·5H_2O$. Reaction time: 24 hours. TLC: $R_f = 0.46$ (hexane/ethyl acetate 4:1 v v^{-1}); mp 104.2–104.8 °C; IR (ATR) ν / cm^{-1} 3143, 3076, 2991, 2912, 2831, 1638, 1594, 1514, 1496, 1460, 1423, 1259, 1235, 1211, 1136, 1030, 993, 911, 866, 781, 753, 781, 676, 646, 598, 546, 442; ^1H NMR (600 MHz, DMSO-d_6) δ 3.30 (d, 2H, J = 7.2 Hz), 3.73 (s, 3H), 5.01–5.08 (m, 2H), 5.16 (s, 2H), 5.96 (ddt, 1H, J = 6.6, 9.6, 16.8 Hz), 6.70 (dd, 1H, J = 2.4, 8.4 Hz), 6.80-6.81 (m, 1H), 7.06 (d, 1H, J = 8.4 Hz), 7.56-7.57 (m, 1H), 7.63 (t, 1H, J = 7.8, 8.4 Hz), 7.93-7.94 (m, 1H), 8.05–8.06 (m, 1H), 8.99 (s, 1H); ^{13}C NMR (150 MHz, DMSO-d_6) δ 41.7, 58.0, 64.4, 115.2, 116.9, 118.1, 121.3, 122.5, 122.7, 125.7, 131.1, 134.2, 135.8, 136.8, 140.2, 140.4, 146.8, 148.2, 151.7; LC-MS (ESI) *m/z*, calcd. for $C_{19}H_{18}ClN_3NaO_2$ $[M + Na]^+$: 378.10, found: 378.16.

Data for 4-((4-allyl-2-methoxyphenoxy)methyl)-1-(2-chlorophenyl)-1H-1,2,3-triazole ***(2f)*** Compound **2f** was obtained as a pasty white solid in 68.0% yield (0.120 g, 0.336 mmol) from 0.076

g (0.494 mmol) of 1-azido-2-chlorobenzene, 0.100 g (0.494 mmol) of the alkyne **1**, 0.040 g (0.200 mmol) of sodium ascorbate and 0.025 g (0.098 mmol) of $CuSO_4 \cdot 5H_2O$. Reaction time: 24 hours. TLC: $R_f = 0.54$ (hexane/ethyl acetate 3:2 v v^{-1}); IR (ATR) ν / cm^{-1} 3143, 3076, 3002, 2935, 2871, 2834, 1716, 1638, 1590, 1508, 1496, 1459, 1419, 1257, 1224, 1138, 1035, 1017, 911, 847, 804, 756, 647, 602, 546, 459; ^1H NMR (400 MHz, $CDCl_3$) δ 3.34 (d, 2H), 3.86 (s, 3H), 5.06-5.10 (m, 2H), 5.38 (s, 2H), 5.90-6.00 (m, 1H), 6.71–6.73 (m, 2H), 7.01 (d, 1H), 7.44-7.46 (m, 2H), 7.56–7.62 (m, 2H), 8.08 (s, 1H); ^{13}C NMR (100 MHz, $CDCl_3$) δ 40.0, 56.0, 63.5, 112.6, 115.2, 115.9, 120.7, 125.3, 128.0, 128.1, 131.0, 131.0, 134.3, 137.7, 144.4, 146.0, 149.9; LC-MS (ESI) *m/z*, calcd. for $C_{19}H_{18}ClN_3NaO_2$ $[M + Na]^+$: 378.10, found: 378.10.

*Data for 4-((4-allyl-2-methoxyphenoxy)methyl)-1-(m-tolyl)-1H-1,2,3-triazole (**2g**)*

Compound **2g** was obtained as a pasty yellow solid in 67% yield (0.116 g, 0.346 mmol) from 0.068 g (0.514 mmol) of 1-azido-3-methylbenzene, 0.104 g (0.514 mmol) of the alkyne **1**, 0.040 g (0.206 mmol) of sodium ascorbate and 0.026 g (0.103 mmol) of $CuSO_4 \cdot 5H_2O$. Reaction time: 24 hours. TLC: $R_f = 0.32$ (hexane/ethyl acetate 4:1 v v^{-1}); IR (ATR) ν / cm^{-1} 3139, 3080, 2976, 2931, 2905, 2831, 1638, 1612, 1591, 1512, 1459, 1419, 1341, 1258, 1233, 1136, 1044, 1030, 1010, 992, 900, 783, 684, 646, 598, 549, 438; ^1H NMR (600 MHz, DMSO-d_6) δ 2.41 (s, 3H), 3.30 (d, 2H, J = 6.6 Hz), 3.73 (s, 3H), 5.02–5.09 (m, 2H), 5.16 (s, 2H), 5.96 (ddt, 1H, J = 6.6, 10.2, 16.8 Hz), 6.69-6.71 (m, 1H), 6.81-6.82 (m, 1H), 7.07 (d, 1H, J = 7.8 Hz), 7.31 (d, 1H, J = 7.8 Hz), 7.47 (t, 1H, J = 8.1 Hz), 7.69 (d, 1H, J = 8.4 Hz), 7.75 (s, 1H), 8.80 (s, 1H); ^{13}C NMR (150 MHz, DMSO-d_6) δ 23.5, 41.7, 58.0, 64.4, 115.2, 116.6, 118.1, 119.8, 122.7, 123.1, 125.4, 131.9, 132.3, 135.7, 139.1, 140.5, 142.2, 146.5, 148.3, 151.7; LC-MS (ESI) *m/z*, calcd. for $C_{20}H_{21}N_3NaO_2$ $[M + Na]^+$: 358.15, found: 358.22.

*Data for 4-((4-allyl-2-methoxyphenoxy)methyl)-1-(p-tolyl)-1H-1,2,3-triazole (**2h**)*

Compound **2h** was obtained as a pasty white solid in 62.0% yield (0.102 g, 0.306 mmol) from 0.066 g (0.494 mmol) of 1-azido-4-methylbenzene, 0.100 g (0.494 mmol) of the alkyne **1**, 0.040 g (0.200

mmol) of sodium ascorbate and 0.025 g (0.098 mmol) of $CuSO_4·5H_2O$. Reaction time: 24 hours. TLC: R_f = 0.44 (hexane/ethyl acetate 3:2 v v^{-1}); IR (ATR) ν / cm^{-1} 3276, 3132, 3080, 2924, 2871, 1739, 1672, 1591, 1510, 1463, 1408, 1322, 1259, 1219, 1138, 1026, 921, 817, 761, 650, 602, 521, 457; ^{1}H NMR (400 MHz, $CDCl_3$) δ 2.44 (s, 3H), 3.35 (d, 2H, J = 6.4 Hz), 3.88 (s, 3H), 5.07─5.12 (m, 2H), 5.38 (s, 2H), 5.92-6.02 (m, 1H), 6.72─6.75 (m, 2H), 7.02 (d, 1H, J = 8.0 Hz), 7.33 (d, 2H, J = 8.0 Hz), 7.61 (d, 2H, J = 8.0 Hz), 8.09 (s, 1H); ^{13}C NMR (100 MHz, $CDCl_3$) δ 21.1, 39.8, 55.8, 63.2, 112.3, 114.5, 115.7, 120.5, 120.6, 121.3, 130.3, 134.0, 134.5, 137.4, 139.2, 144.8, 145.8, 149.5; LC-MS (ESI) *m/z*, calcd. for $C_{20}H_{21}N_3NaO_2$ $[M + Na]^+$: 358.15, found: 358.21.

*Data for 4-((4-allyl-2-methoxyphenoxy)methyl)-1-phenyl-1H-1,2,3-triazole (**2i**)*

Compound **2i** was obtained as a white solid in 62% yield (0.098 g, 0.305 mmol) from 0.058 g (0.494 mmol) of azidobenzene, 0.100 g (0.494 mmol) of the alkyne **1**, 0.039 g (0.197 mmol) of sodium ascorbate and 0.025 g (0.099 mmol) of $CuSO_4·5H_2O$. Reaction time: 48 hours. TLC: R_f = 0.32 (hexane/ethyl acetate 4:1 v v^{-1}); mp 78.3─78.9 °C; IR (ATR) ν / cm^{-1} 3135, 3080, 2994, 2935, 2905, 2827, 1638, 1592, 1501, 1463, 1423, 1385, 1330, 1258, 1222, 1139, 1033, 1001, 983, 913, 834, 762, 689, 650, 598, 518, 464; ^{1}H NMR (300 MHz, $CDCl_3$) δ 3.33 (d, 2H, J = 6.9 Hz), 3.86 (s, 3H), 5.04─5.10 (m, 2H), 5.35 (s, 2H), 5.98 (ddt, 1H, J = 16.8, 10.2, 6.9 Hz), 6.70─6.73 (m, 2H), 7.00 (d, 1H, J = 7.8 Hz), 7.40─7.54 (m, 3H), 7.71 (d, 2H, J = 8.1 Hz), 8.07 (s, 1H); ^{13}C NMR (75 MHz, $CDCl_3$) δ 39.8, 55.8, 63.3, 112.3, 114.4, 115.7, 120.5, 120.5, 121.0, 128.8, 129.7, 133.9, 136.9, 137.4, 145.2, 145.8, 149.5; LC-MS (ESI) *m/z*, calcd. for $C_{19}H_{19}N_3NaO_2$ $[M + Na]^+$: 344.14, found: 344.22.

*Data for 4-((4-allyl-2-methoxyphenoxy)methyl)-1-(4-fluorophenyl)-1H-1,2,3-triazole (**2j**)*

Compound **2j** was obtained as a white solid in 69% yield (0.073 g, 0.215 mmol) from 0.043 g (0.311 mmol) of 1-azido-4-fluorobenzene, 0.063 g (0.311 mmol) of the alkyne **1**, 0.025 g (0.012 mmol) of sodium ascorbate and 0.0016 g (0.062 mmol) of $CuSO_4·5H_2O$. Reaction time: 24 hours. TLC: R_f = 0.51 (hexane/ethyl acetate 3:2 v v^{-1}); mp 80.4-1.2 °C; IR (ATR) ν / cm^{-1} 3124, 3065,

3005, 2935, 2879, 2831, 1638, 1592, 1510, 1463, 1420, 1378, 1222, 1138, 1051, 1031, 1003, 995, 911, 836, 799, 761, 698, 646, 602, 518, 479; ^{1}H NMR (600 MHz, DMSO-d_6) δ 3.30 (d, 2H, J = 7.2 Hz), 3.73 (s, 3H), 5.01—5.08 (m, 2H), 5.16 (s, 2H), 5.96 (ddt, 1H, J = 7.2, 10.2, 16.8 Hz), 6.70 (ddap, 1H, J = 1.8, 7.8 Hz), 6.80—6.81 (m, 1H), 7.07 (d, 1H, J = 8.4 Hz), 7.46 (t, 2H, J = 8.7 Hz), 7.93 (dd, 2H, J = 4.8, 9.0 Hz), 8.89 (s, 1H); ^{13}C NMR (150 MHz, DMSO-d_6) δ 39.5, 55.8, 62.2, 113.0, 114.6, 116.0, 117,1 (d, J = 9.0 Hz), 120.6, 122.9 (d, J = 9.0 Hz), 123.5, 133.5, 133.6, 138.3, 144.5, 146.1, 149.5, 162.1 (d, J = 244.5 Hz); LC-MS (ESI) *m/z*, calcd. for $C_{19}H_{18}FN_3NaO_2$ [M + Na]$^+$: 362.13, found: 362.15.

Data for 4-((4-allyl-2-methoxyphenoxy)methyl)-1-(2-fluorophenyl)-1H-1,2,3-triazole ***(2k)***
Compound **2k** was obtained as a pasty white solid in 77% yield (0.193 g, 0.568 mmol) from 0.101 g (0.741 mmol) of 1-azido-2-fluorobenzene, 0.150 g (0.741 mmol) of the alkyne **1**, 0.058 g (0.296 mmol) of sodium ascorbate and 0.037 g (0.148 mmol) of $CuSO_4 \cdot 5H_2O$. Reaction time: 24 hours. TLC: R_f = 0.22 (hexane/ethyl acetate 4:1 v v^{-1}); IR (ATR) ν / cm^{-1} 3143, 3083, 2998, 2935, 2834, 1638, 1588, 1508, 1463, 1404, 1334, 1258, 1228, 1136, 1108, 1050, 1035, 1018, 991, 906, 851, 806, 704, 646, 553, 475; ^{1}H NMR (600 MHz, DMSO-d_6) δ 3.30 (d, 2H, J = 6.6 Hz), 3.73 (s, 3H), 5.02—5.09 (m, 2H), 5.17 (s, 2H), 5. 96 (ddt, 1H, J = 6.6, 10.2, 16.8 Hz), 6.71 (dd, 1H, J = 1.8, 8.4 Hz), 6.80—6.81 (m, 1H), 7.09 (d, 1H, J = 8.4 Hz), 7.43—7.46 (m, 1H), 7.59—7.64 (m, 2H), 7.83—7.86 (m, 1H), 8.69—8.70 (m, 1H); ^{13}C NMR (150 MHz, DMSO-d_6) δ 39.5, 55.8, 62.0, 113.0, 114.6, 116.0, 117.5 (d, J = 19.5 Hz), 120.6, 125.1 (d, J = 10.5 Hz), 126.0 (d, J = 3.0 Hz), 126.4, 126.6 (d, J = 4.5 Hz), 131.7 (d, J = 9.0 Hz), 133.5, 138.3, 143.9, 146.1, 149.5, 154.3 (d, J = 249 Hz); LC-MS (ESI) *m/z*, calcd. for $C_{19}H_{18}FN_3NaO_2$ [M + Na]$^+$: 362.13, found: 362.16.

Data for 4-((4-allyl-2-methoxyphenoxy)methyl)-1-(4-(trifluoromethyl)phenyl)-1H-1,2,3-triazole ***(2l)***
Compound **2l** was obtained as a white solid in 72% yield (0.138 g, 0.354 mmol) from 0.092 g (0.494 mmol) of 1-azido-4-(trifluoromethyl)benzene, 0.100 g (0.494 mmol) of the alkyne **1**, 0.039 g (0.197 mmol) of sodium ascorbate and 0.025 g (0.099 mmol) of $CuSO_4 \cdot 5H_2O$. Reaction time: 24

hours. TLC: R_f = 0.33 (hexane/ethyl acetate 3:2 v v^{-1}); mp 90.0-90.6 °C; IR (ATR) ν / cm^{-1} 3091, 2924, 2849, 1638, 1616, 1590, 1511, 1463, 1415, 1319, 1259, 1229, 1170, 1130, 1110, 1066, 1021, 921, 843, 691, 650, 598, 520, 460; ^{1}H NMR (600 MHz, DMSO-d_6) δ 3.30 (d, 2H, J = 6.6 Hz), 3.73 (s, 3H), 5.01–5.08 (m, 2H), 5.18 (s, 2H), 5.96 (ddt, 1H, J = 6.6, 10.2, 16.8 Hz), 6.70 (dd, 1H, J = 1.8, 7.8 Hz), 6.81-6.82 (m, 1H), 7.07 (d, 1H, J = 7.8 Hz), 8.00 (d, 2H, J = 8.4 Hz), 8.18 (d, 2H, J = 8.4 Hz), 9.07 (s, 1H);^{13}C NMR (150 MHz, DMSO-d_6) δ 39.5, 55.8, 62.2, 113.0, 114.7, 116.0, 120.6, 121.0, 123.6, 124.2 (q, J = 270 Hz), 127.6 (q, J = 3.5 Hz), 129.1 (q, J = 31.5 Hz), 133.6, 138.3, 139.7, 144.8, 146.1, 149.6; LC-MS (ESI) *m/z*, calcd. for $C_{20}H_{18}F_3N_3NaO_2$ $[M + Na]^+$: 412.12, found: 412.29.

*Data for 4-((4-allyl-2-methoxyphenoxy)methyl)-1-(2-(trifluoromethyl)phenyl)-1H-1,2,3-triazole (**2m**)*

Compound **2m** was obtained as a white solid in 88.0% yield (0.103 g, 0.264 mmol) from 0.056 g (0.301 mmol) of 1-azido-2-(trifluoromethyl)benzene, 0.061 g (0.301 mmol) of the alkyne **1**, 0.024 g (0.120 mmol) of sodium ascorbate and 0.015 g (0.060 mmol) of $CuSO_4 \cdot 5H_2O$. Reaction time: 24 hours. TLC: R_f = 0.52 (hexane/ethyl acetate 3:2 v v^{-1}); mp 89.0-89.7 °C; IR (ATR) ν / cm^{-1} 3132, 3094, 3009, 2972, 2942, 2868, 2842, 1638, 1612, 1590, 1510, 1467, 1419, 1374, 1317, 1263, 1227, 1189, 1020, 902, 851, 768, 694, 639, 598, 549, 460; ^{1}H NMR (600 MHz, DMSO-d_6) δ 3.30 (d, 2H, J = 7.2 Hz), 3.74 (s, 3H), 5.02-5.08 (m, 2H), 5.17 (s, 2H), 5.96 (ddt, 1H, J = 6.6, 10.2, 16.8 Hz), 6.70 (dd, 1H, J = 1.8, 6.0 Hz), 6.81-6.82 (m, 1H), 7.06 (d, 1H, J = 8.4 Hz), 7.73 (d, 1H, J = 7.8 Hz), 7.86 (t, 1H, J = 7.8 Hz), 7.93 (t, 1H, J = 7.8 Hz), 8.03 (d, 1H, J = 9.0 Hz), 8.61 (s, 1H); ^{13}C NMR (150 MHz, DMSO-d_6) δ 39.5, 55.8, 62.2, 113.1, 115.0, 115.9, 120.6, 123.1 (q, J = 271.5 Hz), 125.2 (q, J = 31.0 Hz), 127.8 (q, J = 5.0 Hz), 129.7, 131.6, 133.6, 134.4, 134.6 (q, J = 1.5 Hz), 138.3, 143.5, 146.1, 149.6; LC-MS (ESI) *m/z*, calcd. for $C_{20}H_{18}F_3N_3NaO_2$ $[M + Na]^+$: 412.12, found: 412.10.

*Data for 4-((4-allyl-2-methoxyphenoxy)methyl)-1-(3-(trifluoromethyl)phenyl)-1H-1,2,3-triazole (**2n**)*

Compound **2n** was obtained as a white solid in 86% yield (0.166 g, 0.426 mmol) from 0.092 g

(0.494 mmol) of 1-azido-3-(trifluoromethyl)benzene, 0.100 g (0.494 mmol) of the alkyne **1**, 0.039 g (0.198 mmol) of sodium ascorbate and 0.025 g (0.099 mmol) of $CuSO_4{\cdot}5H_2O$. Reaction time: 24 hours. TLC: $R_f = 0.29$ (hexane/ethyl acetate 4:1 v v^{-1}); mp 117.9-118.6 °C; IR (ATR) ν / cm^{-1} 3180, 3080, 3020, 2968, 2909, 2871, 2838, 1638, 1601, 1513, 1460, 1404, 1328, 1300, 1261, 1231, 1168, 1120, 1070, 1031, 995, 914, 895, 795, 698, 650, 602, 546, 457; ^{1}H NMR (300 MHz, $CDCl_3$) δ 3.33 (d, 2H, J = 6.6 Hz), 3.87 (s, 3H), 5.04-5.11 (m, 2H), 5.35 (s, 2H), 6.00 (ddt, 1H, J = 17.1, 10.2, 6.9 Hz), 6.70-6.74 (m, 2H), 6.98 (d, 1H, J = 7.8 Hz), 7.63-7.71 (m, 2H), 7.93-7.95 (m, 1H), 8.01 (brs, 1H), 8.13 (s, 1H); ^{13}C NMR (75 MHz, $CDCl_3$) δ 39.8, 55.8, 63.3, 112.3, 114.4, 115.7, 117.4 (q, J = 3.75 Hz), 120.5, 120.8, 123.5 (d, J = 271.5 Hz), 125.3 (q, J = 3.5 Hz), 130.5, 132.4 (q, J = 33.0 Hz), 134.1, 137.3, 137.4, 145.7, 145.8, 149.5; LC-MS (ESI) *m/z*, calcd. for $C_{20}H_{18}F_3N_3NaO_2$ [M + Na]$^+$: 412.12, found: 412.23.

*Data for 4-((4-allyl-2-methoxyphenoxy)methyl)-1-(4-methoxyphenyl)-1H-1,2,3-triazole **(2o)***
Compound **2o** was obtained as a white solid in 33% yield (0.072 g, 0.205 mmol) from 0.093 g (0.623 mmol) of 1-azido-4-methoxybenzene, 0.126 g (0,623 mmol) of the alkyne **1**, 0.049 g (0.250 mmol) of sodium ascorbate and 0.031 g (0.125 mmol) of $CuSO_4{\cdot}5H_2O$. Reaction time: 24 hours. TLC: $R_f = 0.52$ (hexane/ethyl acetate 3:2 v v^{-1}); mp 91.1-91.8 °C; IR (ATR) ν / cm^{-1} 3135, 3080, 2994, 2942, 2920, 2883, 2846, 1638, 1594, 1513, 1463, 1404, 1334, 1304, 1252, 1227, 1139, 1032, 925, 832, 819, 812, 758, 650, 605, 530, 457; ^{1}H NMR (400 MHz, $CDCl_3$) δ 3.35 (d, 2H, J = 6,4 Hz), 3.88 (s, 6H), 5.07-5.12 (m, 2H), 5.36 (s, 2H), 5.92-6.02 (m, 1H), 6.72-6.75 (m, 2H), 7.03 (d, 3H, J = 8.8 Hz), 7.63 (d, 2H), 8.03 (s, 1H); ^{13}C NMR (100 MHz, $CDCl_3$) δ 39.8, 55.6, 55.8, 63.2, 112.3, 114.4, 114.8, 115.7, 120.5, 121.3, 122.2, 130.3, 133.9, 137.3, 144.8, 145.8, 149.5, 159.9; LC-MS (ESI) *m/z*, calcd. for $C_{20}H_{21}N_3NaO_3$ [M + Na]$^+$: 374.15, found: 374.22.

*Data for 4-((4-allyl-2-methoxyphenoxy)methyl)-1-(2-methoxyphenyl)-1H-1,2,3-triazole **(2p)***
Compound **2p** was obtained as a pasty white solid in 59% yield (0.086 g, 0.245 mmol) from 0.062 g (0.415 mmol) of 1-azido-2-methoxybenzene, 0.084 g (0.415 mmol) of the alkyne **1**, 0.033 g (0.166

mmol) of sodium ascorbate and 0.021 g (0.083 mmol) of $CuSO_4·5H_2O$. Reaction time: 24 hours. TLC: R_f = 0.58 (hexane/ethyl acetate 3:2 v v^{-1}); IR (ATR) ν / cm^{-1} 3158, 3080, 3050, 2964, 2938, 2879, 2842, 1669, 1590, 1507, 1452, 1389, 1341, 1247, 1219, 1132, 1120, 1073, 1010, 986, 906, 854, 802, 747, 709, 657, 587, 546, 460; ^1H NMR (600 MHz, DMSO-d_6) δ 3.30 (d, 2H, *J* = 7.2 Hz), 3.73 (s, 3H), 3.85 (s, 3H), 5.02-5.09 (m, 2H), 5.15 (s, 2H), 5.96 (ddt, 1H, *J* = 6.6, 10.2, 16.8 Hz), 6.71 (dd, 1H, *J* = 1.8, 8.4 Hz), 6.80-6.81 (m, 1H), 7.09 (d, 1H, *J* = 7.8 Hz), 7.16 (td, 1H, *J* = 1.2, 7.8 Hz), 7.31-7.33 (m, 1H), 7.52-7.55 (m, 1H), 7.63 (dd, 1H, *J* = 1.8, 7.8 Hz), 8.53 (s, 1H);^{13}C NMR (150 MHz, DMSO-d_6) δ 39.5, 55.8, 56.5, 62.1, 113.0, 113.4, 114.5, 115.9, 120.6, 121.3, 126.0, 126.2, 127.0, 131.2, 133.4, 138.3, 143.0, 146.2, 149.5, 152.0; LC-MS (ESI) *m/z*, calcd. for $C_{20}H_{21}N_3NaO_3$ [M + Na]$^+$: 374.15, found: 374.17.

*Data for 4-((4-allyl-2-methoxyphenoxy)methyl)-1-(3-methoxyphenyl)-1H-1,2,3-triazole (**2q**)* Compound **2q** was obtained as a pasty yellow solid in 62% yield (0.054 g, 0.154 mmol) from 0.037 g (0.247 mmol) of 1-azido-3-methoxybenzene, 0.050 g (0.247 mmol) of the alkyne **1**, 0.020 g (0.099 mmol) of sodium ascorbate and 0.012 g (0.049 mmol) of $CuSO_4·5H_2O$. Reaction time: 24 hours. TLC: R_f = 0.61 (hexane/ethyl acetate 3:2 v v^{-1}); IR (ATR) ν / cm^{-1} 3176, 3124, 3080, 2968, 2931, 2834, 1752, 1675, 1607, 1594, 1505, 1453, 1419, 1337, 1300, 1253, 1214, 1159, 1132, 1047, 1028, 993, 911, 851, 770, 680, 572, 460; ^1H NMR (600 MHz, DMSO-d_6) δ 3.30 (d, 2H, *J* = 6.6 Hz), 3.73 (s, 3H), 3.85 (s, 3H), 5.01-5.08 (m, 2H), 5.15 (s, 2H), 5.96 (ddt, 1H, *J* = 7.2, 10.2, 16.8 Hz), 6.70 (dd, 1H, *J* = 1.8, 8.4 Hz), 6.80-6.81 (m, 1H), 7.05–7.08 (m, 2H), 7.47-7.50 (m, 3H), 8.94 (s, 1H); ^{13}C NMR (150 MHz, DMSO-d_6) δ 39.5, 55.8, 56.0, 62.2, 106.1, 112.5, 113.0, 114.6, 114.9, 116.0, 120.6, 123.4, 131.3, 133.6, 138.0, 138.2, 144.4, 146.0, 149.5, 160.6; LC-MS (ESI) *m/z*, calcd. for $C_{20}H_{21}N_3NaO_3$ [M + Na]$^+$: 374.15, found: 374.14.

*Data for 4-((4-allyl-2-methoxyphenoxy)methyl)-1-(4-iodophenyl)-1H-1,2,3-triazole (**2r**)* Compound **2r** was obtained as a white solid in 74% yield (0.245 g, 0.547 mmol) from 0.181 g (0.741 mmol) of 1-azido-4-iodobenzene, 0.150 g (0.741 mmol) of the alkyne **1**, 0.058 g (0.296 mmol) of sodium

ascorbate and 0.037 g (0.148 mmol) of $CuSO_4 \cdot 5H_2O$. Reaction time: 24 hours. TLC: R_f = 0.35 (hexane/ethyl acetate 4:1 v v^{-1}); mp 124.5—125.2 °C; IR (ATR) ν / cm^{-1} 3128, 3091, 2972, 2942, 2868, 2834, 1638, 1590, 1511, 1494, 1463, 1397, 1337, 1260, 1229, 1139, 1046, 1034, 1024, 985, 925, 819, 750, 698, 646, 605, 518, 468; ^{1}H NMR (600 MHz, DMSO-d_6) δ 3.30 (d, 2H, J = 7.2 Hz), 3.72 (s, 3H), 5.01—5.08 (m, 2H), 5.15 (s, 2H), 5.96 (ddt, 1H, J = 6.6, 9.6, 16.8 Hz), 6.68-6.70 (m, 1H), 6.80-6.81 (m, 1H), 7.06 (d, 1H, J = 8.4 Hz), 7.74 (d, 2H, J = 9.0 Hz), 7.96 (d, 2H, J = 9.0 Hz), 8.93 (s, 1H); ^{13}C NMR (150 MHz, DMSO-d_6) δ 39.5, 55.8, 62.2, 94.8, 113.0, 114.7, 116.0, 120.6, 122.4, 123.2, 133.6. 136.6, 138.3, 139.0, 144.6, 146.1, 149.5; LC-MS (ESI) *m/z*, calcd. for $C_{19}H_{18}IN_3NaO_2$ $[M + Na]^+$: 470.03, found: 470.15.

*Data for 4-((4-allyl-2-methoxyphenoxy)methyl)-1-(3-iodophenyl)-1H-1,2,3-triazole (**2s**)* Compound **2s** was obtained as a yellow solid in 65% yield (0.072 g, 0.161 mmol) from 0.060 g (0.247 mmol) of 1-azido-3-iodobenzene, 0.050 g (0.247 mmol) of the alkyne **1**, 0.019 g (0.098 mmol) of sodium ascorbate and 0.012 g (0.049 mmol) of $CuSO_4 \cdot 5H_2O$. Reaction time: 24 hours. TLC: R_f = 0.64 (hexane/ethyl acetate 3:2 v v^{-1}); mp 102.4—103.3 °C; IR (ATR) ν / cm^{-1} 3172, 3076, 3002, 2931, 2909, 2871, 2831, 1731, 1679, 1634, 1588, 1580, 1512, 1490, 1459, 1420, 1399, 1260, 1229, 1140, 1035, 1019, 989, 905, 831, 781, 709, 674, 645, 598, 546, 430; ^{1}H NMR (600 MHz, DMSO-d_6) δ 3.30 (d, 2H, J = 6.6 Hz), 3.74 (s, 3H), 5.01-5.08 (m, 2H), 5.15 (s, 2H), 5.96 (ddt, 1H, J = 7.2, 10.2, 17.4 Hz), 6.70 (dd, 1H, J = 1.8, 8.4 Hz), 6.80-6.81 (m, 1H), 7.06 (d, 1H, J = 7.8 Hz), 7.4 (d, 1H, J = 7.8 Hz), 7.86 (d, 1H, J = 7.8 Hz), 7.95-7.97 (m, 1H), 8.29-8.30 (m, 1H), 8.97 (s, 1H); ^{13}C NMR (150 MHz, DMSO-d_6) δ 39.5, 55.8, 62.2, 95.8, 113.0, 114.7, 116.0, 119.9, 120.6, 123.4, 128.6, 132.1, 133.6, 137.8, 137.9, 138.3, 144.6, 146.1, 149.6; LC-MS (ESI) *m/z*, calcd. for $C_{19}H_{18}IN_3NaO_2$ $[M + Na]^+$: 470.03, found: 470.12.

2. IR, NMR (^{1}H AND ^{13}C) AND LC-MS/MS SPECTRA OF COMPOUNDS 2a–2s

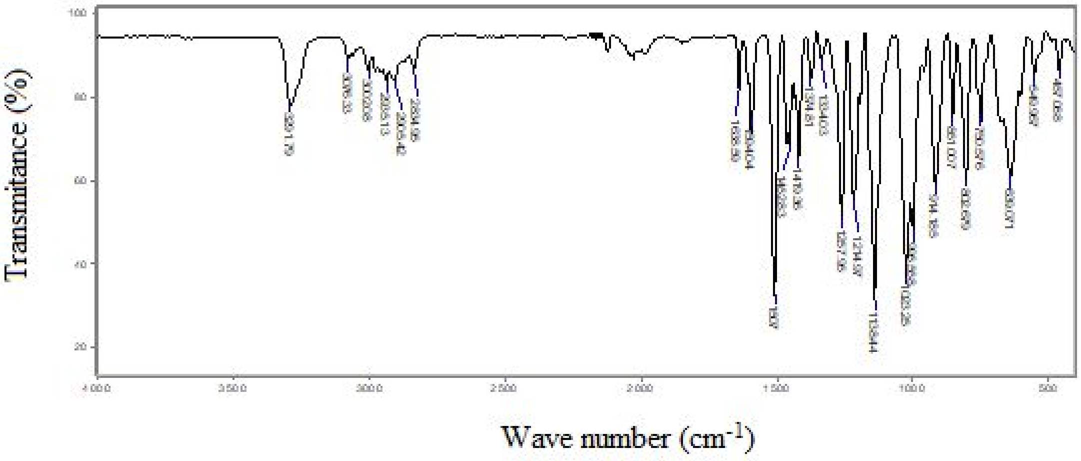

Figure S1. FTIR spectrum (ATR) of 4-allyl-2-methoxy-1-(prop-2-yn-1-yloxy)benzene **1**.

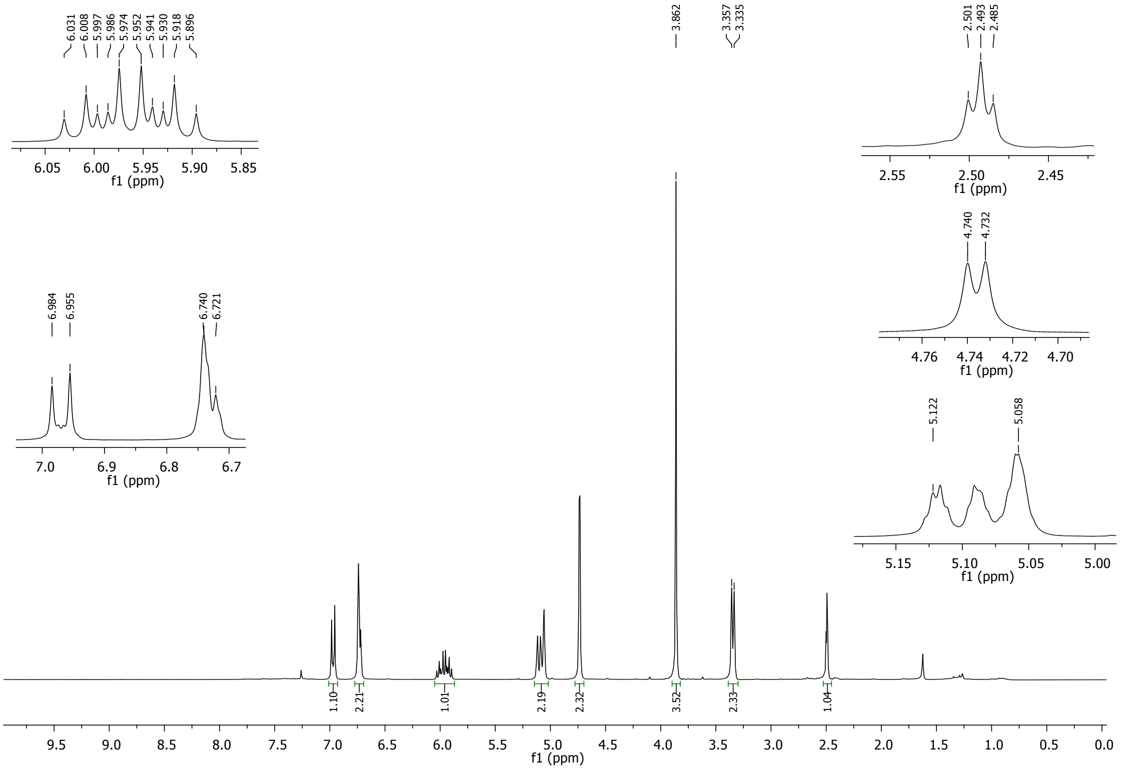

Figure S2. ^{1}H NMR spectrum (300 MHz, $CDCl_3$) of 4-allyl-2-methoxy-1-(prop-2-yn-1-yloxy)benzene (**1**).

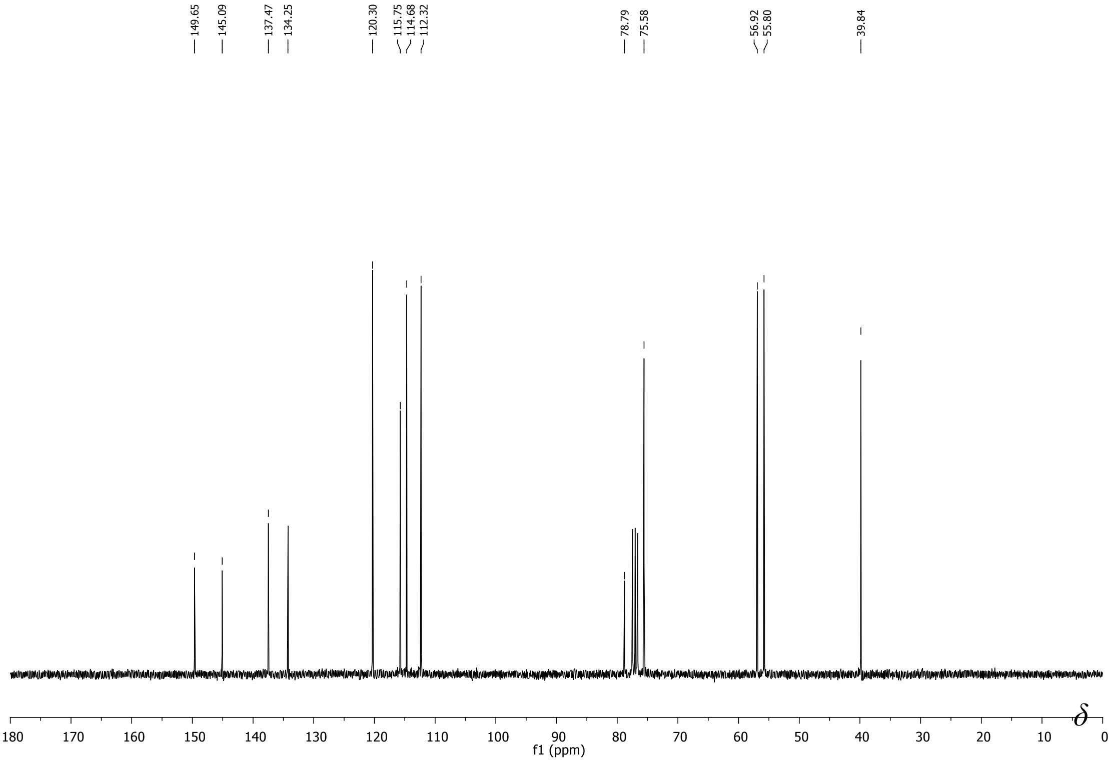

Figure S3. ^{13}C NMR spectrum (75 MHz, $CDCl_3$) of 4-allyl-2-methoxy-1-(prop-2-yn-1-yloxy)benzene (**1**).

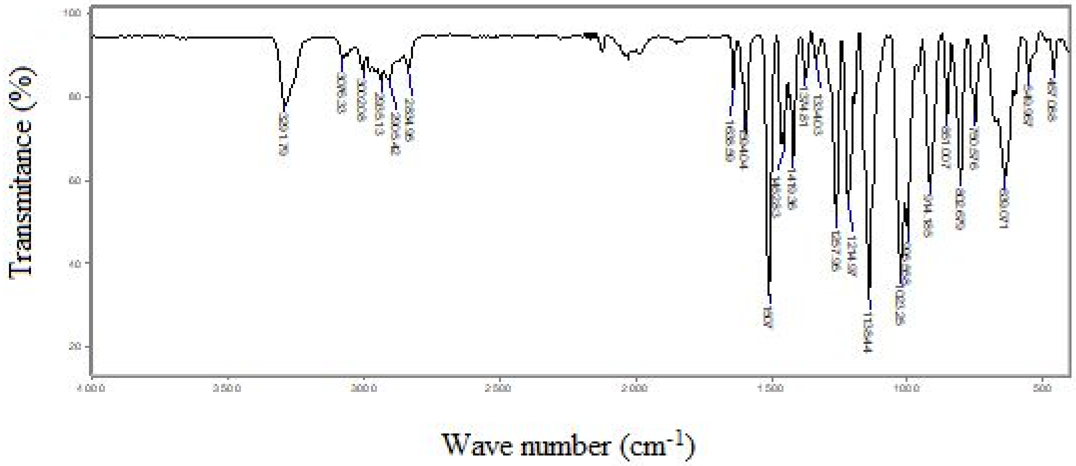

Figure S4. FTIR spectrum (ATR) of 4-((4-allyl-2-methoxyphenoxy)methyl)-1-(4-bromophenyl)-1*H*-1,2,3-triazole (**2a**).

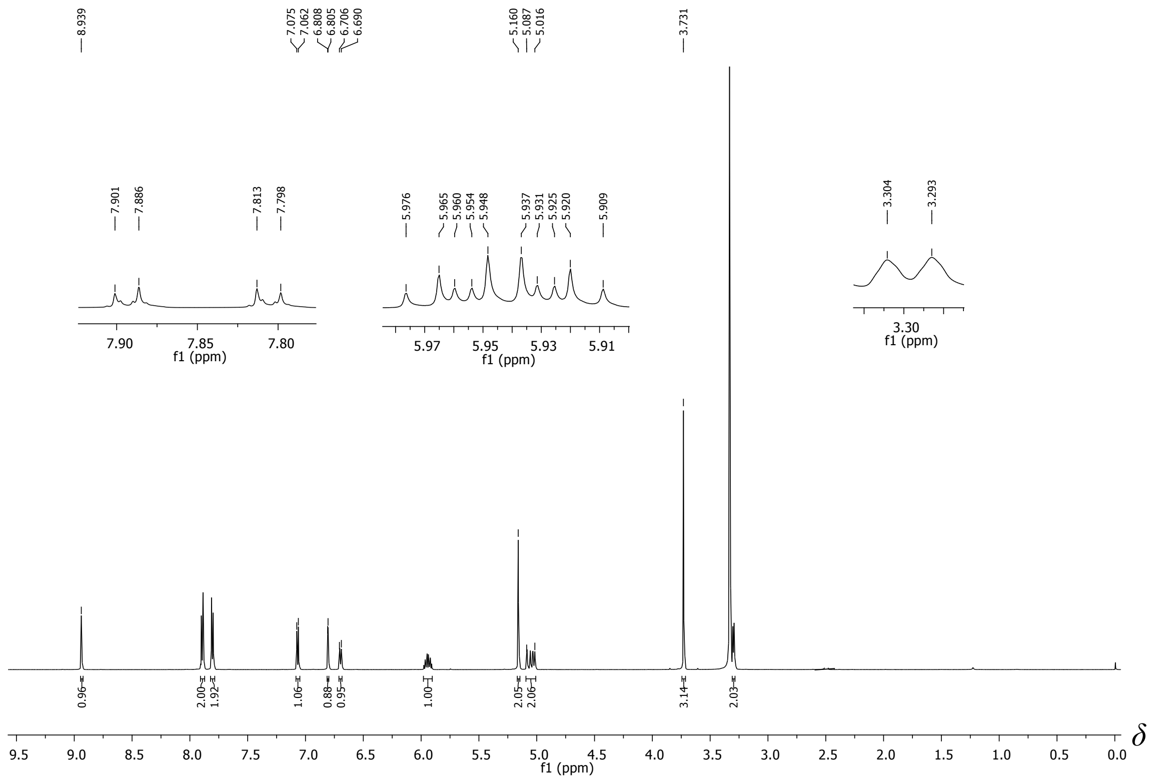

Figure S5. ^{1}H NMR spectrum (600 MHz, DMSO-d_6) of 4-((4-allyl-2-methoxyphenoxy)methyl)-1-(4-bromophenyl)-1*H*-1,2,3-triazole (**2a**).

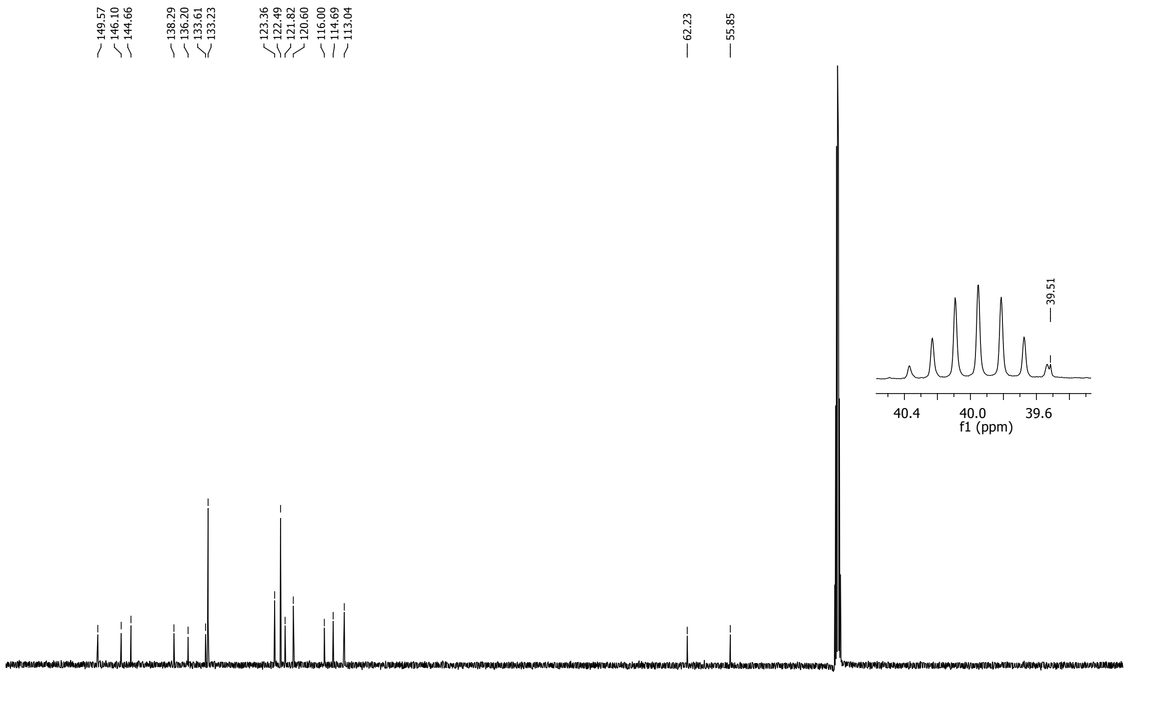

Figure S6. ^{13}C NMR spectrum (150 MHz, DMSO-d_6) of 4-((4-allyl-2-methoxyphenoxy)methyl)-1-(4-bromophenyl)-1*H*-1,2,3-triazole (**2a**).

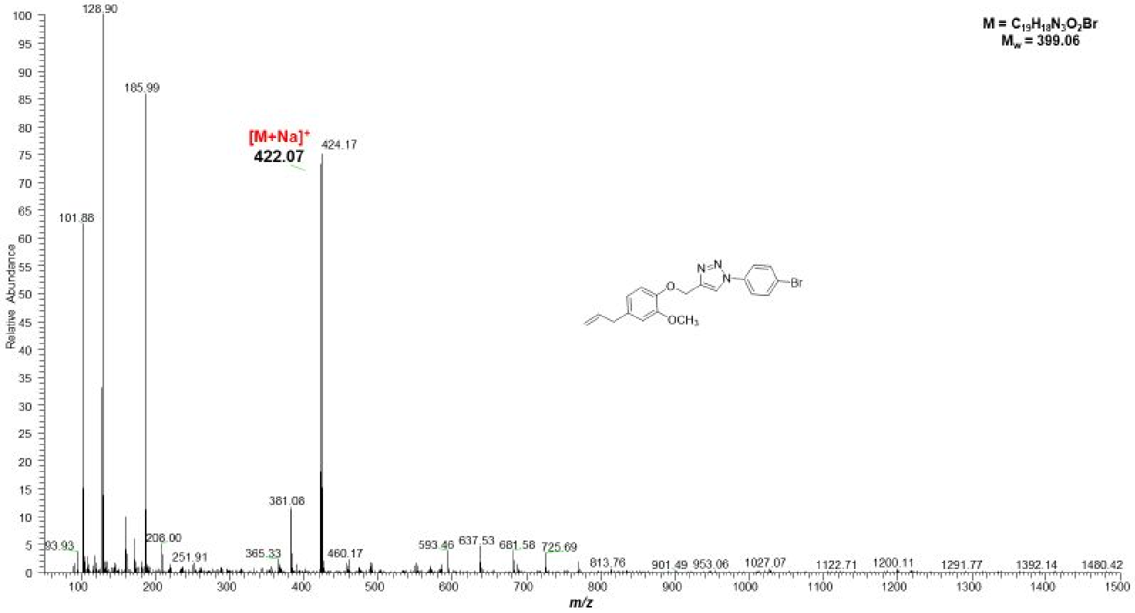

Figure S7. LC-MS spectrum of 4-((4-allyl-2-methoxyphenoxy)methyl)-1-(4-bromophenyl)-1*H*-1,2,3-triazole (**2a**).

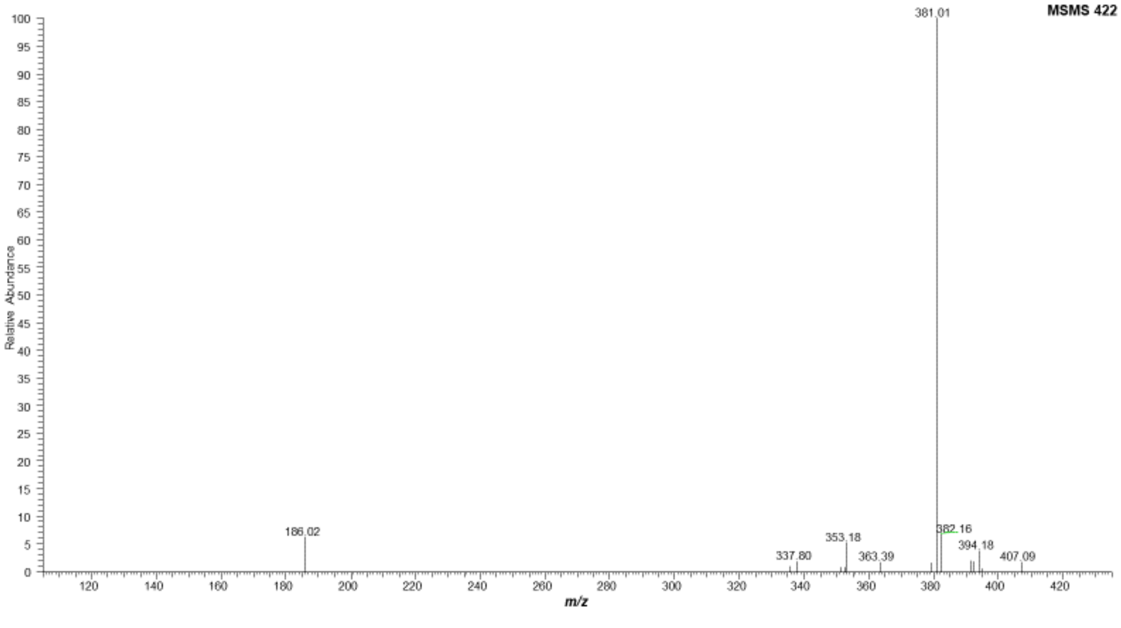

Figure S8. MS-MS spectrum of 4-((4-allyl-2-methoxyphenoxy)methyl)-1-(4-bromophenyl)-1*H*-1,2,3-triazole (**2a**).

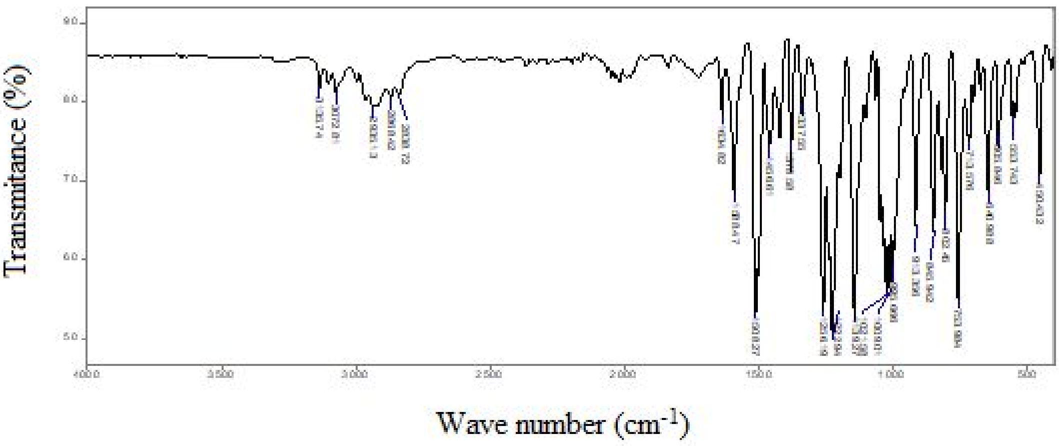

Figure S9. FTIR spectrum (ATR) of 4-((4-allyl-2-methoxyphenoxy)methyl)-1-(2-bromophenyl)-1*H*-1,2,3-triazole (**2b**).

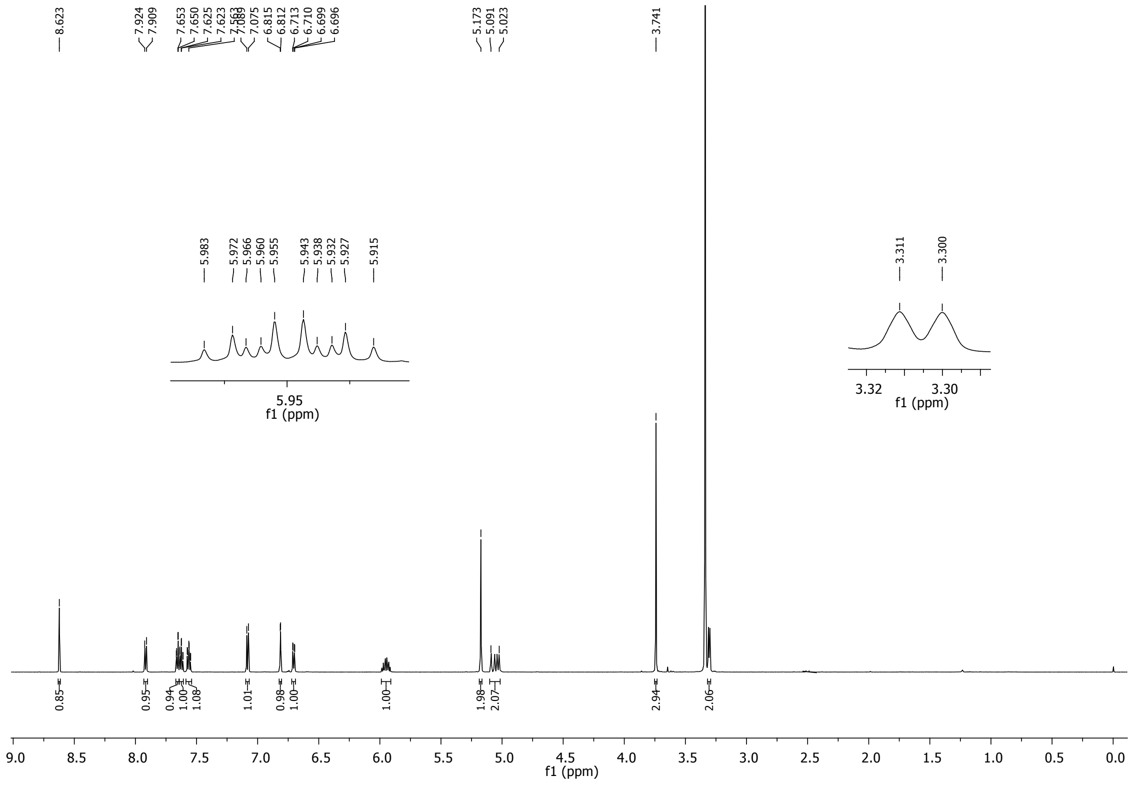

Figure S10. ^{1}H NMR spectrum (600 MHz, DMSO-d_6) of 4-((4-allyl-2-methoxyphenoxy)methyl)-1-(2-bromophenyl)-1*H*-1,2,3-triazole (**2b**).

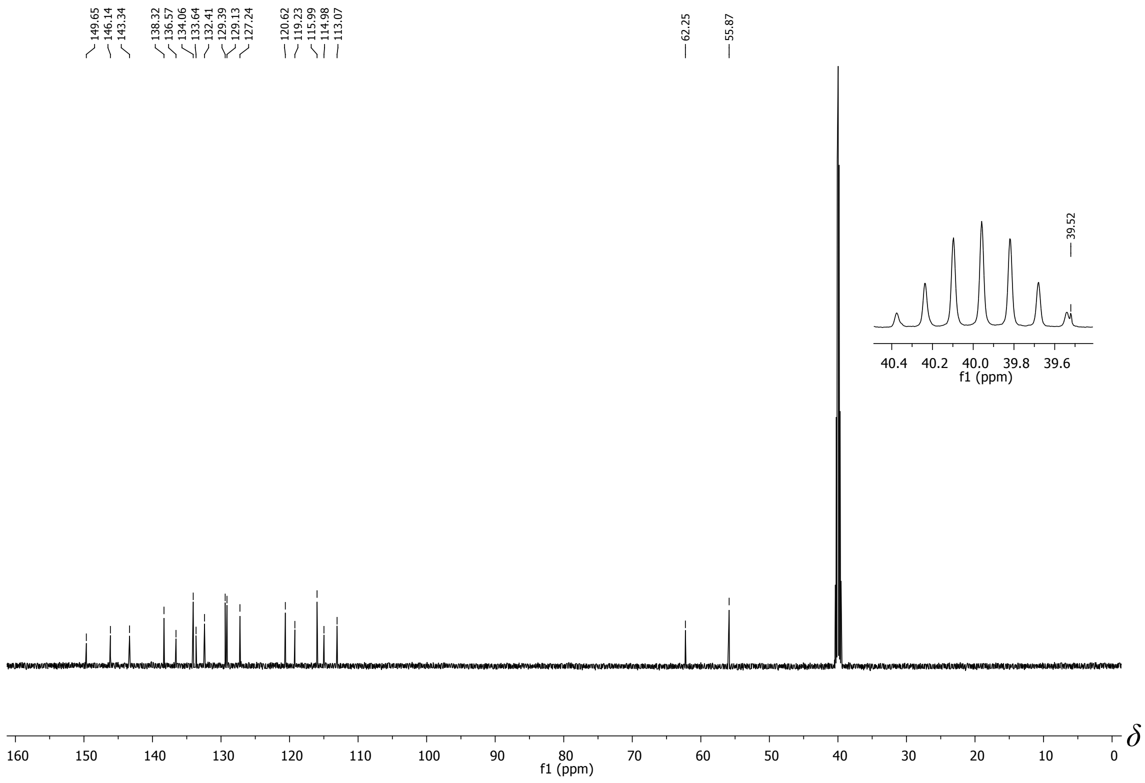

Figure S11. ^{13}C NMR spectrum (150 MHz, DMSO-d_6) of 4-((4-allyl-2-methoxyphenoxy)methyl)-1-(2-bromophenyl)-1*H*-1,2,3-triazole (**2b**).

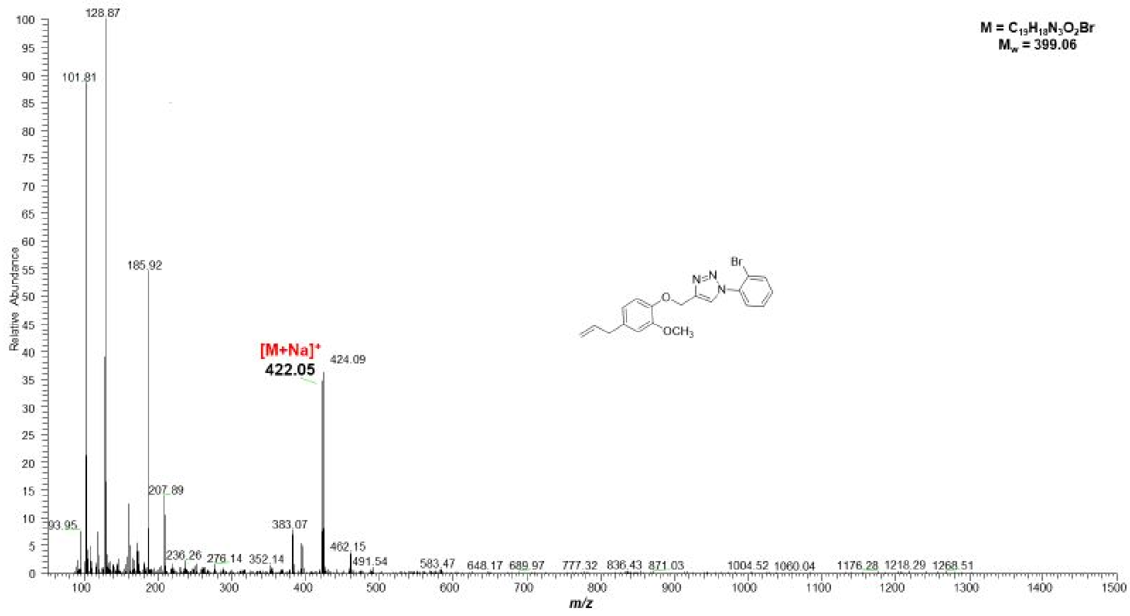

Figure S12. LC-MS spectrum of 4-((4-allyl-2-methoxyphenoxy)methyl)-1-(2-bromophenyl)-1*H*-1,2,3-triazole (**2b**).

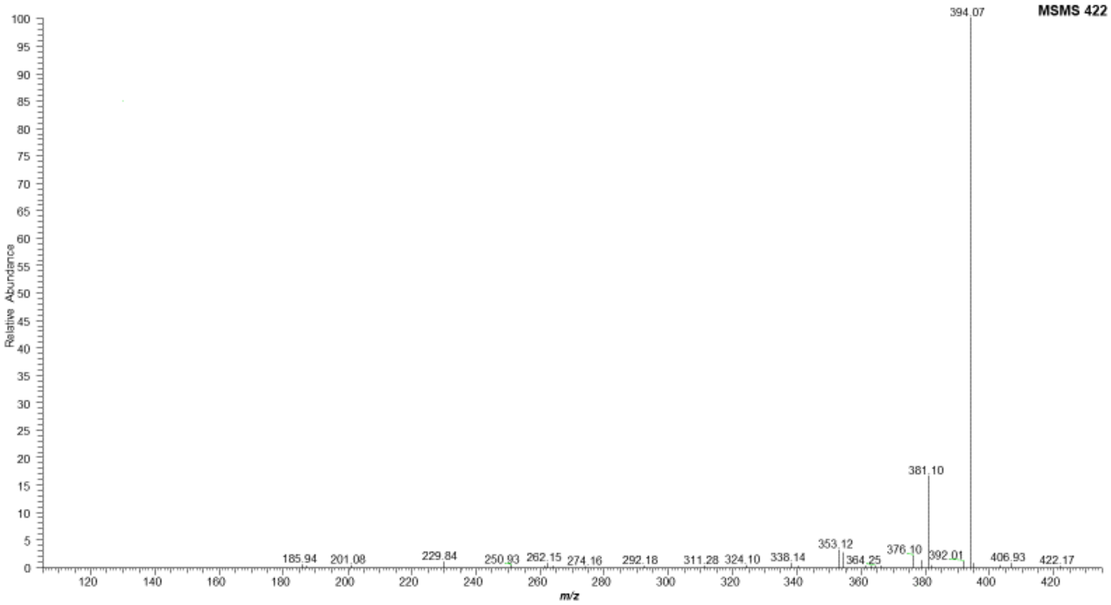

Figure S13. MS-MS spectrum of 4-((4-allyl-2-methoxyphenoxy)methyl)-1-(2-bromophenyl)-1*H*-1,2,3-triazole (**2b**).

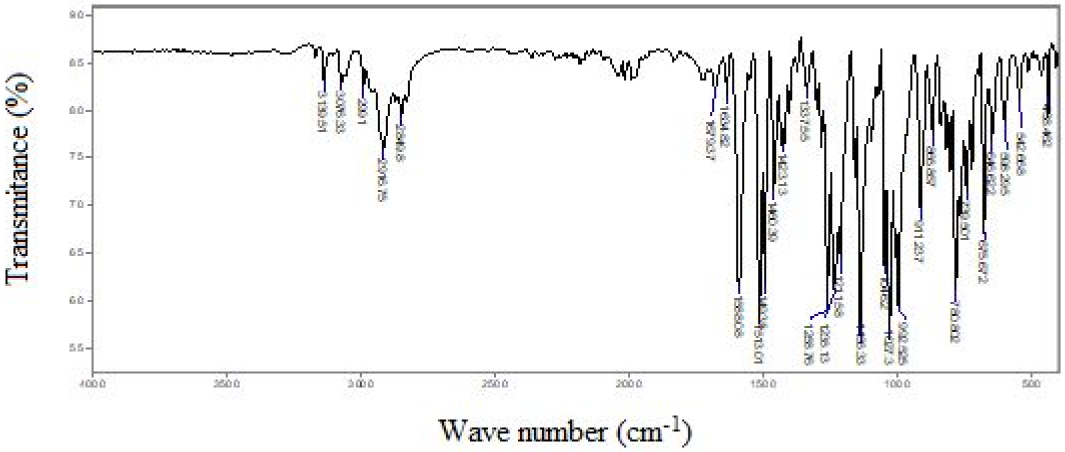

Figure S14. FTIR spectrum (ATR) of 4-((4-allyl-2-methoxyphenoxy)methyl)-1-(3-bromophenyl)-1*H*-1,2,3-triazole (**2c**).

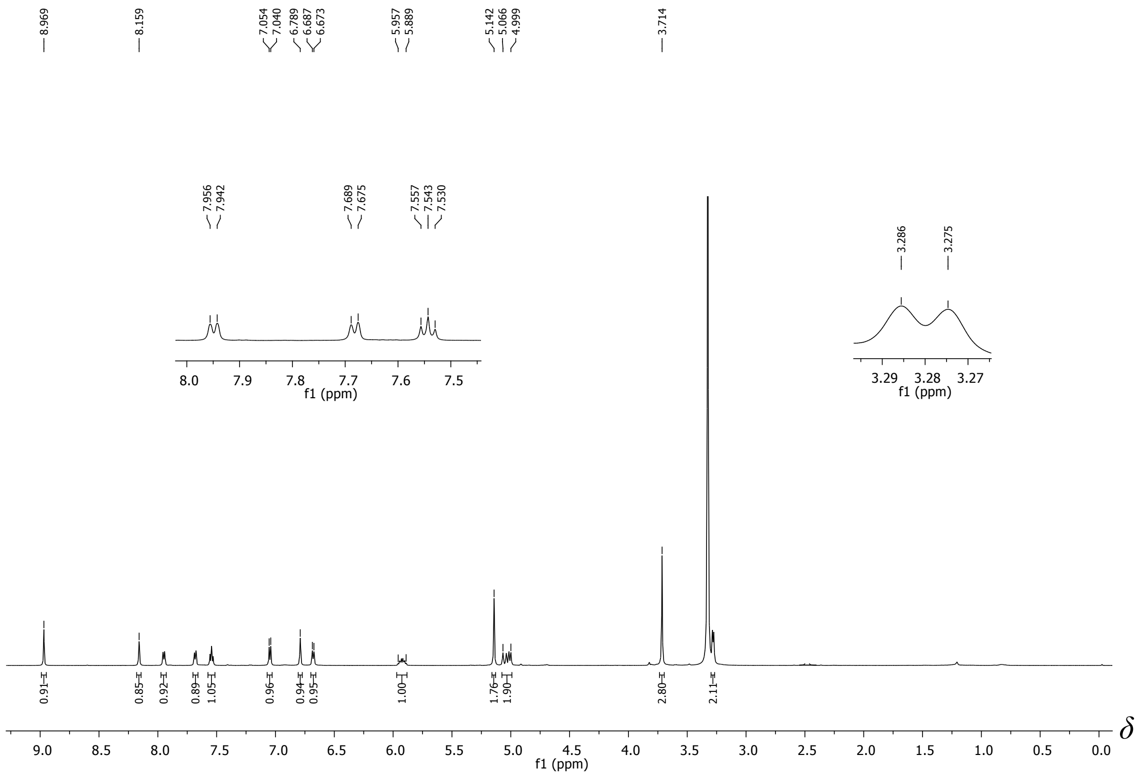

Figure S15. ^{1}H NMR spectrum (600 MHz, DMSO-d_6) of 4-((4-allyl-2-methoxyphenoxy)methyl)-1-(3-bromophenyl)-1*H*-1,2,3-triazole (**2c**).

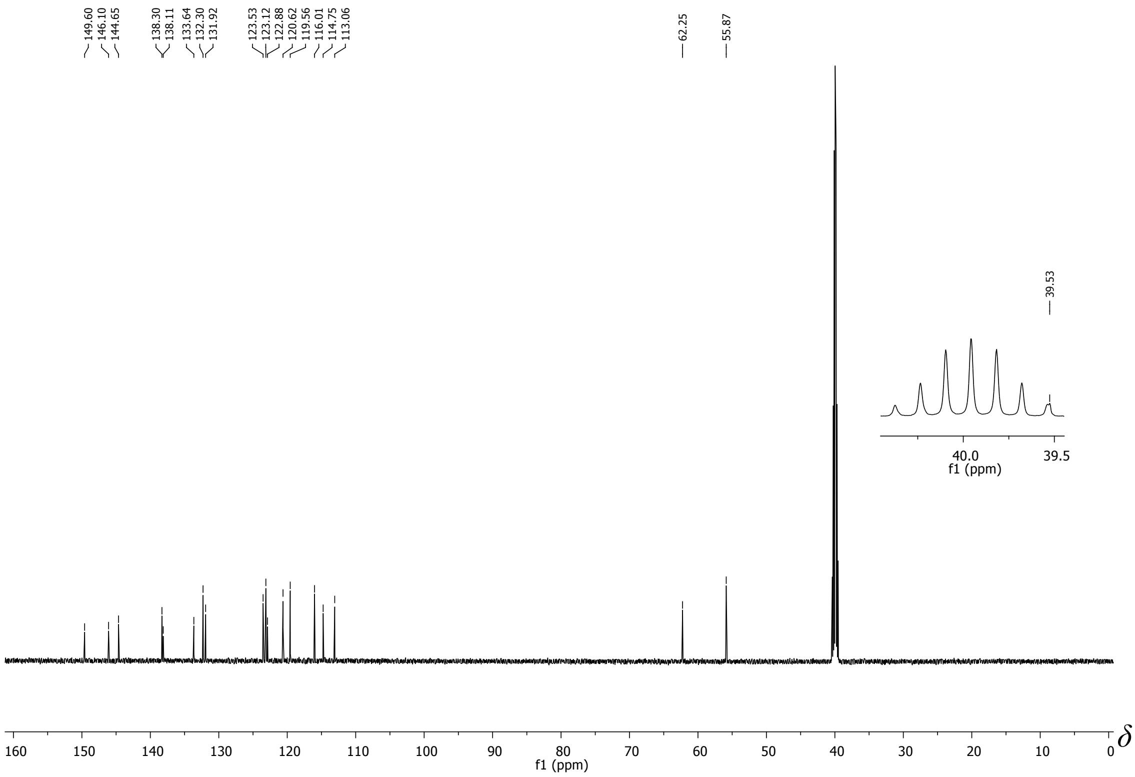

Figure S16. ^{13}C NMR spectrum (150 MHz, DMSO-d_6) of 4-((4-allyl-2-methoxyphenoxy)methyl)-1-(3-bromophenyl)-1*H*-1,2,3-triazole (**2c**).

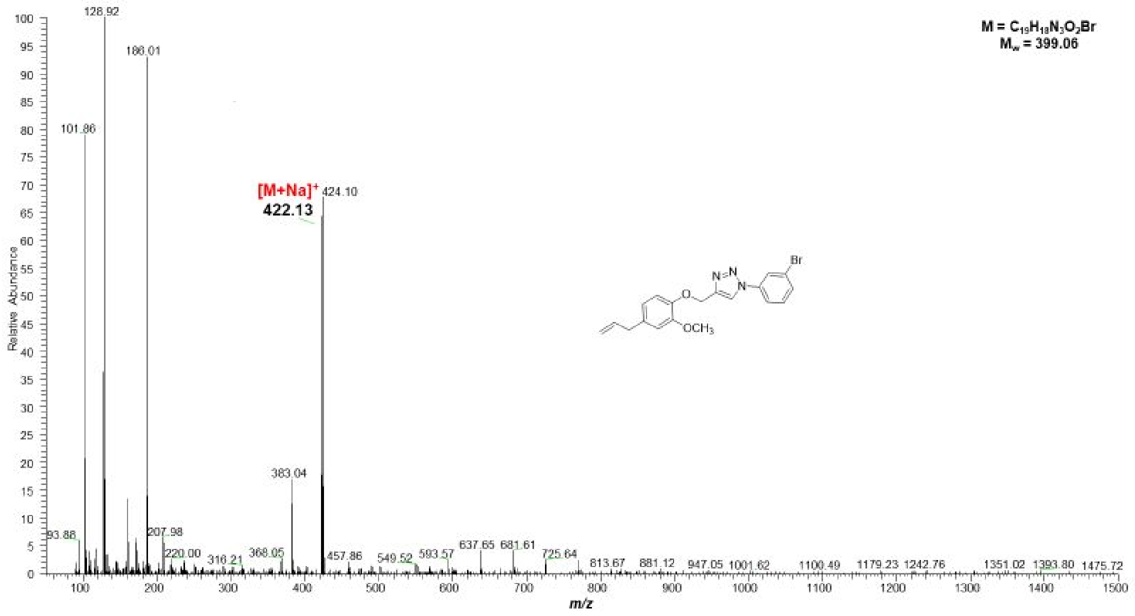

Figure S17. LC-MS spectrum of 4-((4-allyl-2-methoxyphenoxy)methyl)-1-(3-bromophenyl)-1*H*-1,2,3-triazole (**2c**).

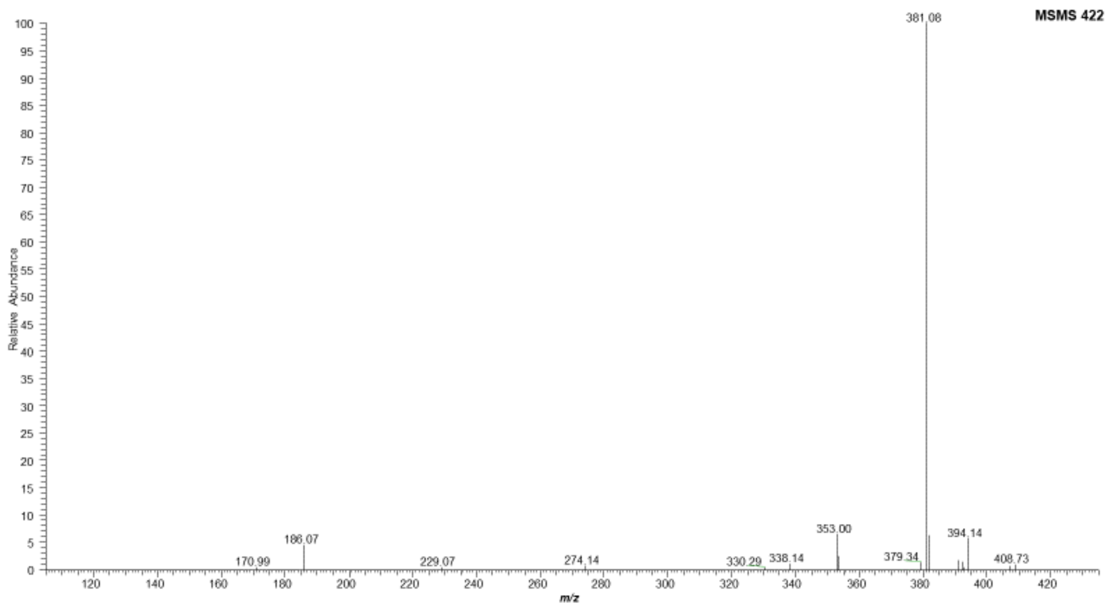

Figure S18. MS-MS spectrum of 4-((4-allyl-2-methoxyphenoxy)methyl)-1-(3-bromophenyl)-1*H*-1,2,3-triazole (**2c**).

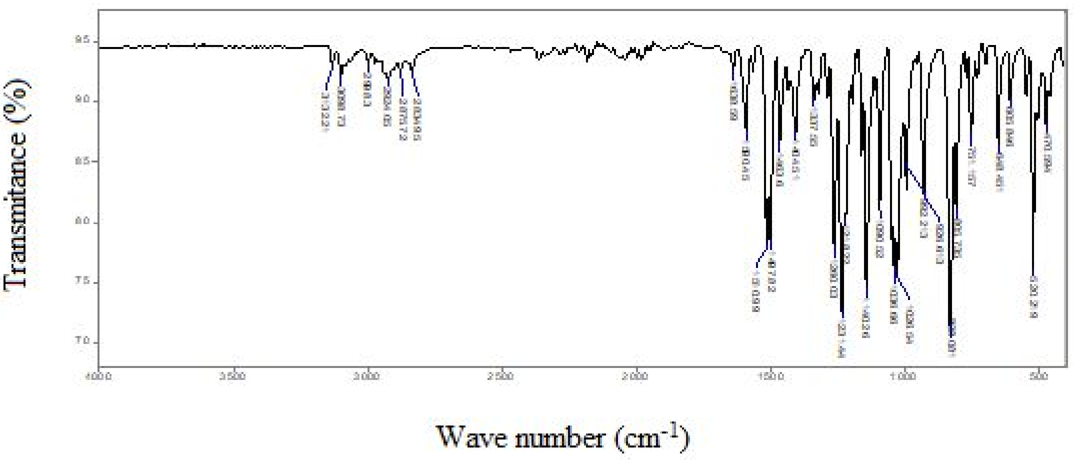

Figure S19. FTIR spectrum (ATR) of 4-((4-allyl-2-methoxyphenoxy)methyl)-1-(4-chlorophenyl)-1*H*-1,2,3-triazole (**2d**).

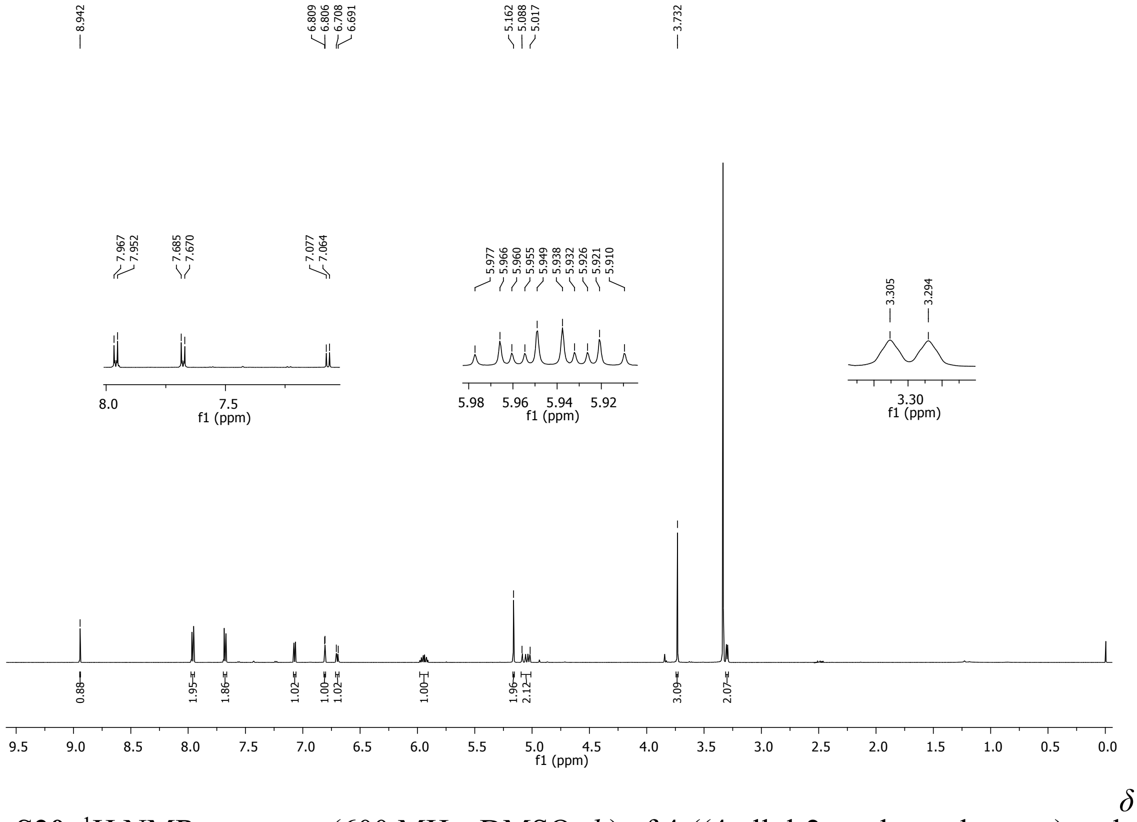

Figure S20. ^{1}H NMR spectrum (600 MHz, DMSO-d_6) of 4-((4-allyl-2-methoxyphenoxy)methyl)-1-(4-chlorophenyl)-1*H*-1,2,3-triazole (**2d**).

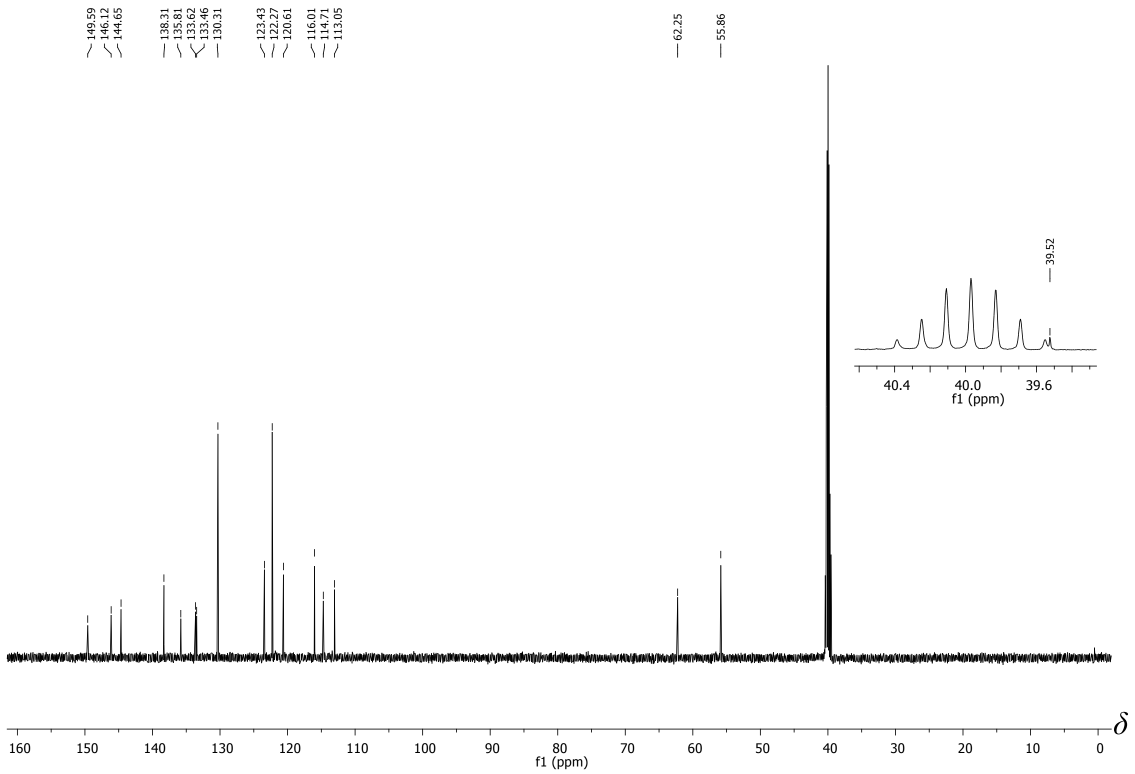

Figure S21. ^{13}C NMR spectrum (150 MHz, DMSO-d_6) of 4-((4-allyl-2-methoxyphenoxy)methyl)-1-(4-chlorophenyl)-1*H*-1,2,3-triazole (**2d**).

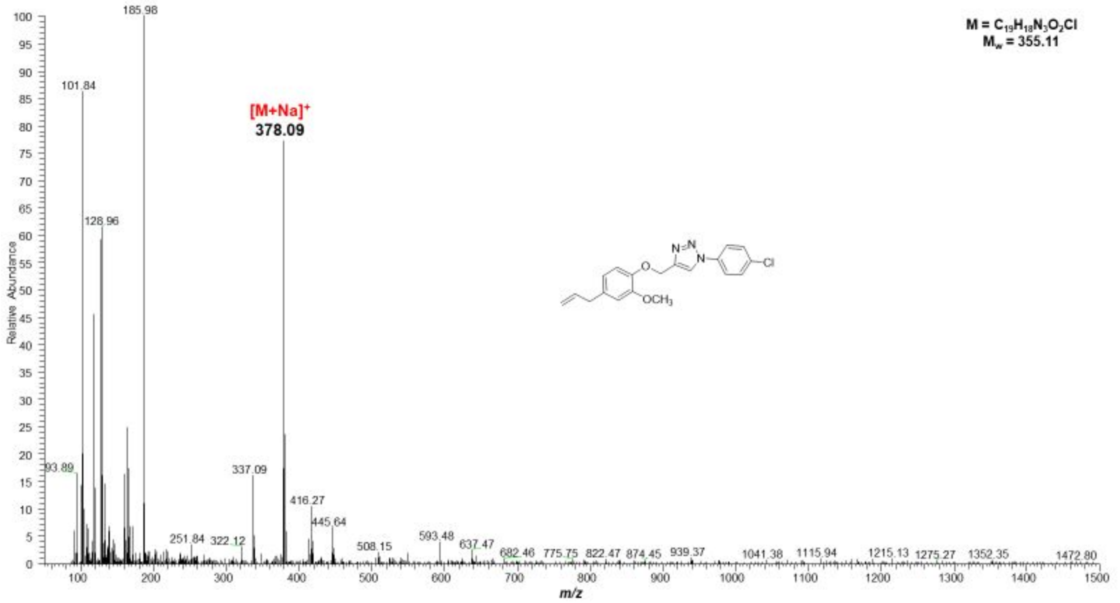

Figure S22. LC-MS spectrum of 4-((4-allyl-2-methoxyphenoxy)methyl)-1-(4-chlorophenyl)-1*H*-1,2,3-triazole (**2d**).

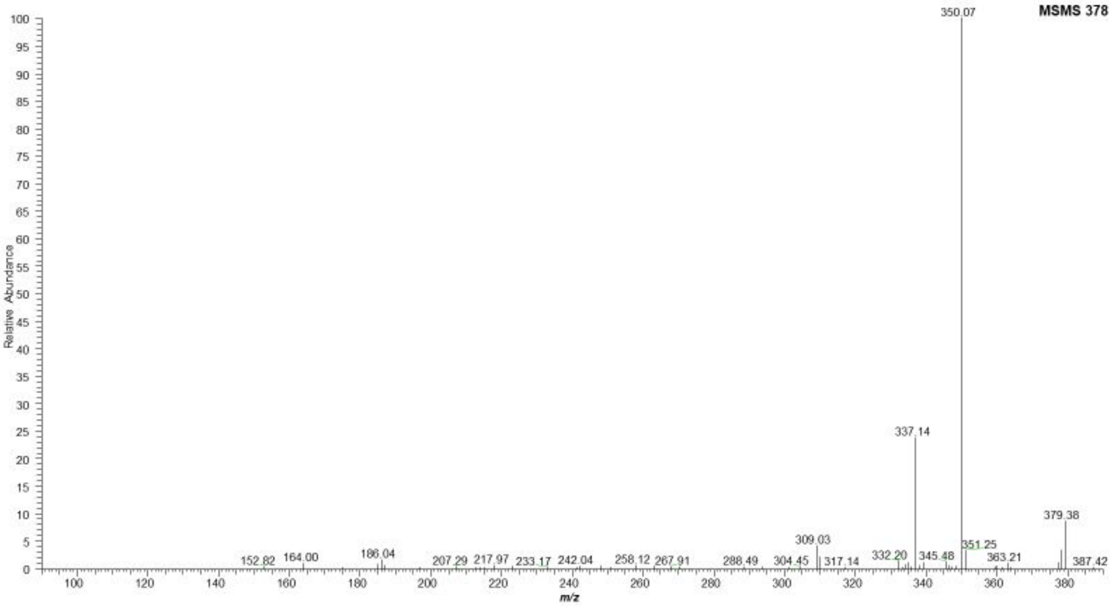

Figure S23. MS-MS spectrum of 4-((4-allyl-2-methoxyphenoxy)methyl)-1-(4-chlorophenyl)-1*H*-1,2,3-triazole (**2d**).

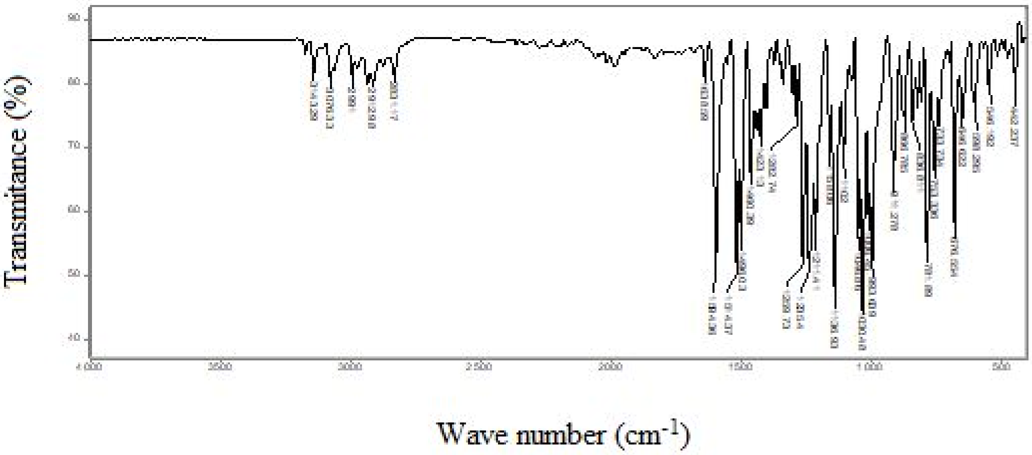

Figure S24. FTIR spectrum (ATR) of 4-((4-allyl-2-methoxyphenoxy)methyl)-1-(3-chlorophenyl)-1*H*-1,2,3-triazole (**2e**).

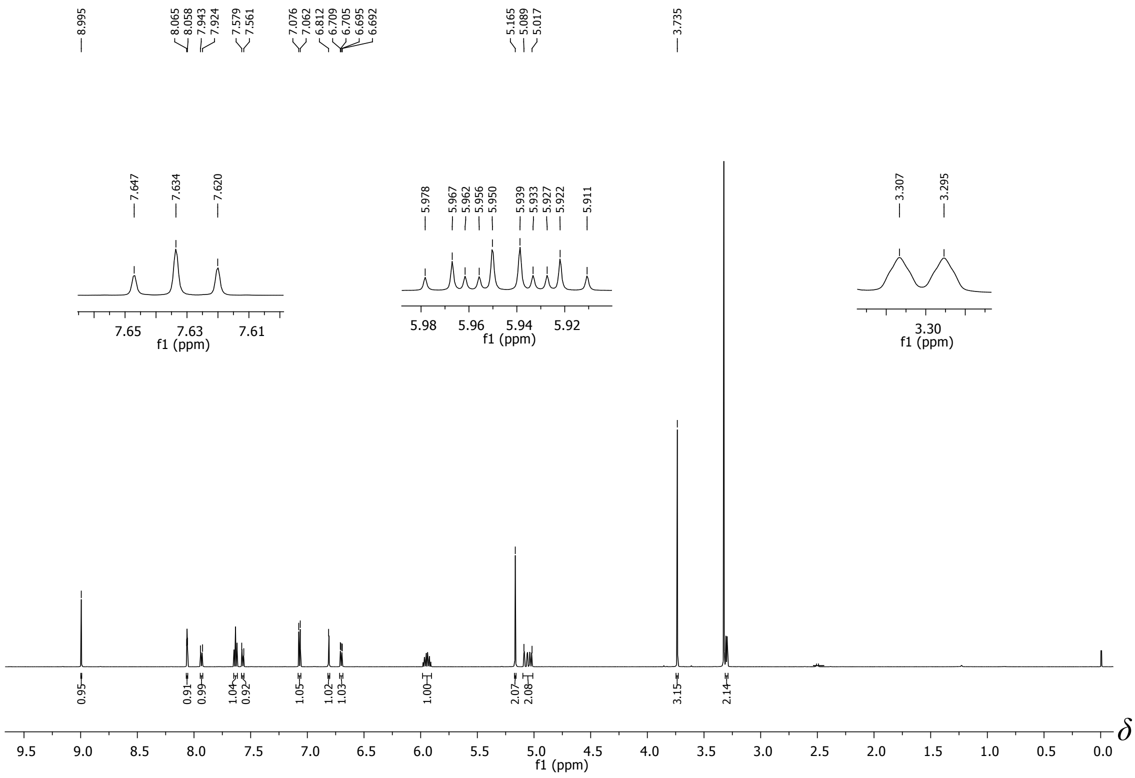

Figure S25. ^{1}H NMR spectrum (600 MHz, DMSO-d_6) of 4-((4-allyl-2-methoxyphenoxy)methyl)-1-(3-chlorophenyl)-1*H*-1,2,3-triazole (**2e**).

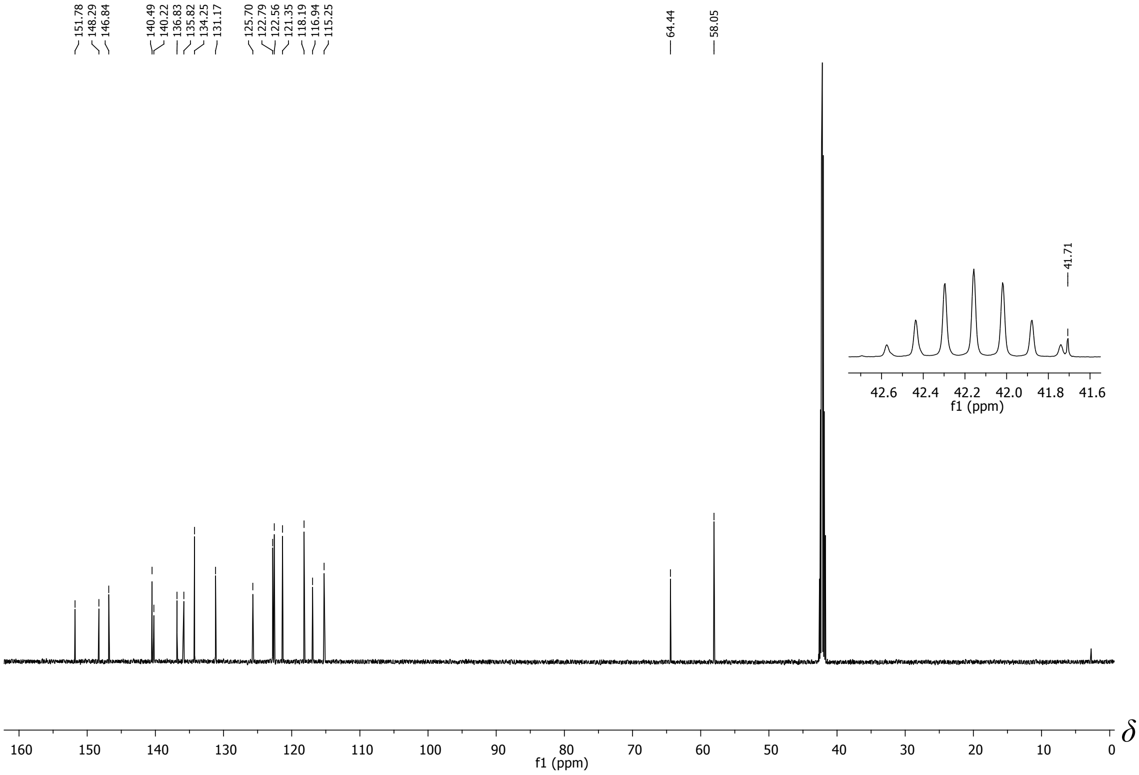

Figure S26. ^{13}C NMR spectrum (150 MHz, DMSO-d_6) of 4-((4-allyl-2-methoxyphenoxy)methyl)-1-(3-chlorophenyl)-1*H*-1,2,3-triazole (**2e**).

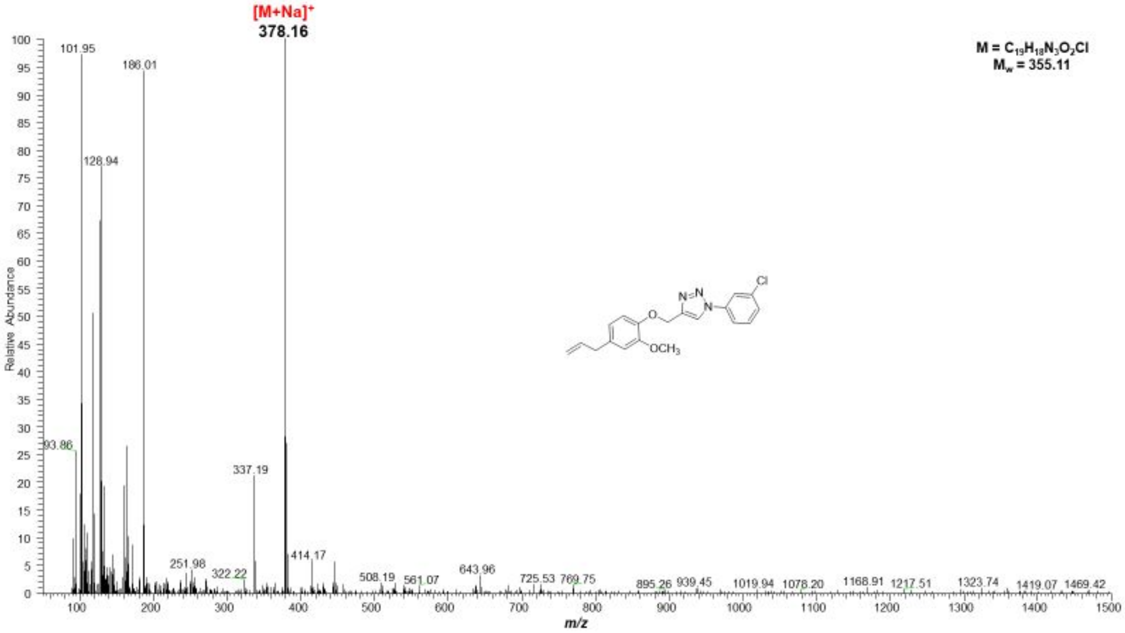

Figure S27. LC-MS spectrum of 4-((4-allyl-2-methoxyphenoxy)methyl)-1-(3-chlorophenyl)-1*H*-1,2,3-triazole (**2e**).

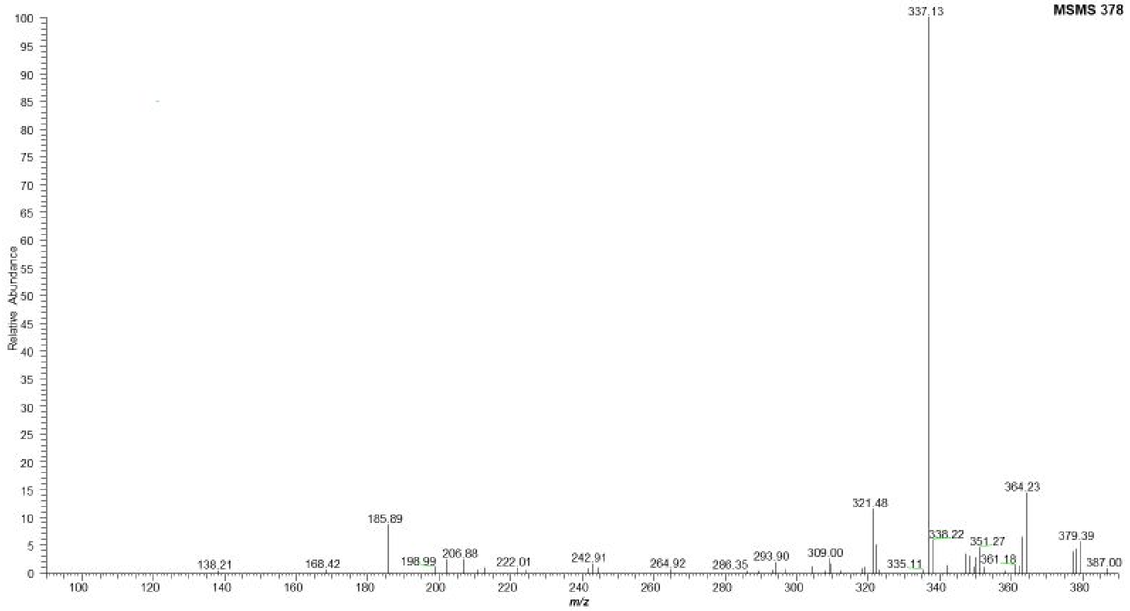

Figure S28. MS-MS spectrum of 4-((4-allyl-2-methoxyphenoxy)methyl)-1-(3-chlorophenyl)-1*H*-1,2,3-triazole (**2e**).

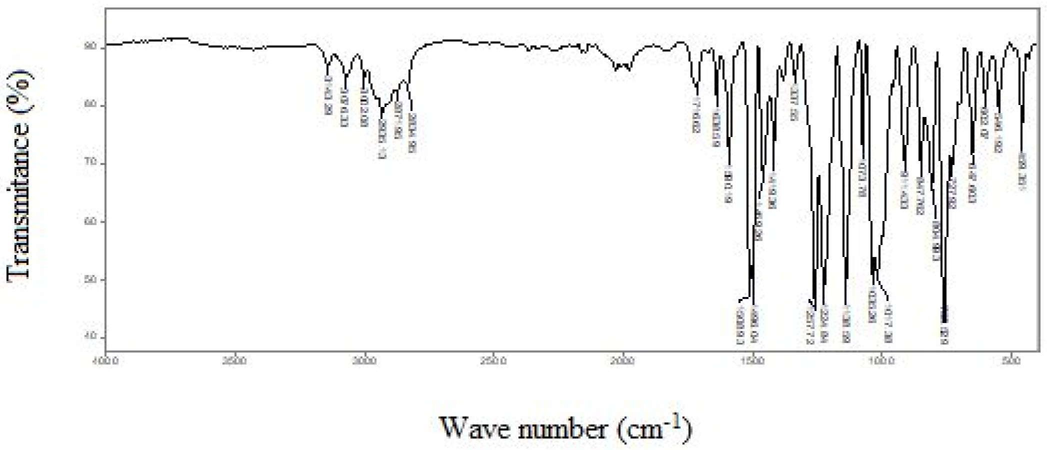

Figure S29. FTIR spectrum (ATR) of 4-((4-allyl-2-methoxyphenoxy)methyl)-1-(2-chlorophenyl)-1*H*-1,2,3-triazole (**2f**).

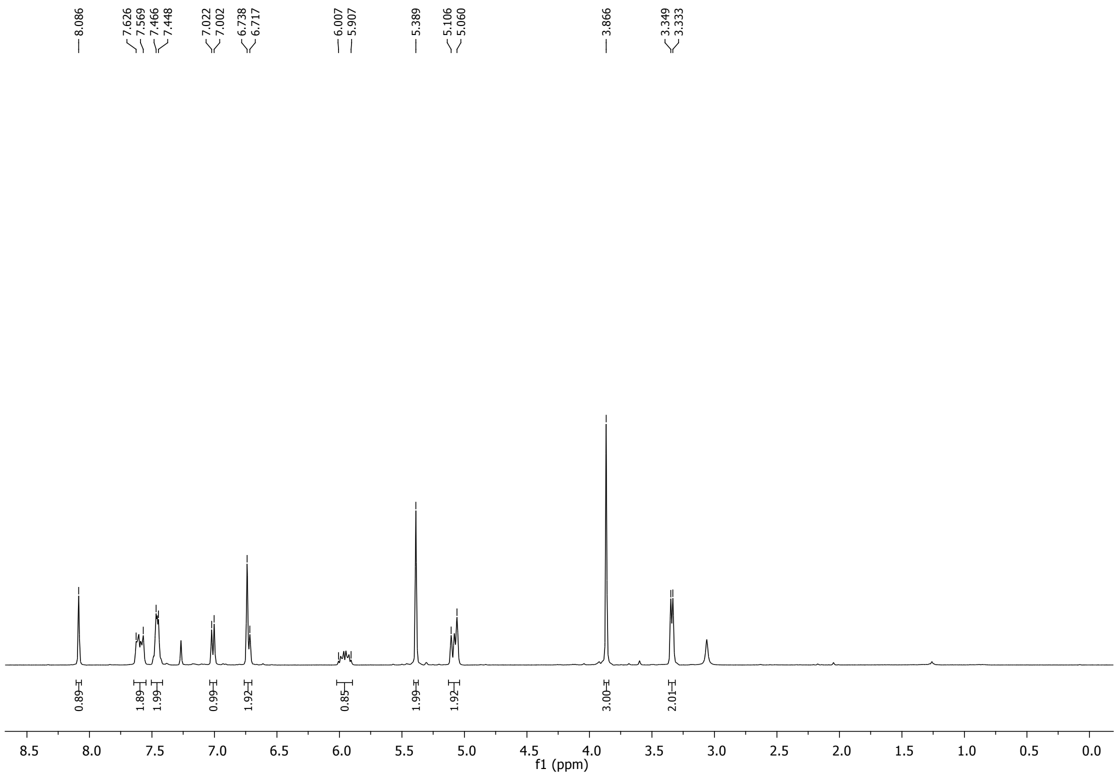

Figure S30. ^{1}H NMR spectrum (400 MHz, $CDCl_3$) of 4-((4-allyl-2-methoxyphenoxy)methyl)-1-(2-chlorophenyl)-1*H*-1,2,3-triazole (**2f**).

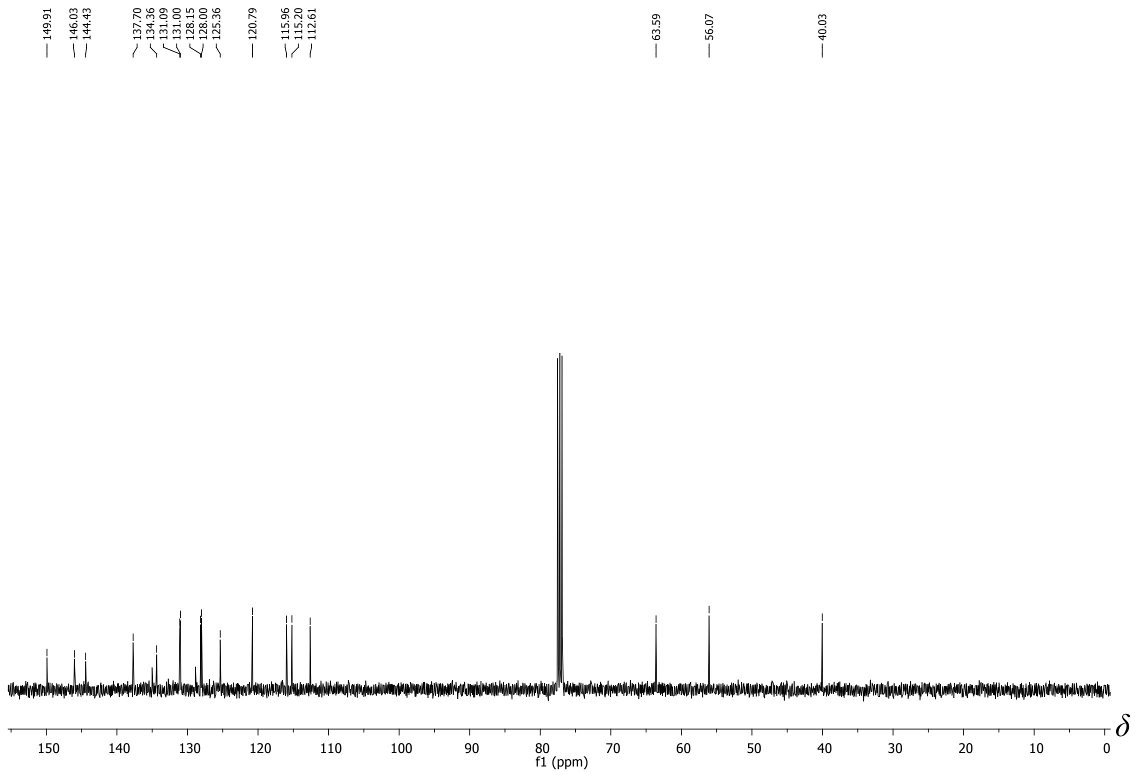

Figure S31. ^{13}C NMR spectrum (100 MHz, $CDCl_3$) of 4-((4-allyl-2-methoxyphenoxy)methyl)-1-(2-chlorophenyl)-1*H*-1,2,3-triazole (**2f**).

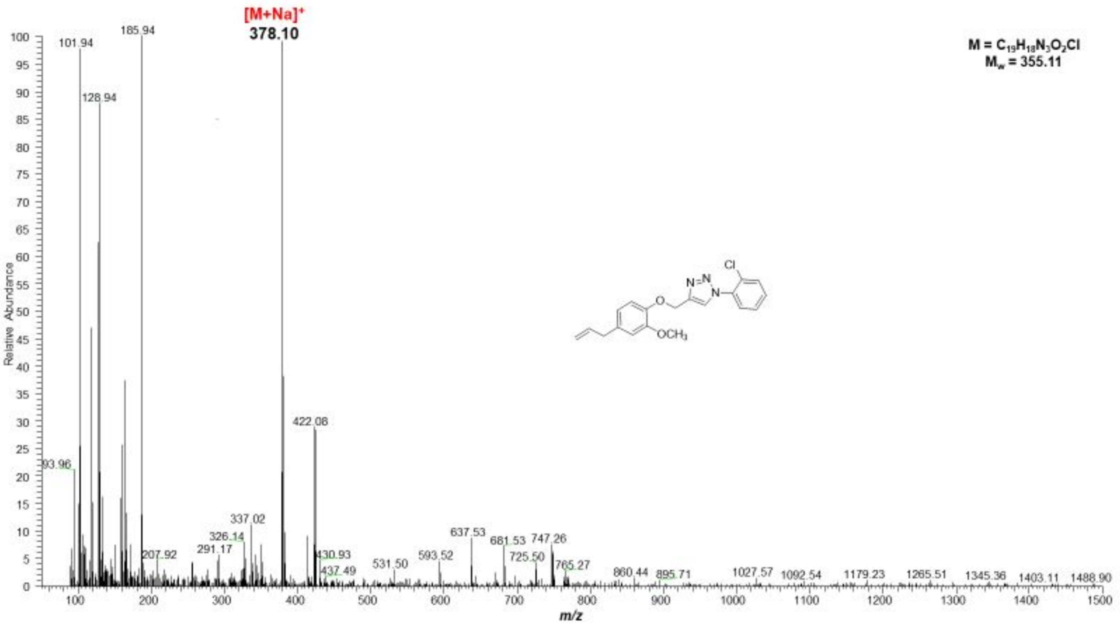

Figure S32. LC-MS spectrum of 4-((4-allyl-2-methoxyphenoxy)methyl)-1-(2-chlorophenyl)-1*H*-1,2,3-triazole (**2f**).

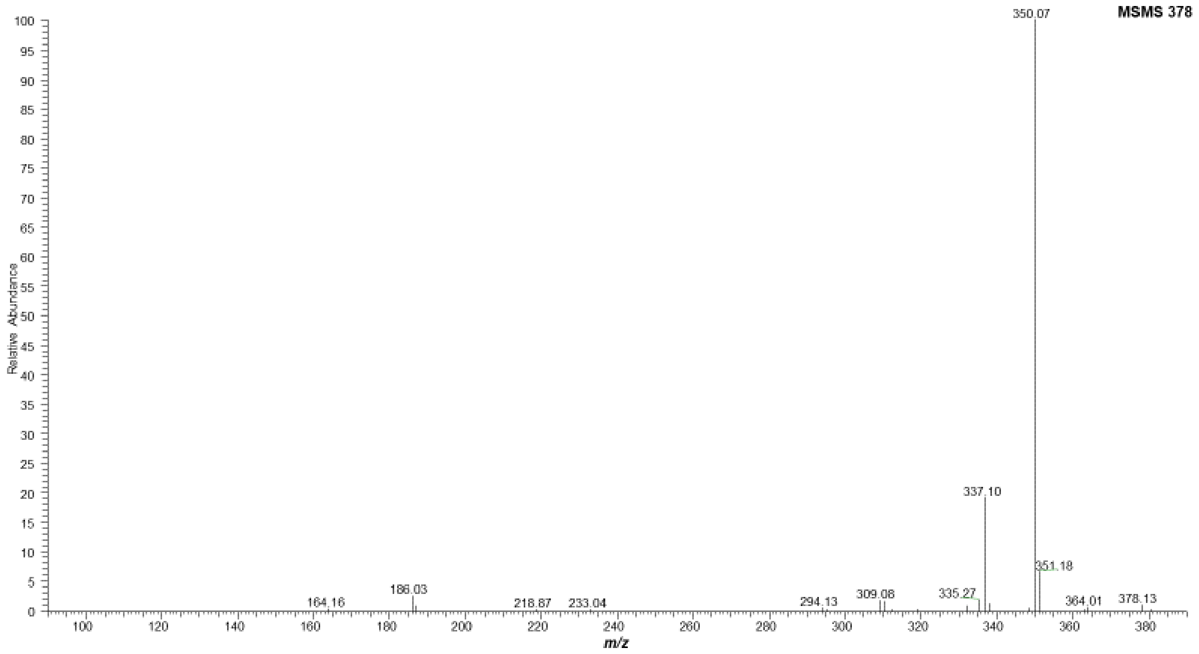

Figure S33. MS-MS spectrum of 4-((4-allyl-2-methoxyphenoxy)methyl)-1-(2-chlorophenyl)-1*H*-1,2,3-triazole (**2f**).

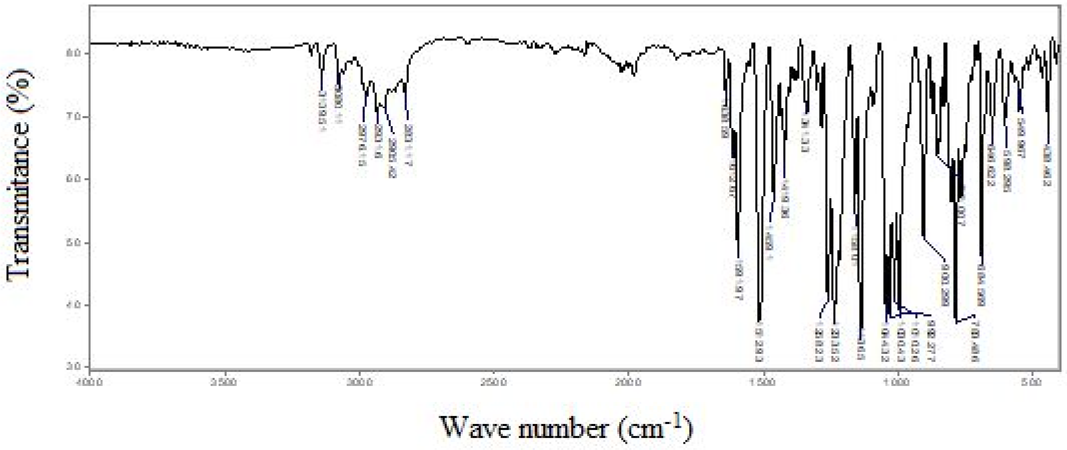

Figure S34. FTIR spectrum (ATR) of 4-((4-allyl-2-methoxyphenoxy)methyl)-1-(*m*-tolyl)-1*H*-1,2,3-triazole (**2g**).

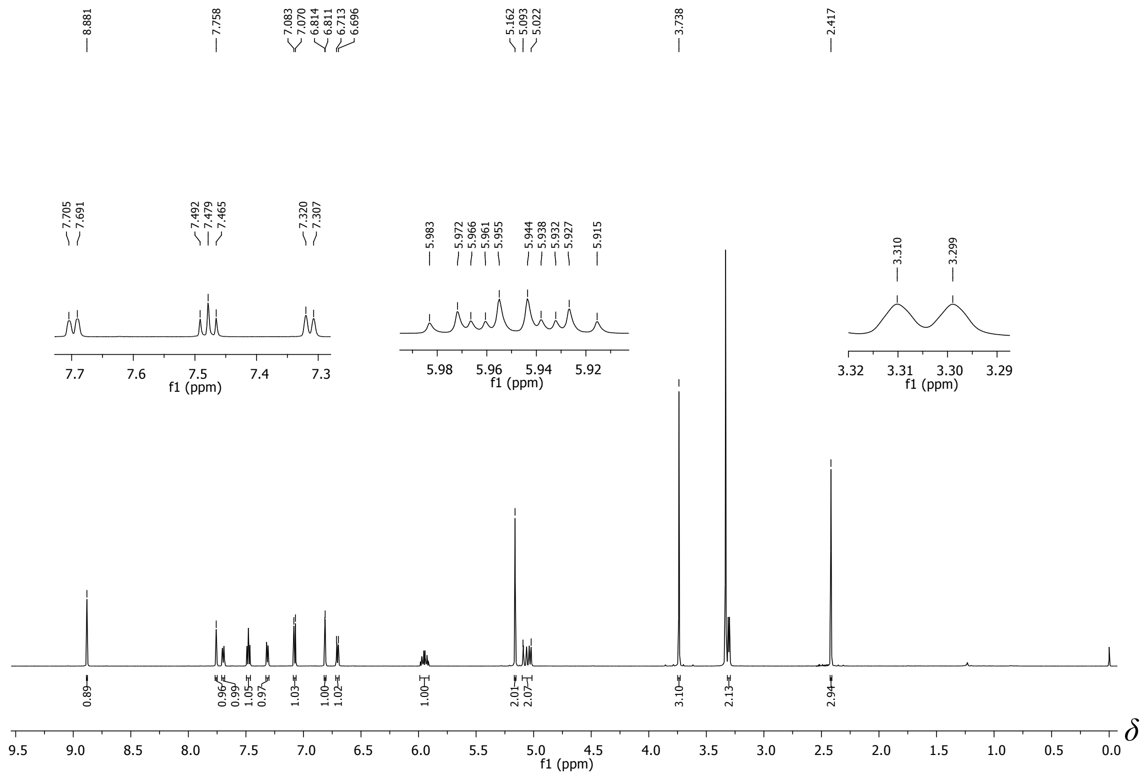

Figure S35. ^{1}H NMR spectrum (600 MHz, DMSO-d_6) of 4-((4-allyl-2-methoxyphenoxy)methyl)-1-(*m*-tolyl)-1*H*-1,2,3-triazole (**2g**).

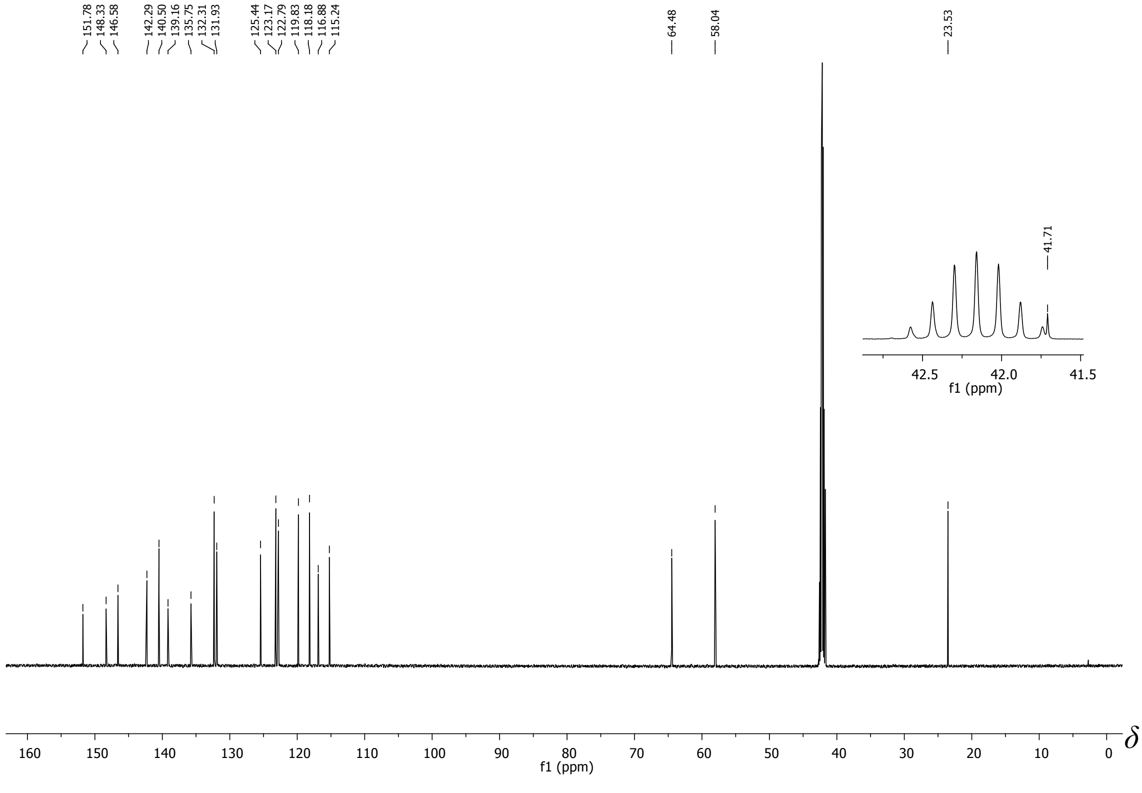

Figure S36. ^{13}C NMR spectrum (150 MHz, DMSO-d_6) of 4-((4-allyl-2-methoxyphenoxy)methyl)-1-(*m*-tolyl)-1*H*-1,2,3-triazole (**2g**).

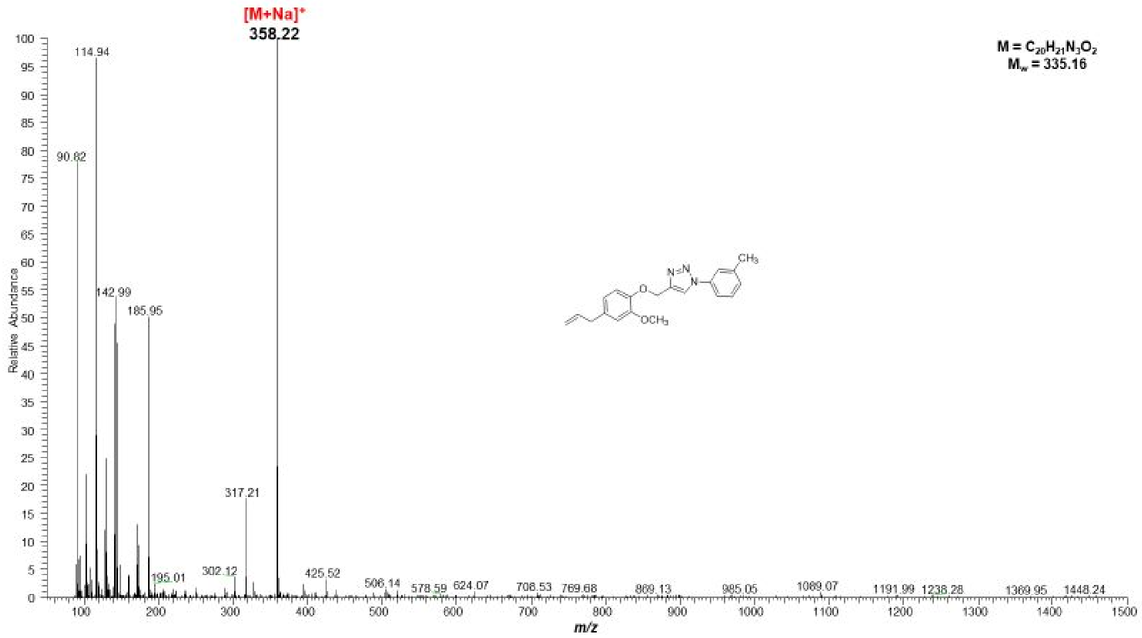

Figure S37. LC-MS spectrum of 4-((4-allyl-2-methoxyphenoxy)methyl)-1-(*m*-tolyl)-1*H*-1,2,3-triazole (**2g**).

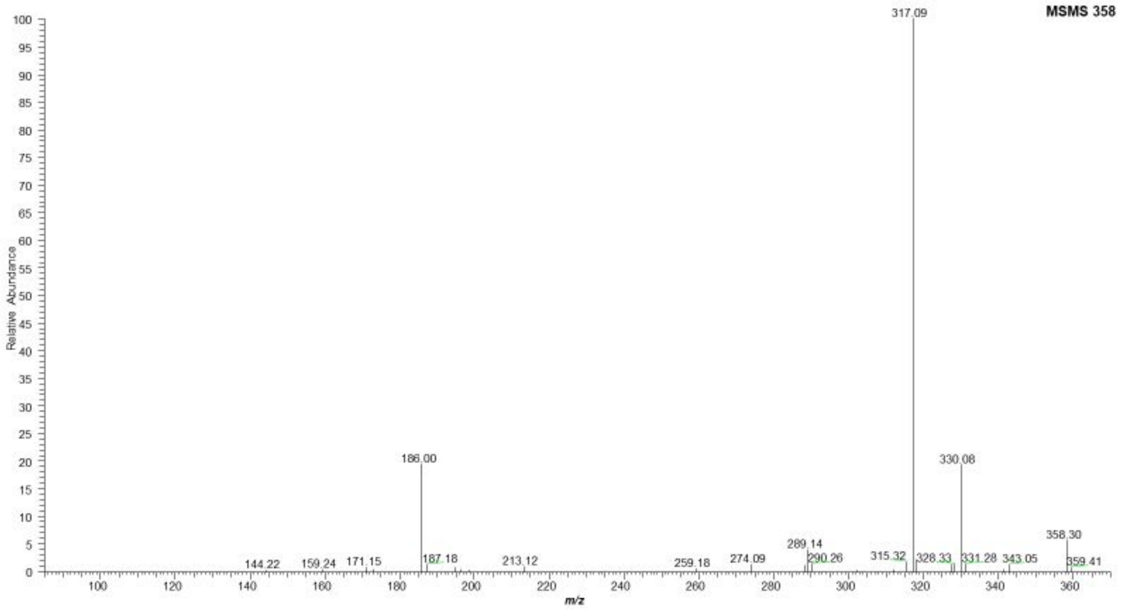

Figure S38. MS-MS spectrum of 4-((4-allyl-2-methoxyphenoxy)methyl)-1-(*m*-tolyl)-1*H*-1,2,3-triazole (**2g**).

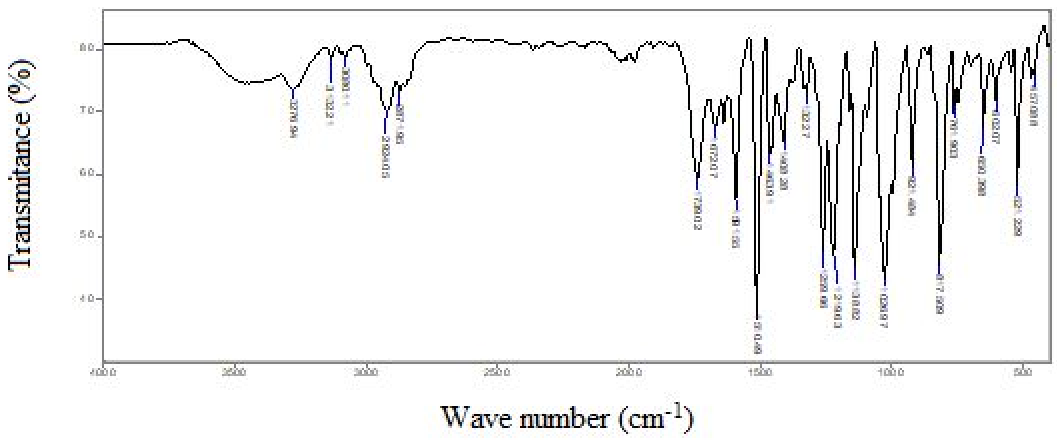

Figure S39. FTIR spectrum (ATR) of 4-((4-allyl-2-methoxyphenoxy)methyl)-1-(*p*-tolyl)-1*H*-1,2,3-triazole (**2h**).

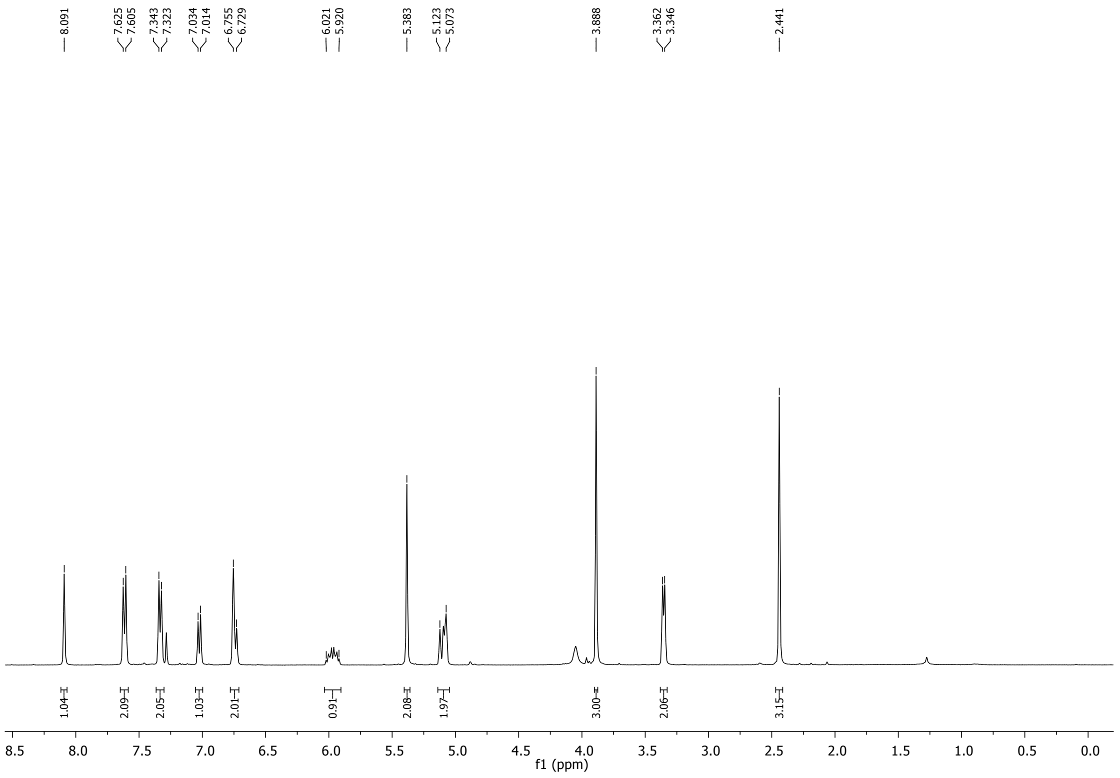

Figure S40. ^{1}H NMR spectrum (400 MHz, $CDCl_3$) of 4-((4-allyl-2-methoxyphenoxy)methyl)-1-(*p*-tolyl)-1*H*-1,2,3-triazole (**2h**).

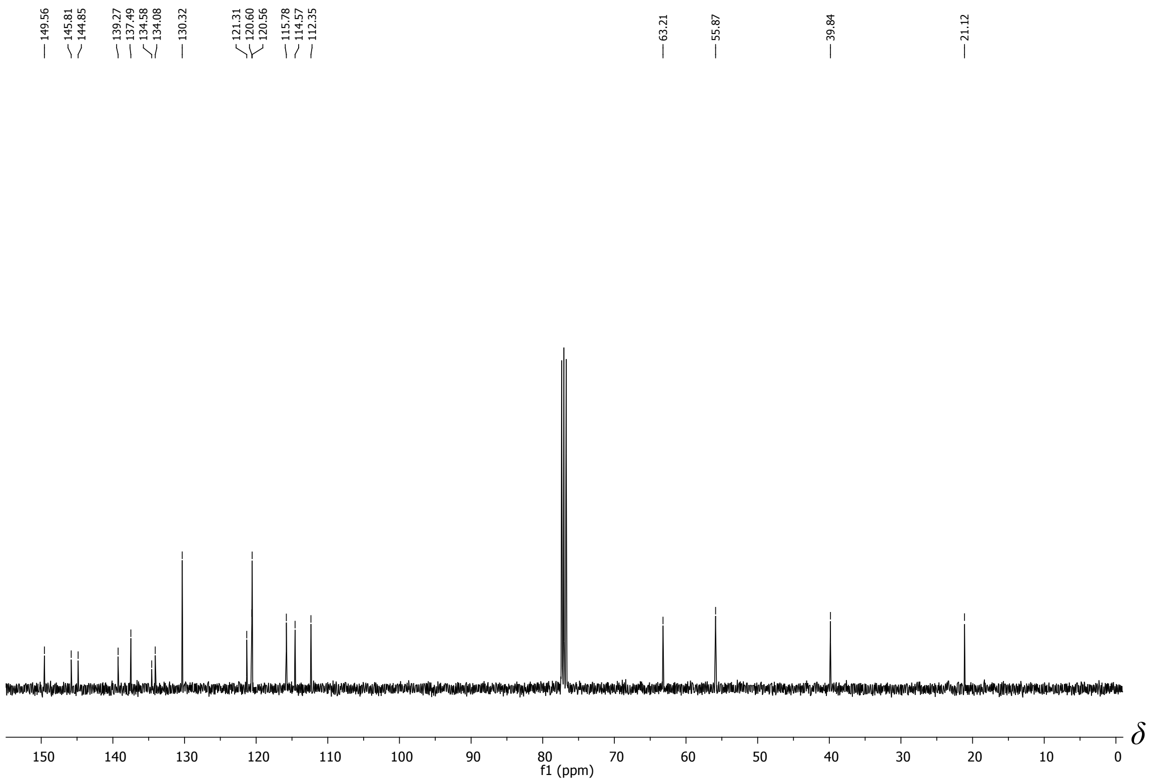

Figure S41. ^{13}C NMR spectrum (100 MHz, $CDCl_3$) of 4-((4-allyl-2-methoxyphenoxy)methyl)-1-(*p*-tolyl)-1*H*-1,2,3-triazole (**2h**).

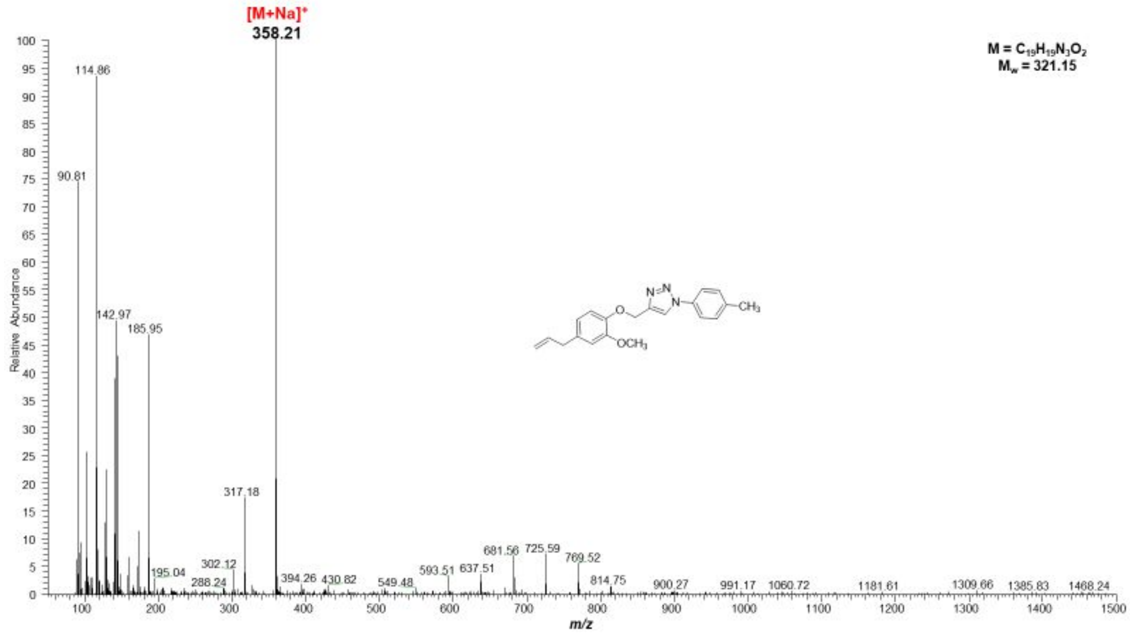

Figure S42. LC-MS spectrum of 4-((4-allyl-2-methoxyphenoxy)methyl)-1-(*p*-tolyl)-1*H*-1,2,3-triazole (**2h**).

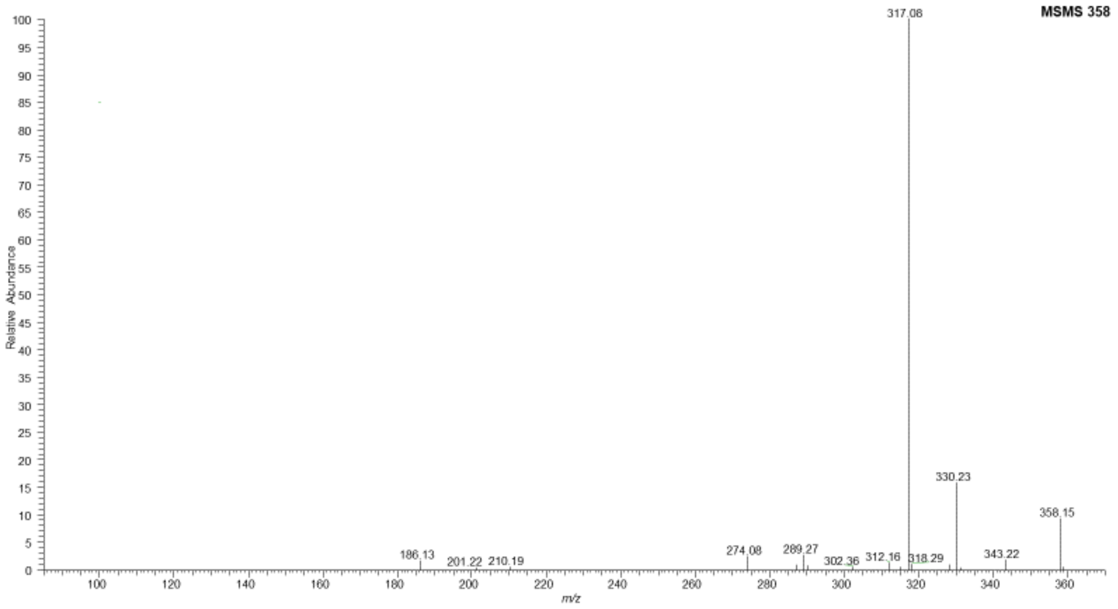

Figure S43. MS-MS spectrum of 4-((4-allyl-2-methoxyphenoxy)methyl)-1-(*p*-tolyl)-1*H*-1,2,3-triazole (**2h**).

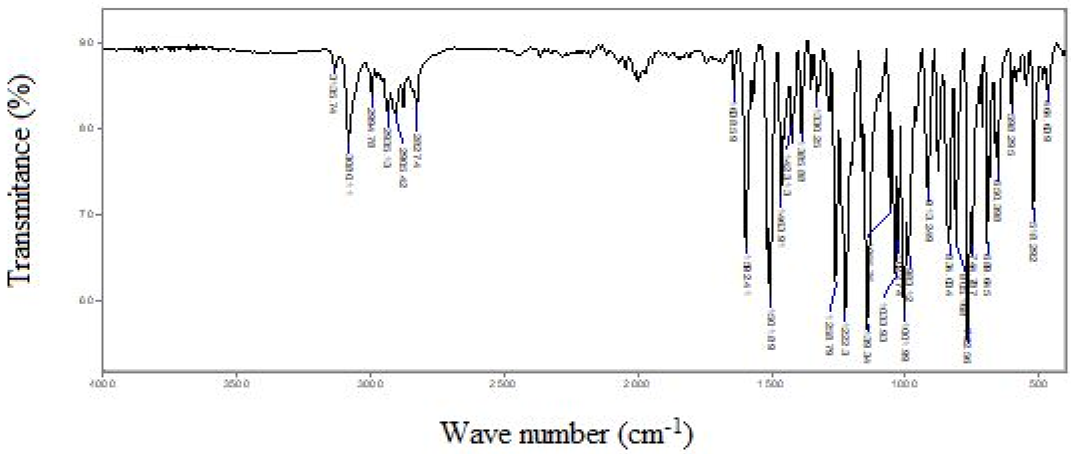

Figure S44. FTIR spectrum (ATR) of 4-((4-allyl-2-methoxyphenoxy)methyl)-1-phenyl-1*H*-1,2,3-triazole (**2i**).

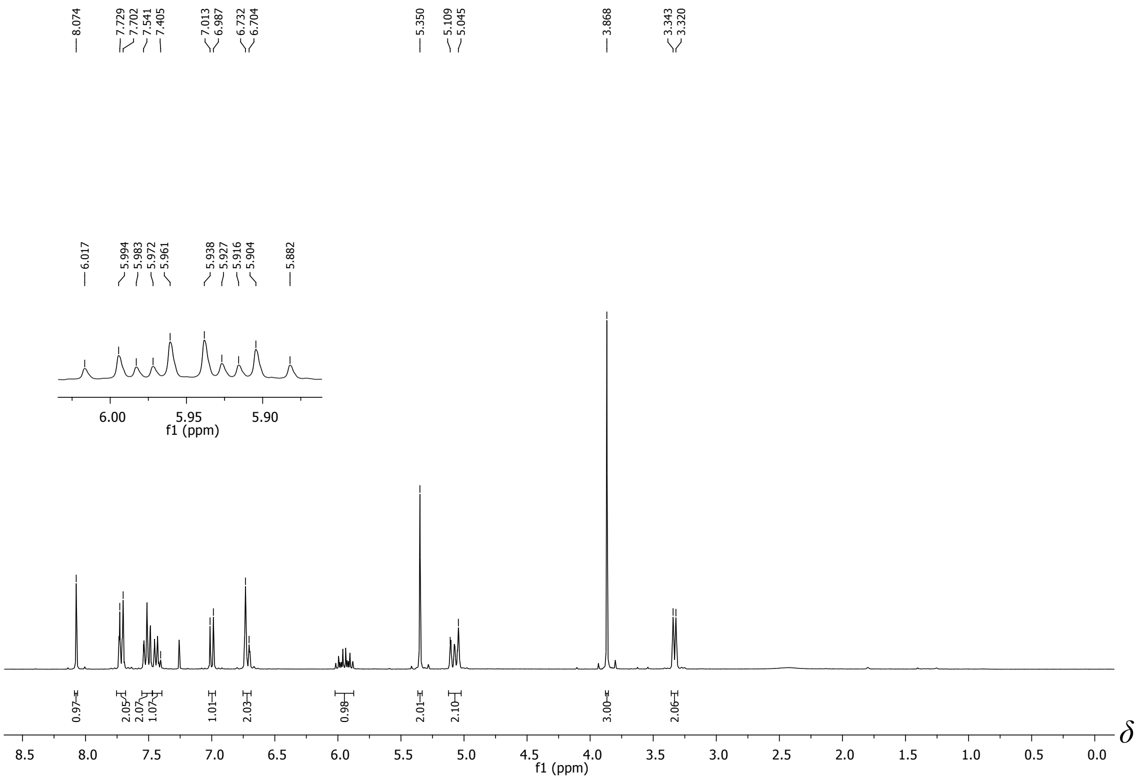

Figure S45. ^{1}H NMR spectrum (300 MHz, $CDCl_3$) of 4-((4-allyl-2-methoxyphenoxy)methyl)-1-phenyl-1*H*-1,2,3-triazole (**2i**).

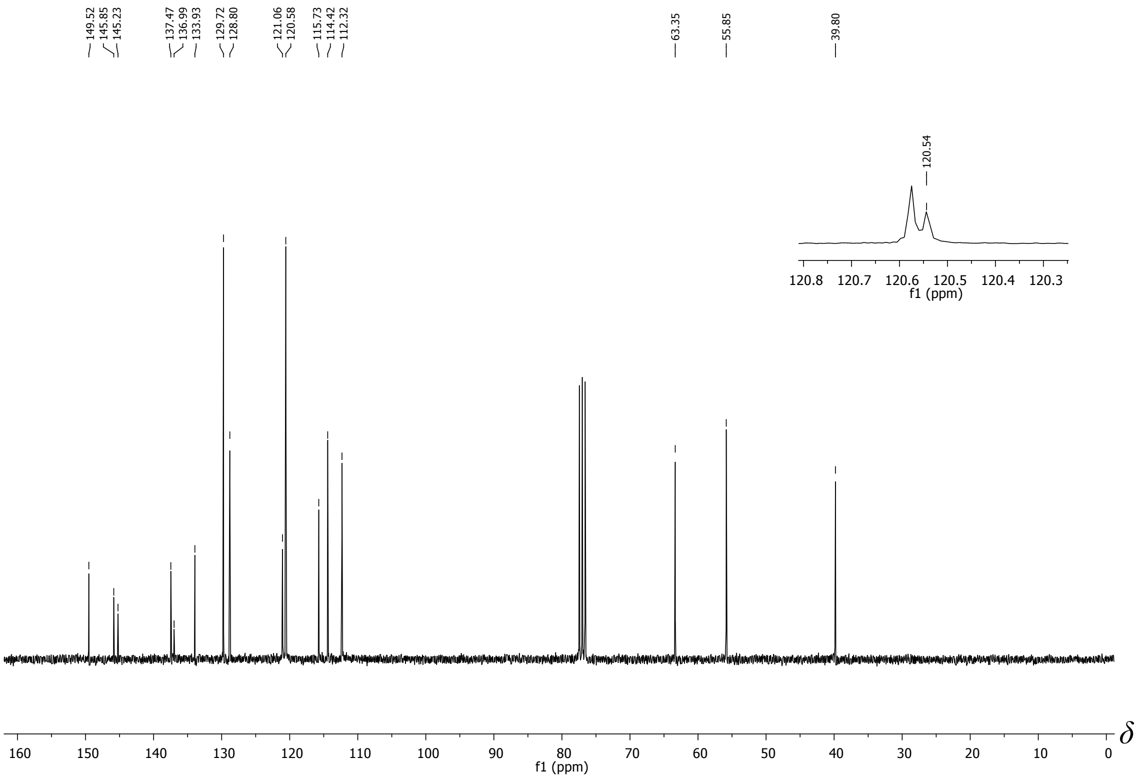

Figure S46. ^{13}C NMR spectrum (75 MHz, $CDCl_3$) of 4-((4-allyl-2-methoxyphenoxy)methyl)-1-phenyl-1*H*-1,2,3-triazole (**2i**).

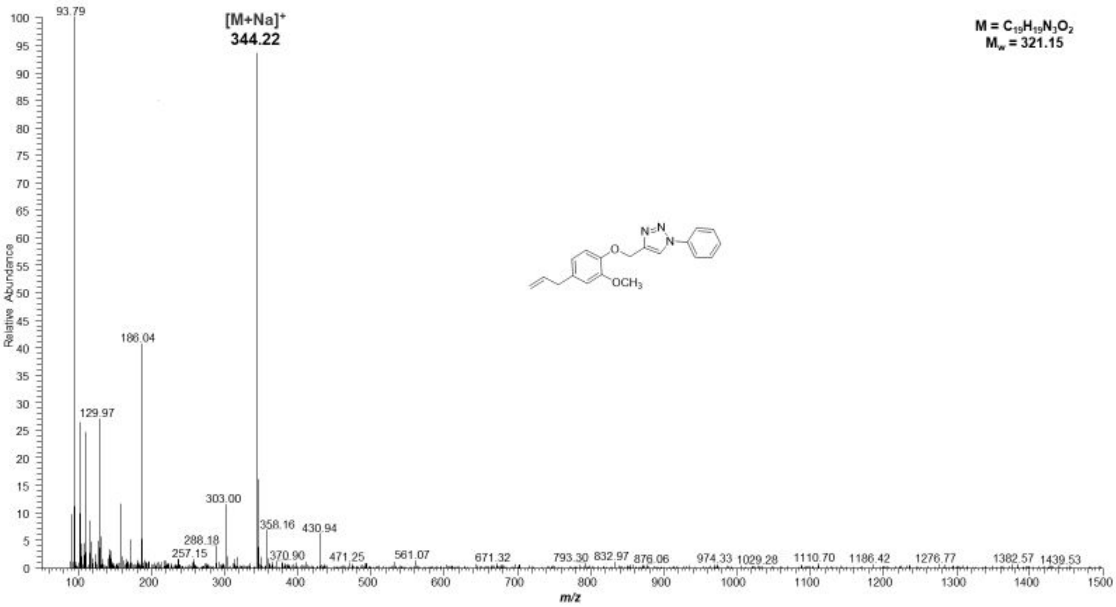

Figure S47. LC-MS spectrum of 4-((4-allyl-2-methoxyphenoxy)methyl)-1-phenyl-1*H*-1,2,3-triazole (**2i**).

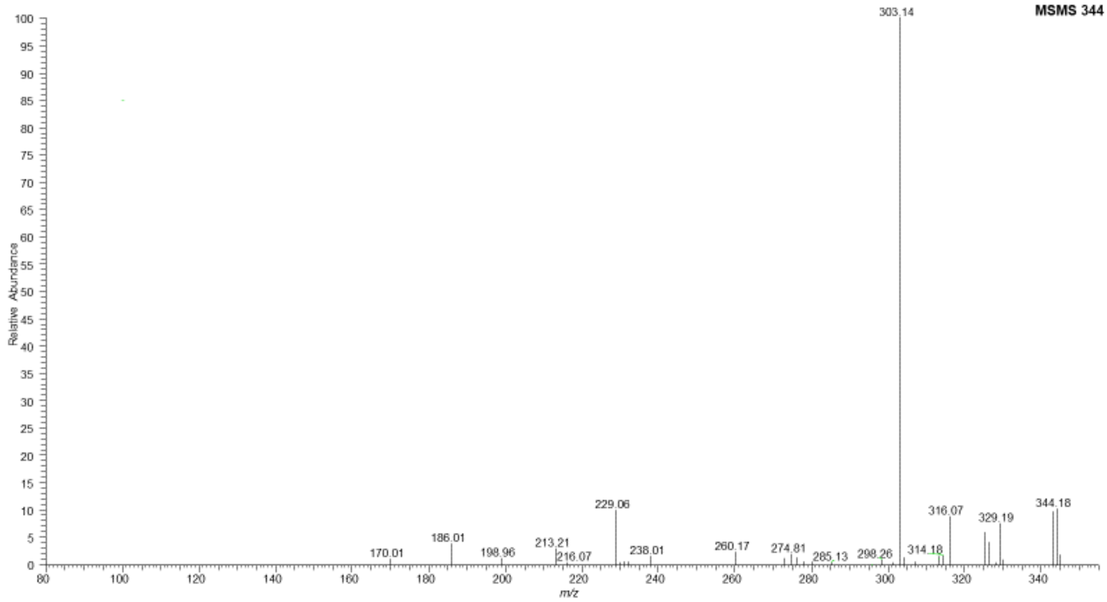

Figure S48. MS-MS spectrum of 4-((4-allyl-2-methoxyphenoxy)methyl)-1-phenyl-1*H*-1,2,3-triazole (**2i**).

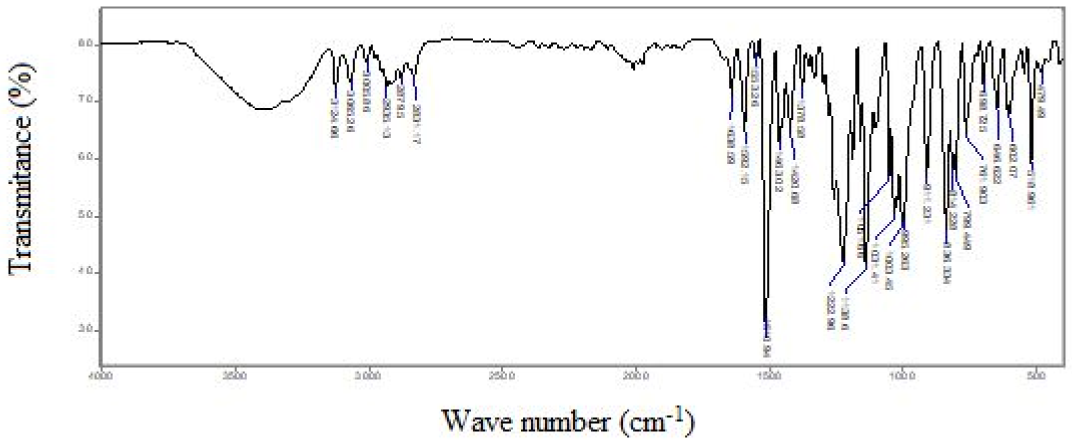

Figure S49. FTIR spectrum (ATR) of 4-((4-allyl-2-methoxyphenoxy)methyl)-1-(4-fluorophenyl)-1*H*-1,2,3-triazole (**2j**).

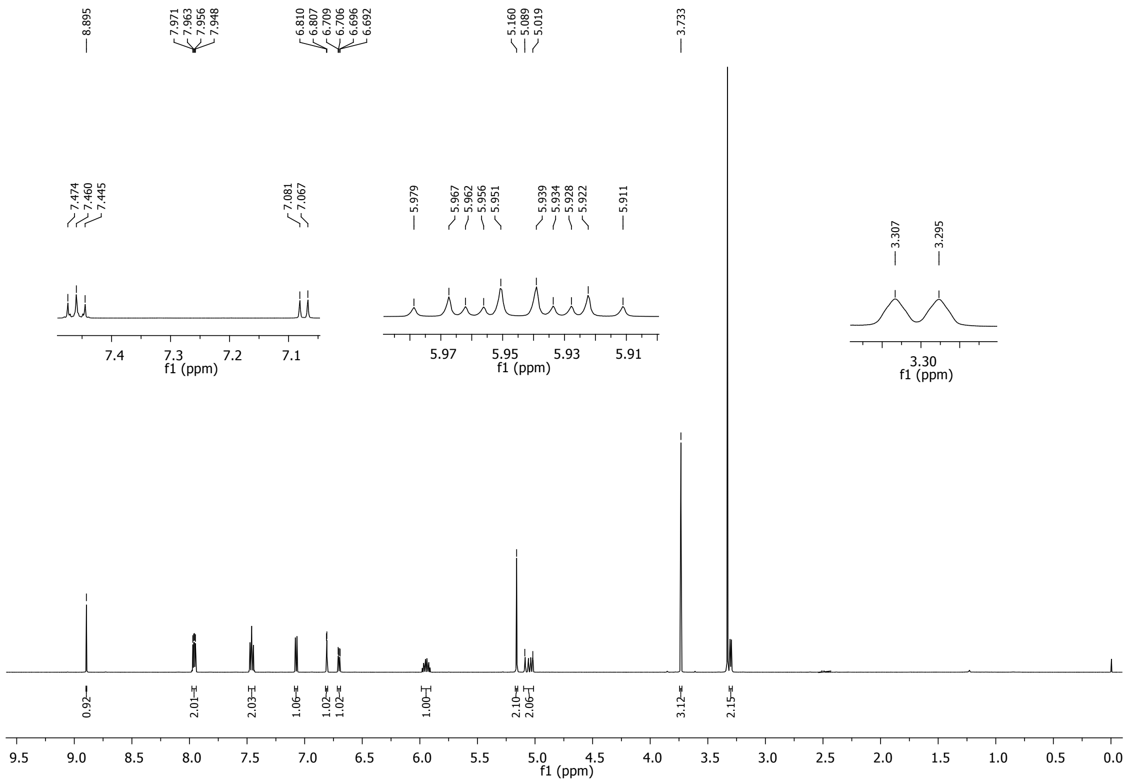

Figure S50. ^{1}H NMR spectrum (600 MHz, DMSO-d_6) of 4-((4-allyl-2-methoxyphenoxy)methyl)-1-(4-fluorophenyl)-1*H*-1,2,3-triazole (**2j**). δ

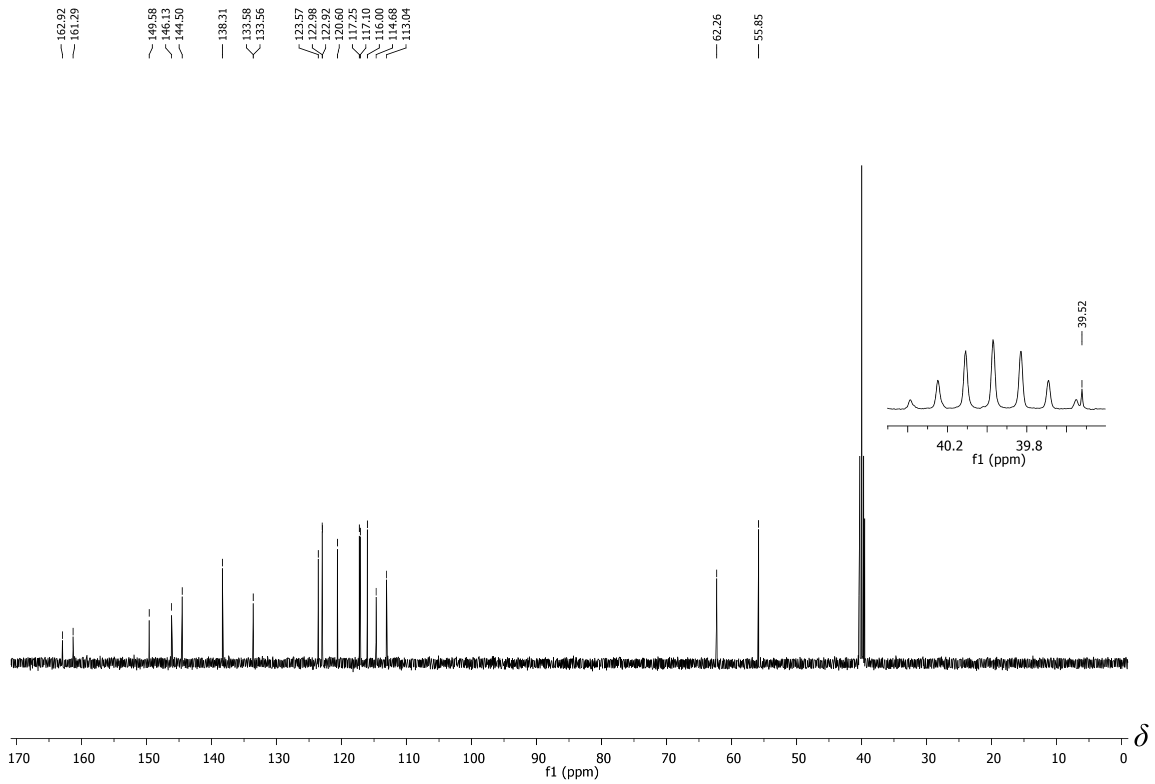

Figure S51. ^{13}C NMR spectrum (150 MHz, DMSO-d_6) of 4-((4-allyl-2-methoxyphenoxy)methyl)-1-(4-fluorophenyl)-1*H*-1,2,3-triazole (**2j**).

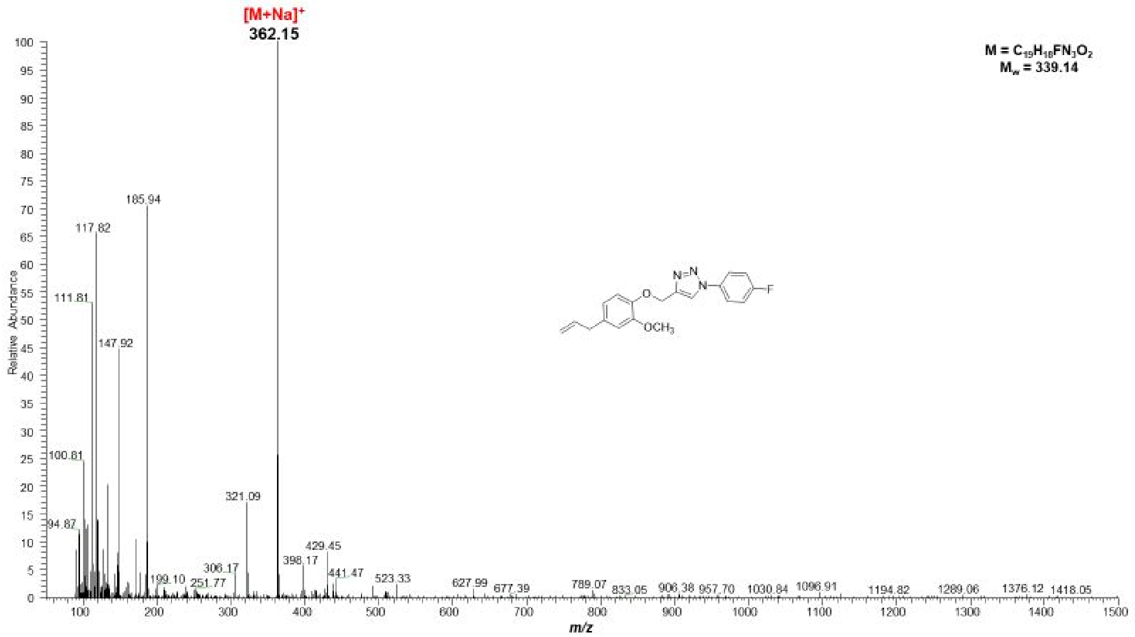

Figure S52. LC-MS spectrum of 4-((4-allyl-2-methoxyphenoxy)methyl)-1-(4-fluorophenyl)-1*H*-1,2,3-triazole (**2j**).

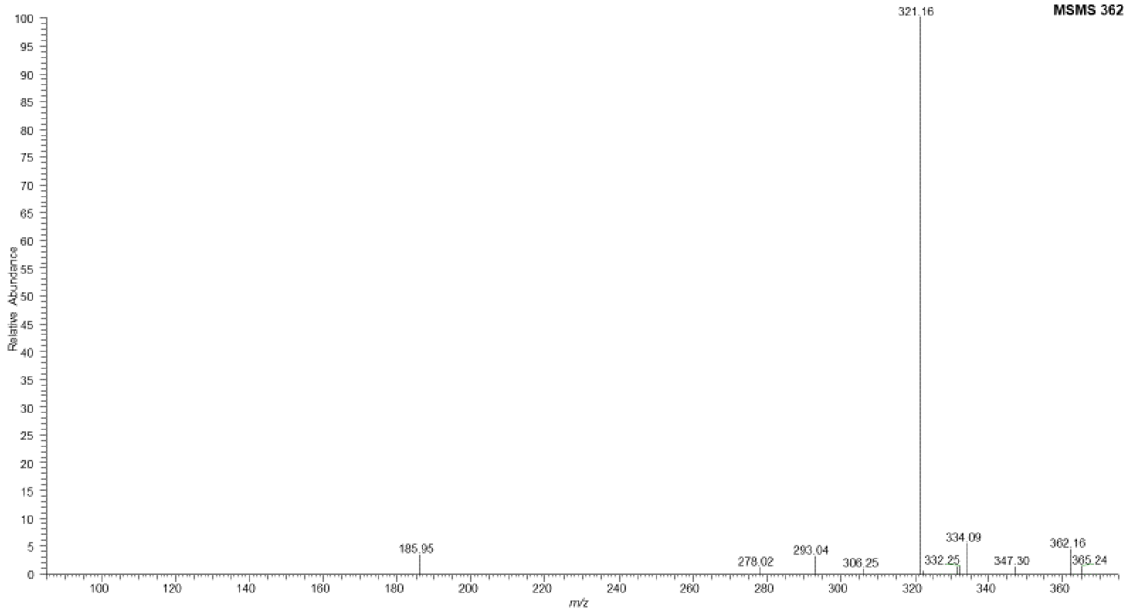

Figure S53. MS-MS spectrum of 4-((4-allyl-2-methoxyphenoxy)methyl)-1-(4-fluorophenyl)-1*H*-1,2,3-triazole (**2j**).

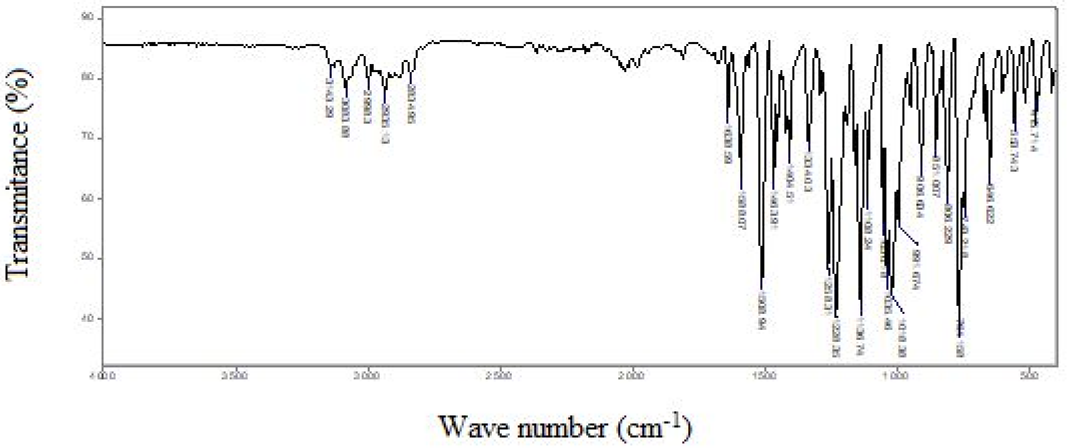

Figure S54. FTIR spectrum (ATR) of 4-((4-allyl-2-methoxyphenoxy)methyl)-1-(2-fluorophenyl)-1*H*-1,2,3-triazole (**2k**).

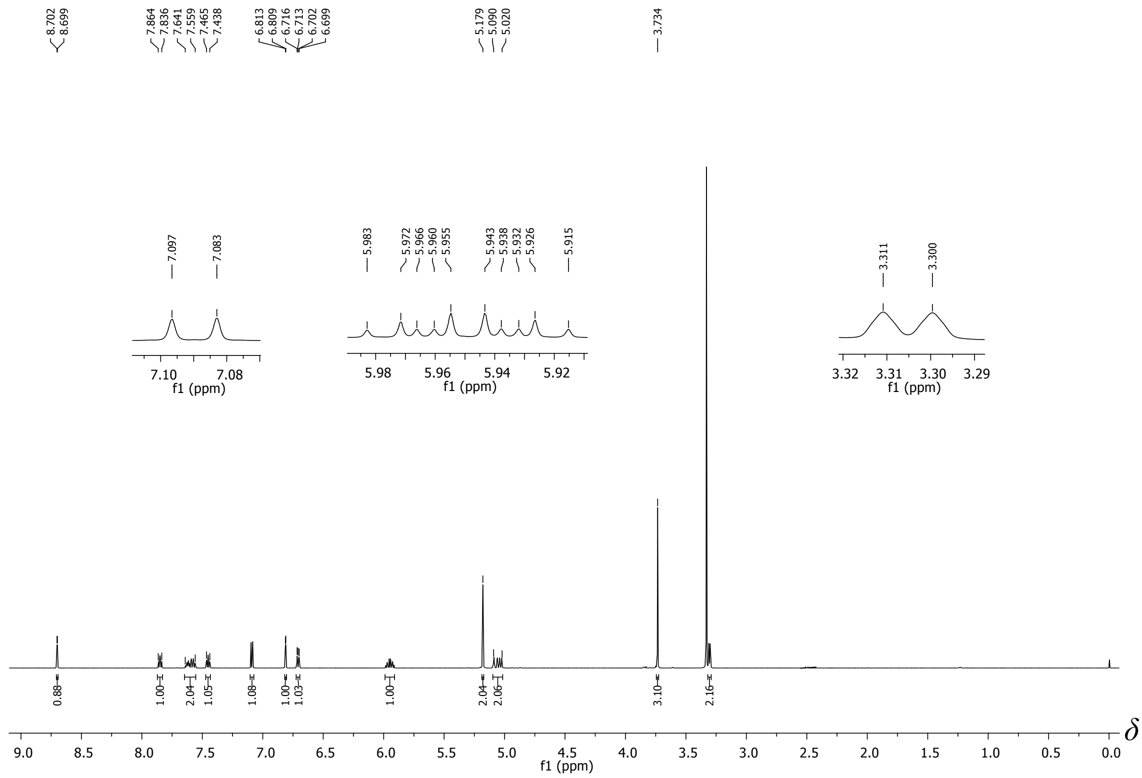

Figure S55. ^{1}H NMR spectrum (600 MHz, DMSO-d_6) of 4-((4-allyl-2-methoxyphenoxy)methyl)-1-(2-fluorophenyl)-1*H*-1,2,3-triazole (**2k**).

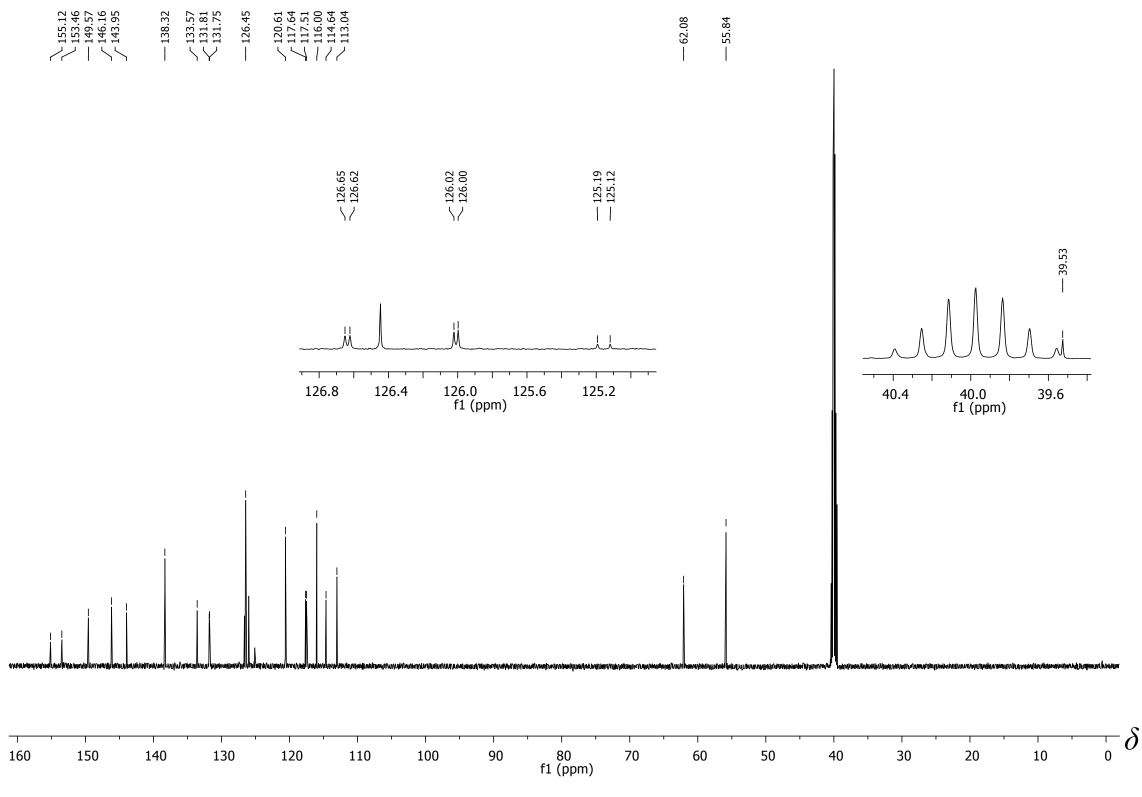

Figure S56. ^{13}C NMR spectrum (150 MHz, DMSO-d_6) of 4-((4-allyl-2-methoxyphenoxy)methyl)-1-(2-fluorophenyl)-1*H*-1,2,3-triazole (**2k**).

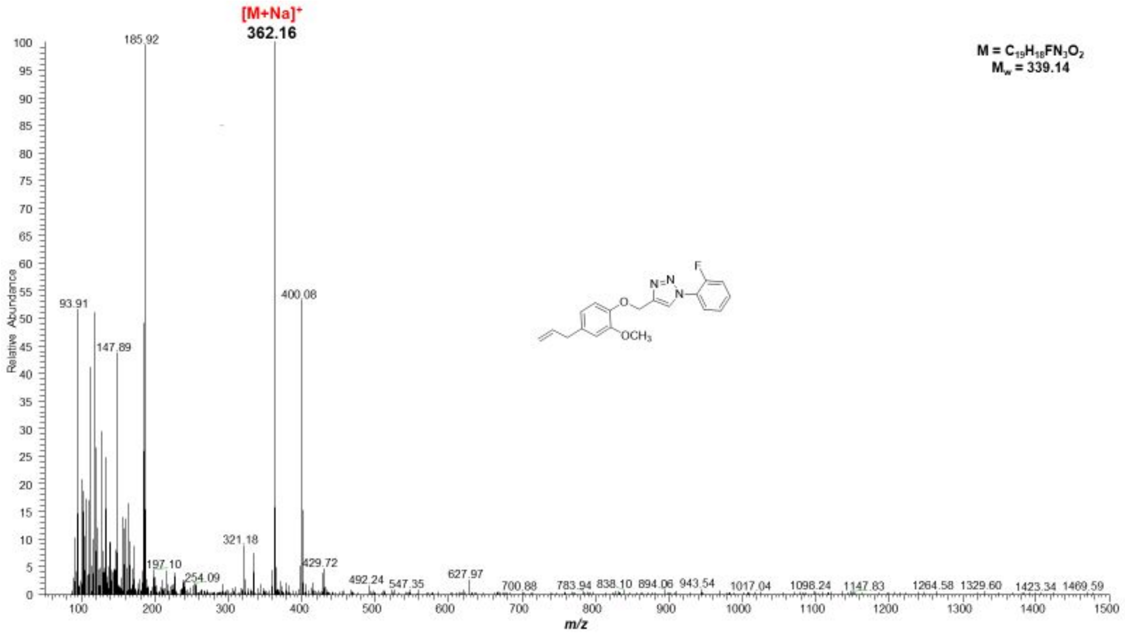

Figure S57. LC-MS spectrum of 4-((4-allyl-2-methoxyphenoxy)methyl)-1-(2-fluorophenyl)-1*H*-1,2,3-triazole (**2k**).

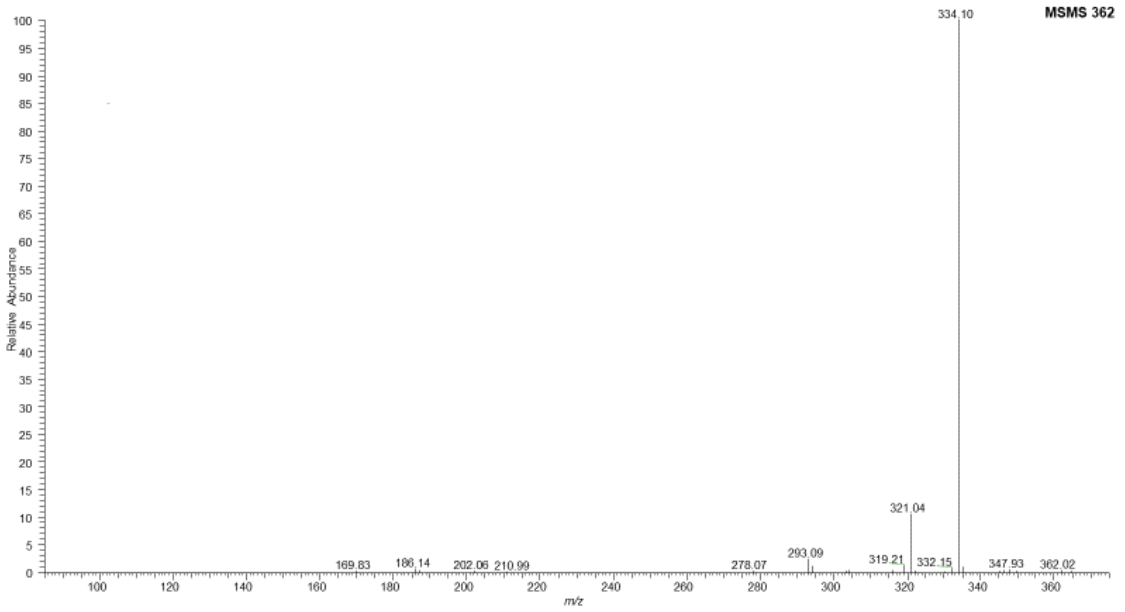

Figure S58. MS-MS spectrum of 4-((4-allyl-2-methoxyphenoxy)methyl)-1-(2-fluorophenyl)-1*H*-1,2,3-triazole (**2k**).

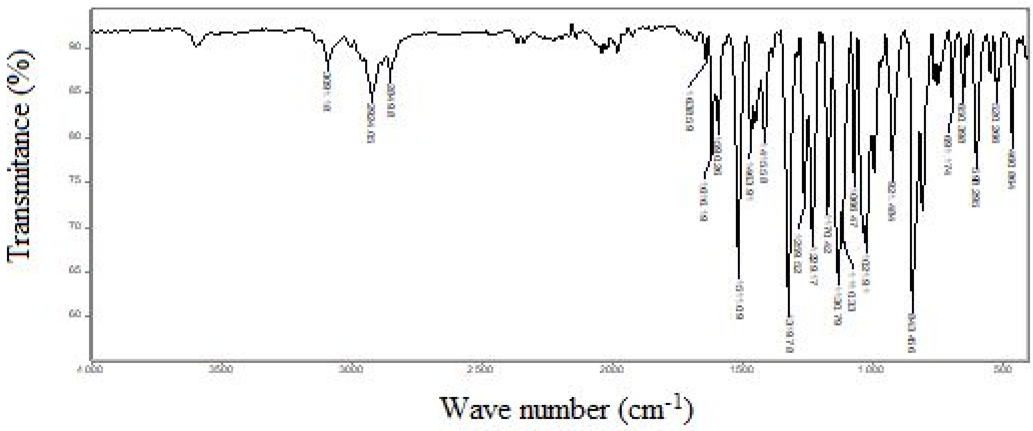

Figure S59. FTIR spectrum (ATR) of 4-((4-allyl-2-methoxyphenoxy)methyl)-1-(4-(trifluoromethyl)phenyl)-1H-1,2,3-triazole (**2l**).

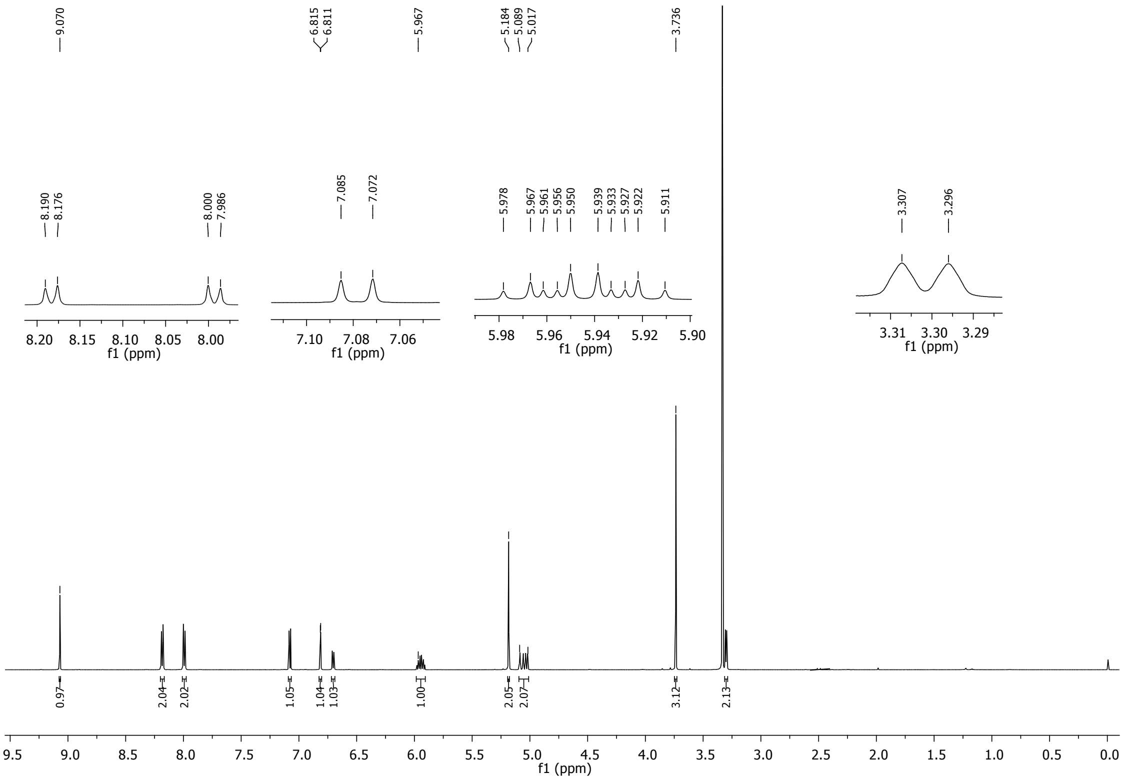

Figure S60. ^{1}H NMR spectrum (600 MHz, DMSO-d_6) of 4-((4-allyl-2-methoxyphenoxy)methyl)-1-(4-(trifluoromethyl)phenyl)-1H-1,2,3-triazole (**2l**).

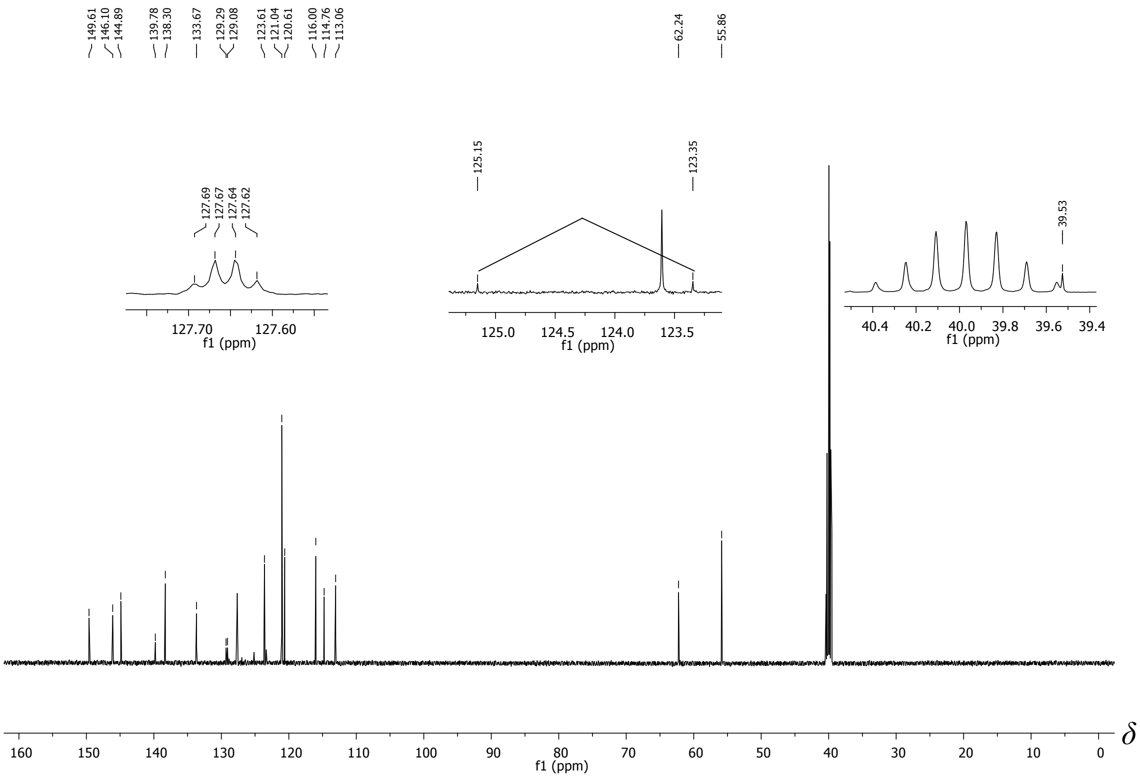

Figure S61. ^{13}C NMR spectrum (150 MHz, DMSO-d_6) of 4-((4-allyl-2-methoxyphenoxy)methyl)-1-(4-(trifluoromethyl)phenyl)-1*H*-1,2,3-triazole (**2l**).

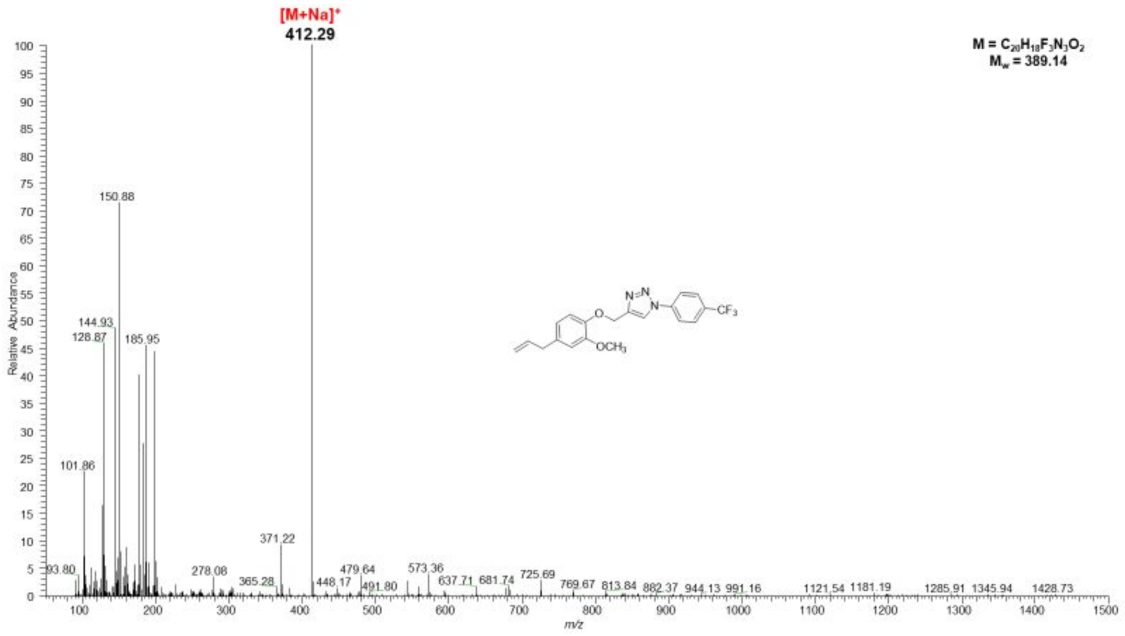

Figure S62. LC-MS spectrum of 4-((4-allyl-2-methoxyphenoxy)methyl)-1-(4-(trifluoromethyl)phenyl)-1*H*-1,2,3-triazole (**2l**).

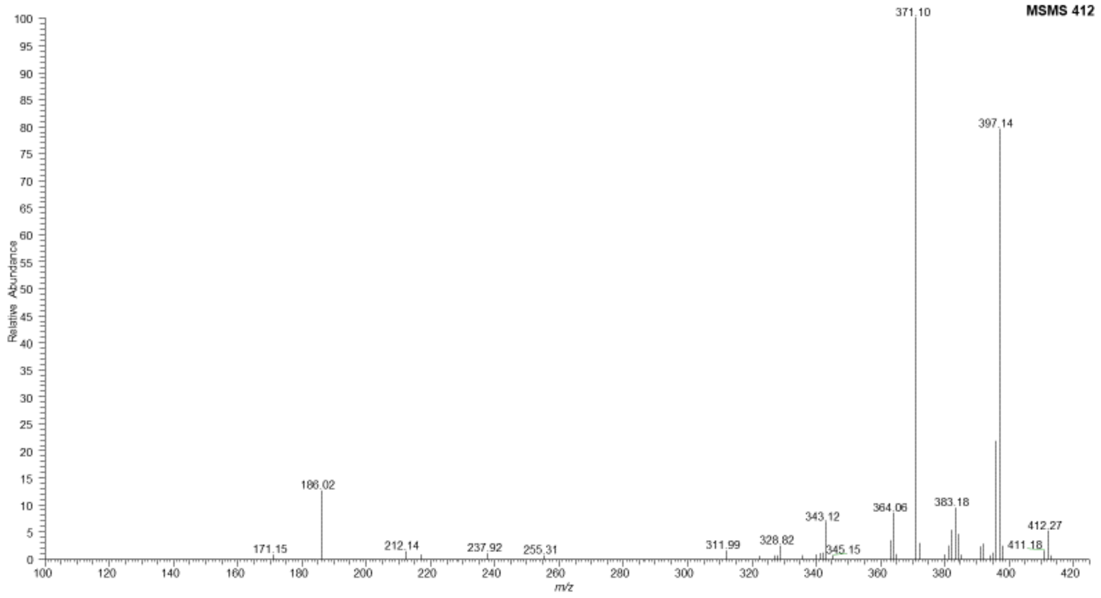

Figure S63. MS-MS spectrum of 4-((4-allyl-2-methoxyphenoxy)methyl)-1-(4-(trifluoromethyl)phenyl)-1*H*-1,2,3-triazole (**2l**).

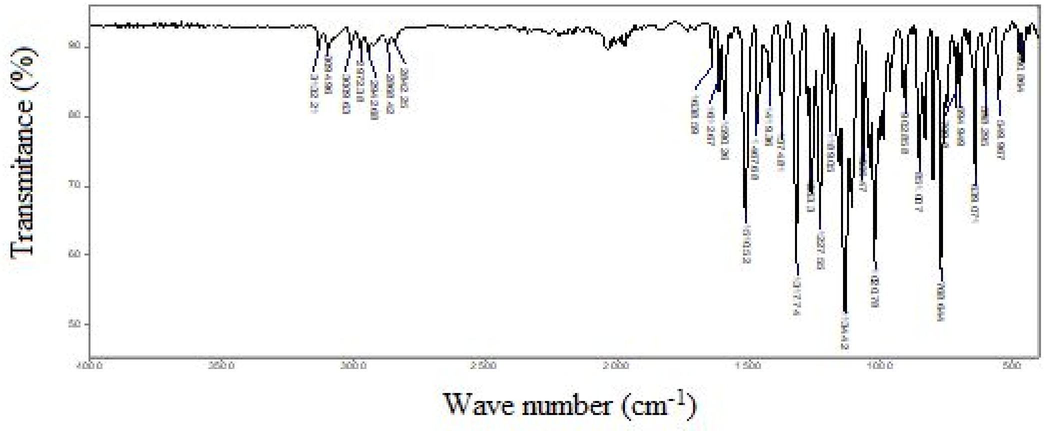

Figure S64. FTIR spectrum (ATR) of 4-((4-allyl-2-methoxyphenoxy)methyl)-1-(2-(trifluoromethyl)phenyl)-1*H*-1,2,3-triazole (**2m**).

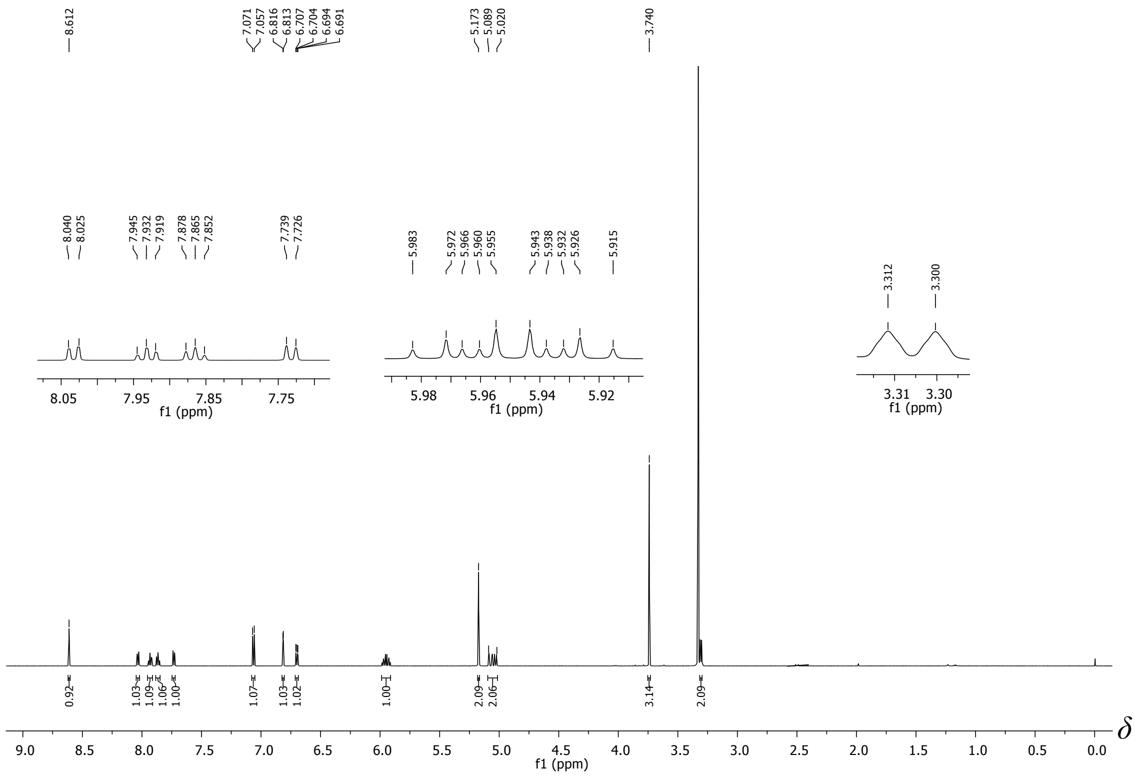

Figure S65. ^{1}H NMR spectrum (600 MHz, DMSO-d_6) of 4-((4-allyl-2-methoxyphenoxy)methyl)-1-(2-(trifluoromethyl)phenyl)-1*H*-1,2,3-triazole (**2m**).

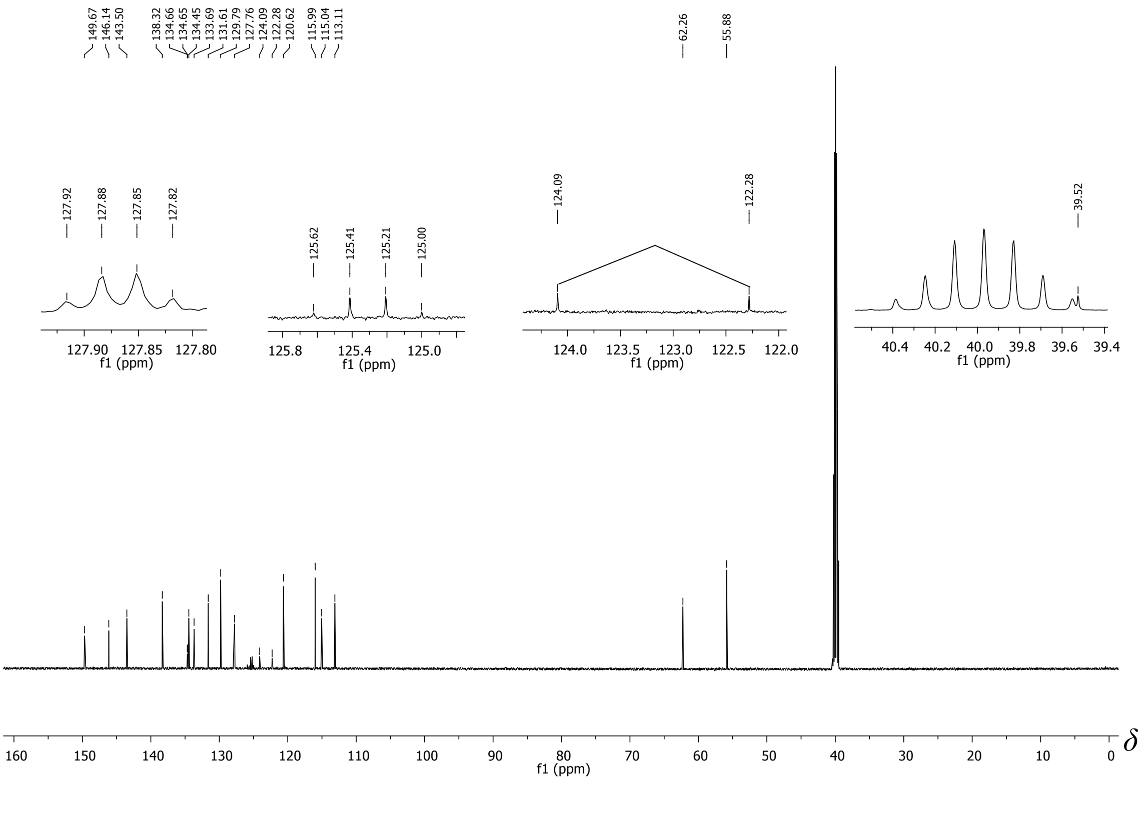

Figure S66. ^{13}C NMR spectrum (150 MHz, DMSO-d_6) of 4-((4-allyl-2-methoxyphenoxy)methyl)-1-(2-(trifluoromethyl)phenyl)-1*H*-1,2,3-triazole (**2m**).

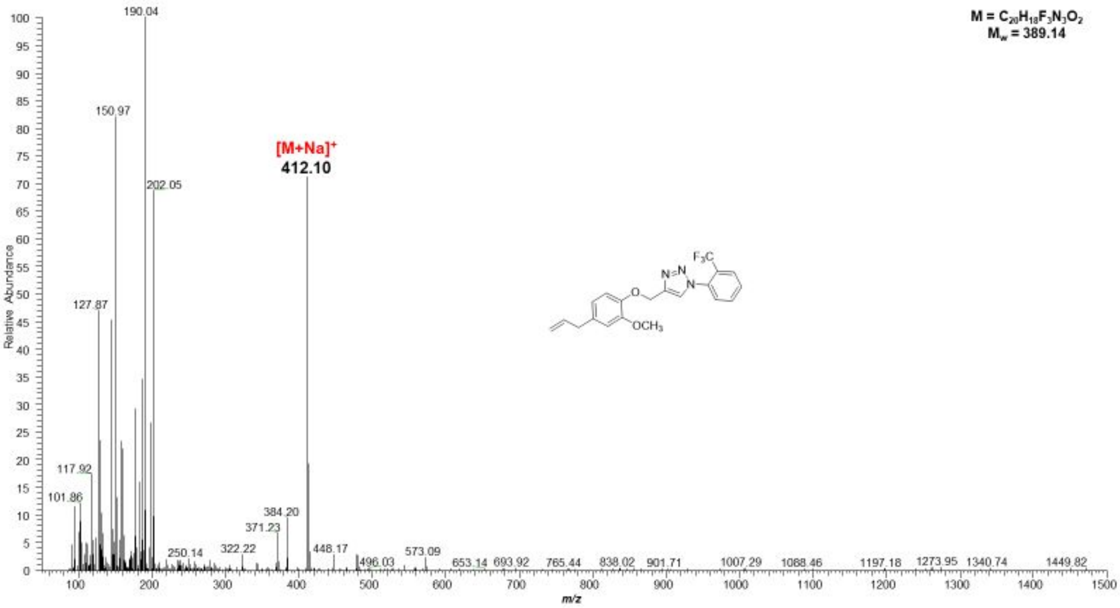

Figure S67. LC-MS spectrum of 4-((4-allyl-2-methoxyphenoxy)methyl)-1-(2-(trifluoromethyl)phenyl)-1*H*-1,2,3-triazole (**2m**).

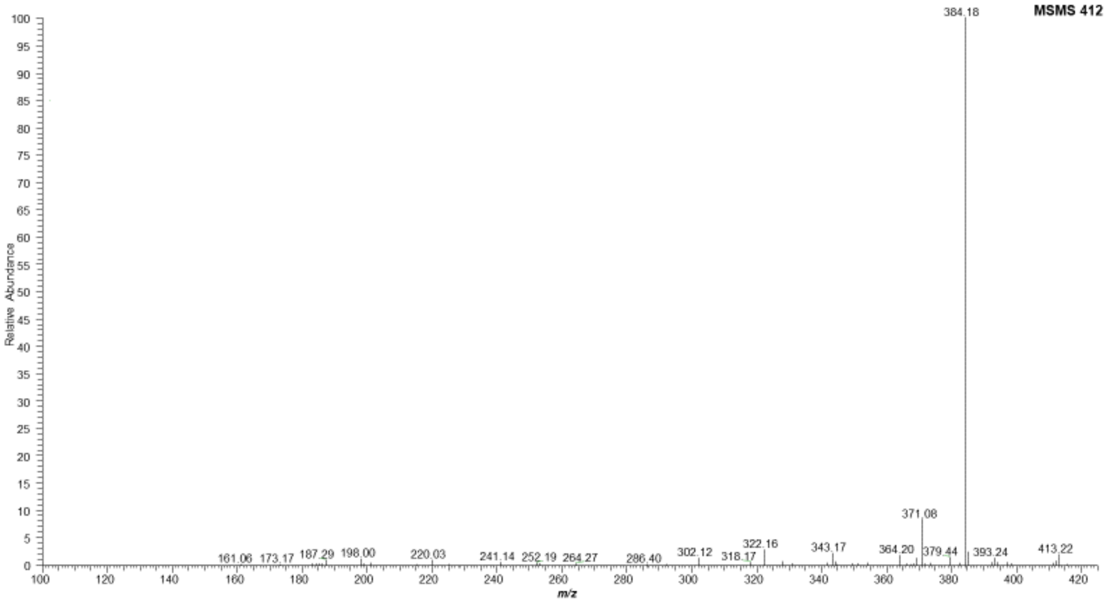

Figure S68. MS-MS spectrum of 4-((4-allyl-2-methoxyphenoxy)methyl)-1-(2-(trifluoromethyl)phenyl)-1*H*-1,2,3-triazole (**2m**).

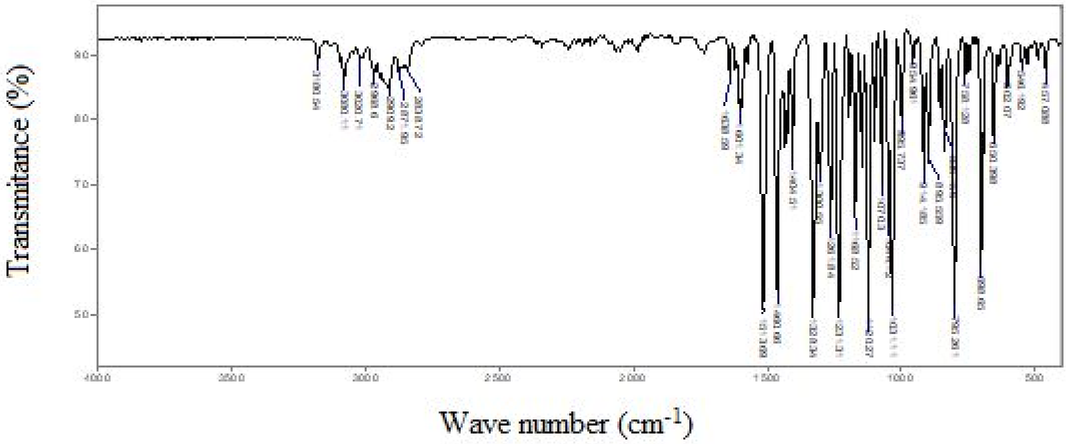

Figure S69. FTIR spectrum (ATR) of 4-((4-allyl-2-methoxyphenoxy)methyl)-1-(3-(trifluoromethyl)phenyl)-1*H*-1,2,3-triazole (**2n**).

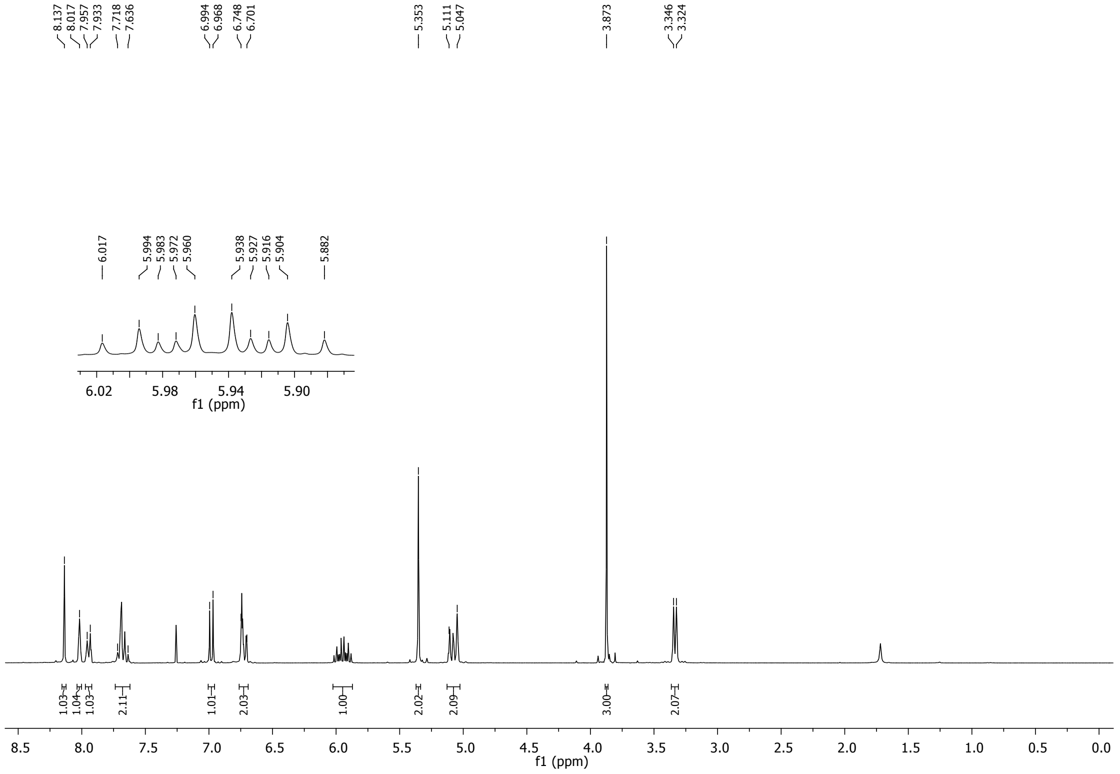

Figure S70. ^{1}H NMR spectrum (300 MHz, $CDCl_3$) of 4-((4-allyl-2-methoxyphenoxy)methyl)-1-(3-(trifluoromethyl)phenyl)-1*H*-1,2,3-triazole (**2n**).

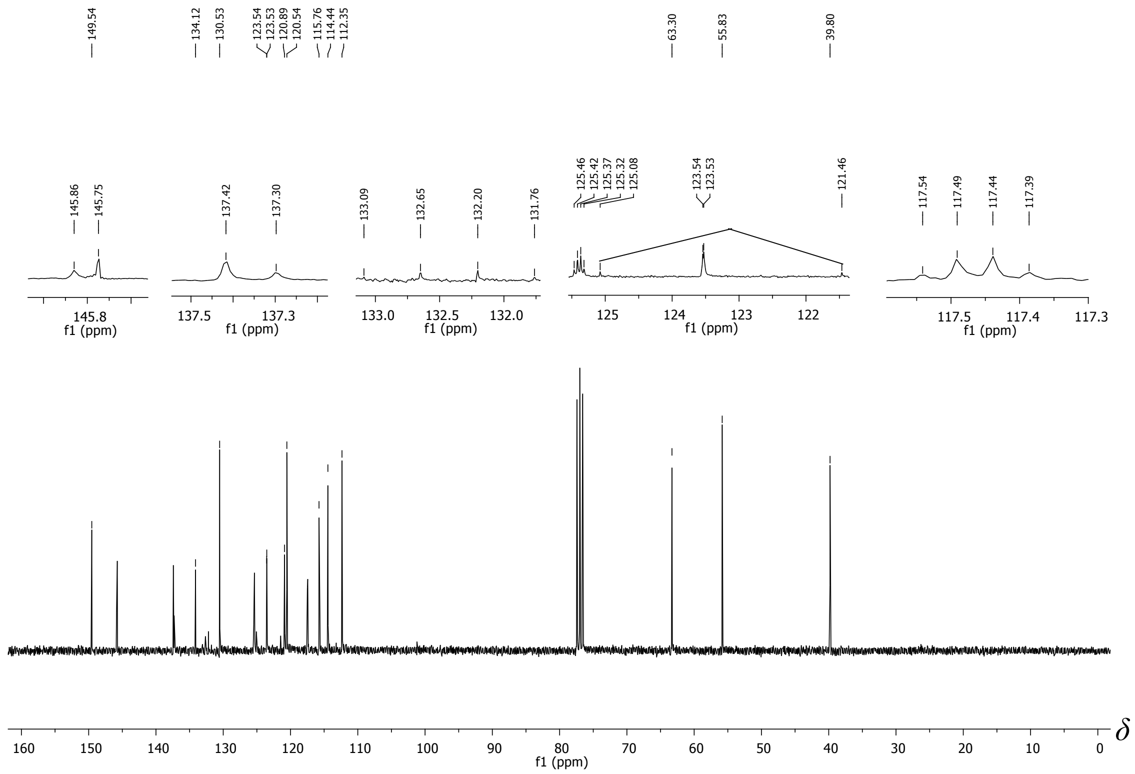

Figure S71. ^{13}C NMR spectrum (75 MHz, $CDCl_3$) of 4-((4-allyl-2-methoxyphenoxy)methyl)-1-(3-(trifluoromethyl)phenyl)-1*H*-1,2,3-triazole (**2n**).

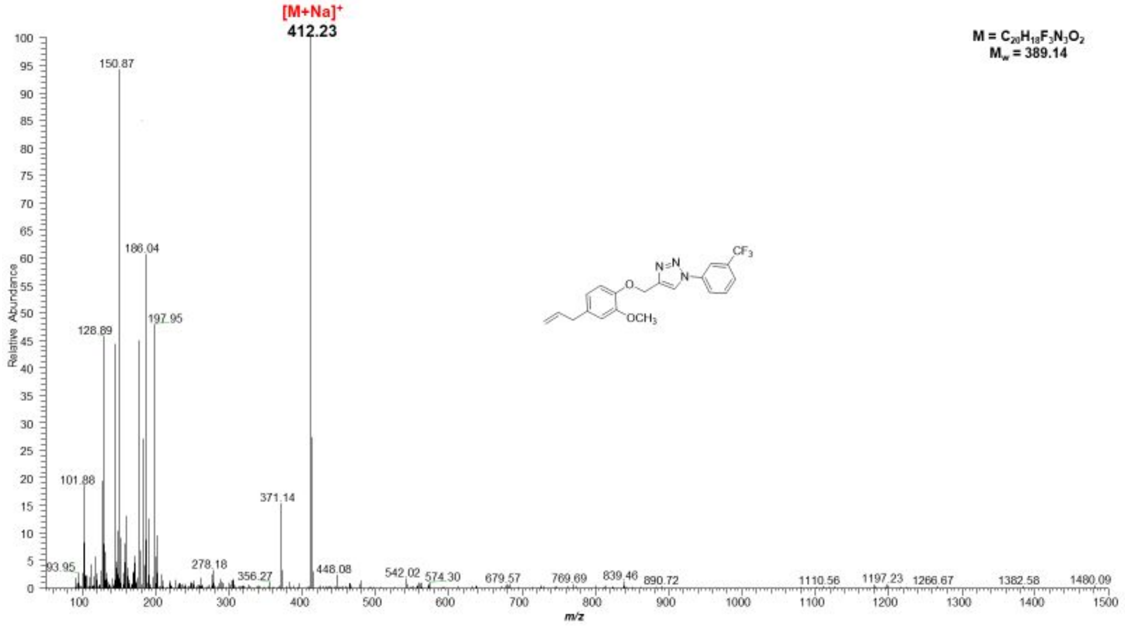

Figure S72. LC-MS spectrum of 4-((4-allyl-2-methoxyphenoxy)methyl)-1-(3-(trifluoromethyl)phenyl)-1*H*-1,2,3-triazole (**2n**).

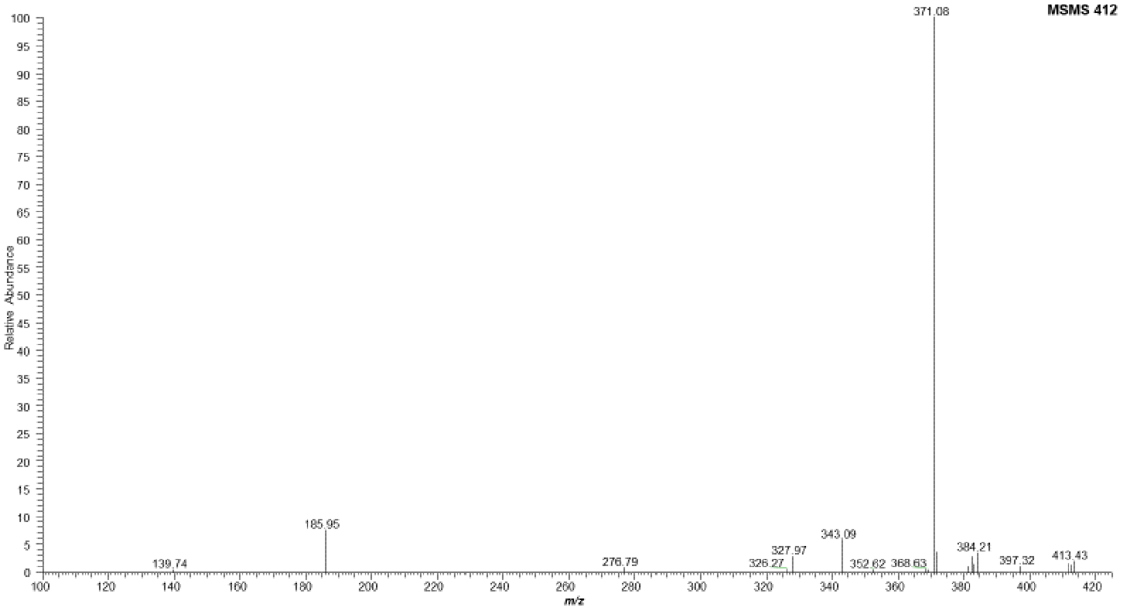

Figure S73. MS-MS spectrum of 4-((4-allyl-2-methoxyphenoxy)methyl)-1-(3-(trifluoromethyl)phenyl)-1*H*-1,2,3-triazole (**2n**).

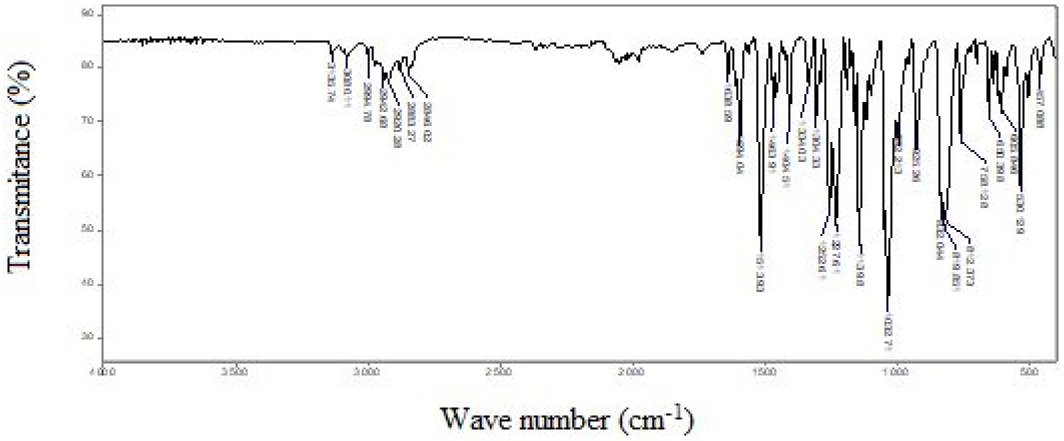

Figure S74. FTIR spectrum (ATR) of 4-((4-allyl-2-methoxyphenoxy)methyl)-1-(4-methoxyphenyl)-1*H*-1,2,3-triazole (**2o**).

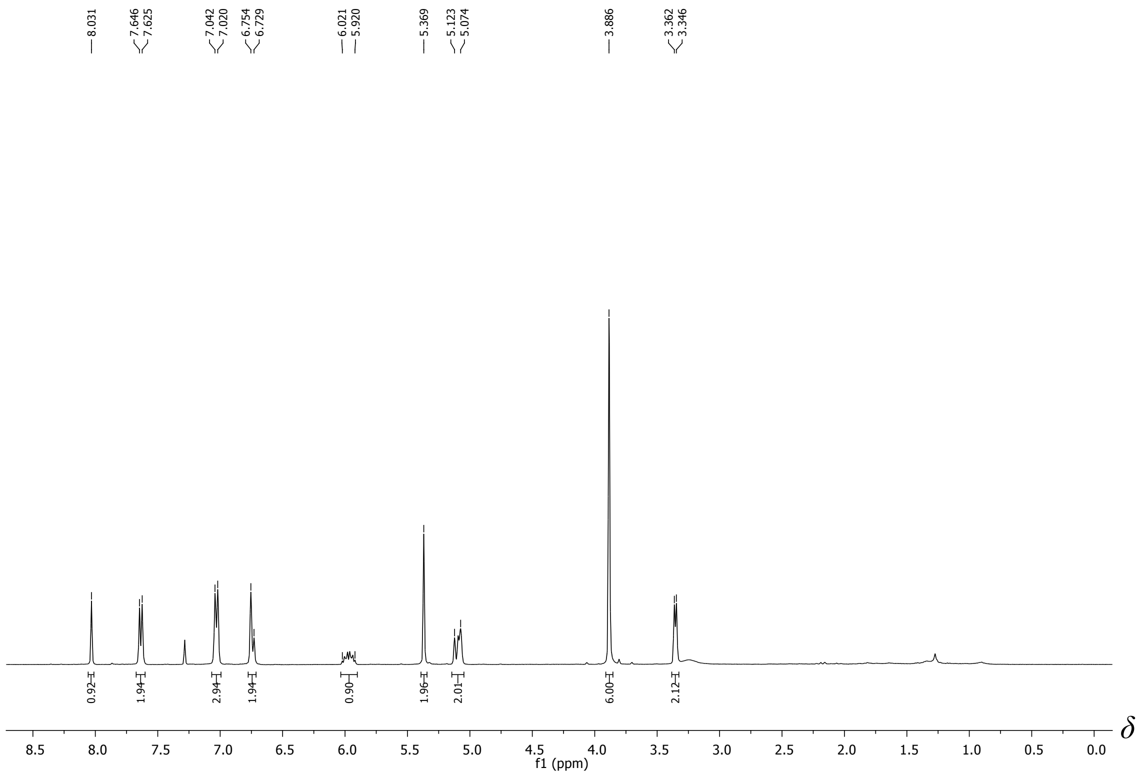

Figure S75. ^{1}H NMR spectrum (400 MHz, $CDCl_3$) of 4-((4-allyl-2-methoxyphenoxy)methyl)-1-(4-methoxyphenyl)-1*H*-1,2,3-triazole (**2o**).

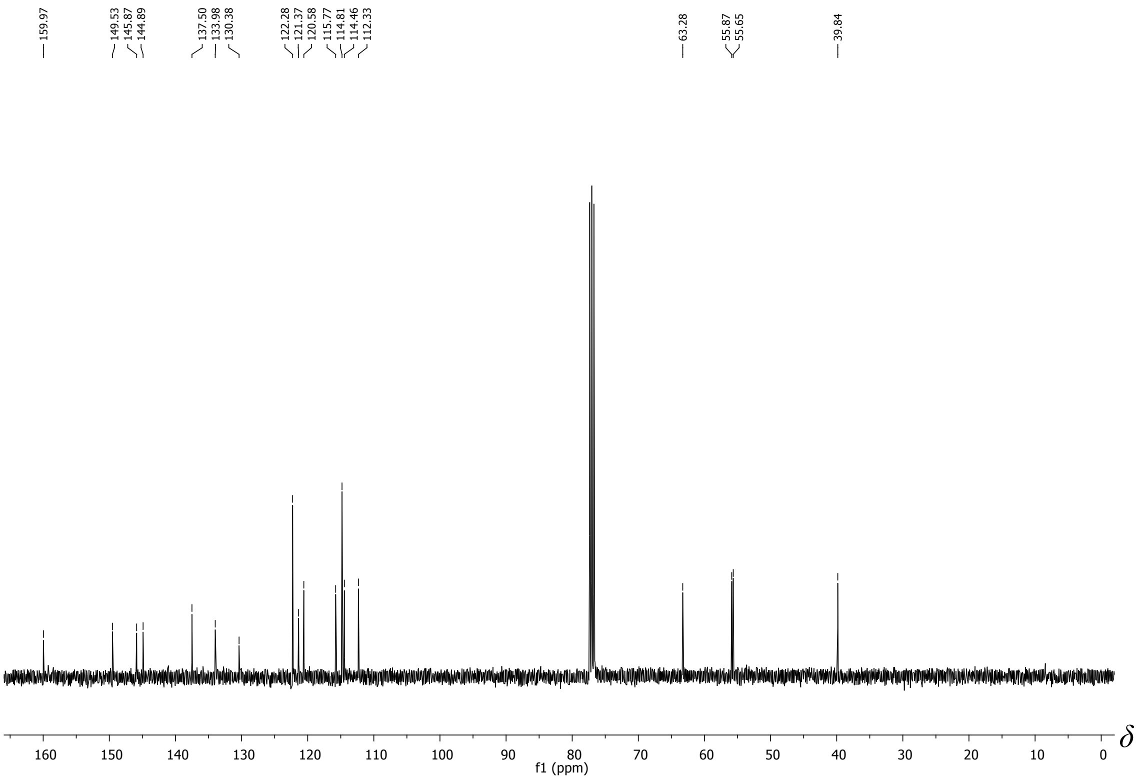

Figure S76. ^{13}C NMR spectrum (100 MHz, $CDCl_3$) of 4-((4-allyl-2-methoxyphenoxy)methyl)-1-(4-methoxyphenyl)-1*H*-1,2,3-triazole (**2o**).

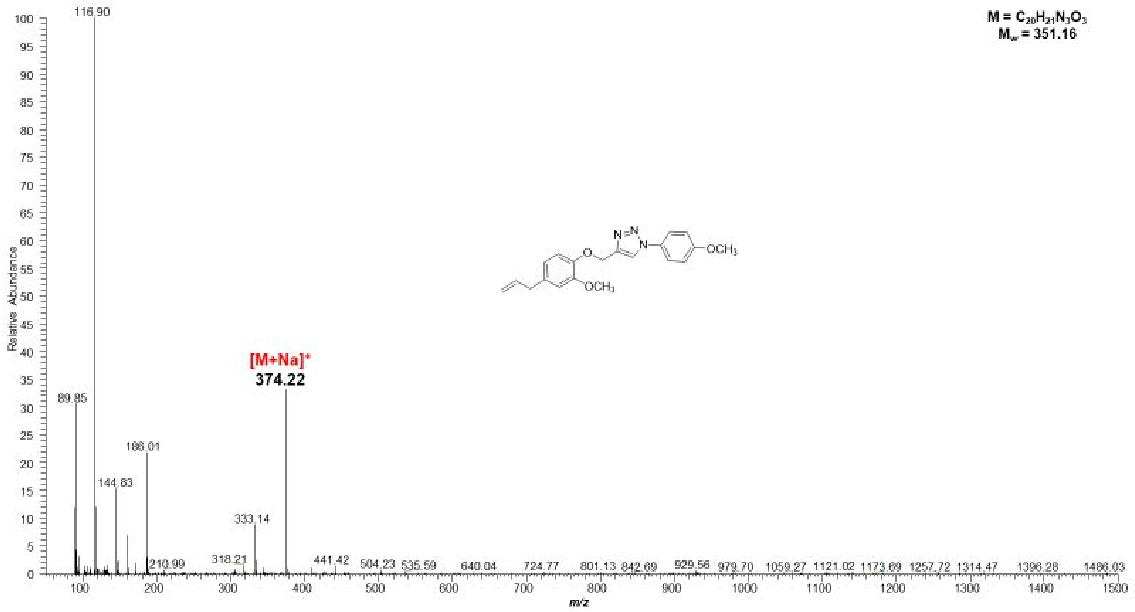

Figure S77. LC-MS spectrum of 4-((4-allyl-2-methoxyphenoxy)methyl)-1-(4-methoxyphenyl)-1*H*-1,2,3-triazole (**2o**).

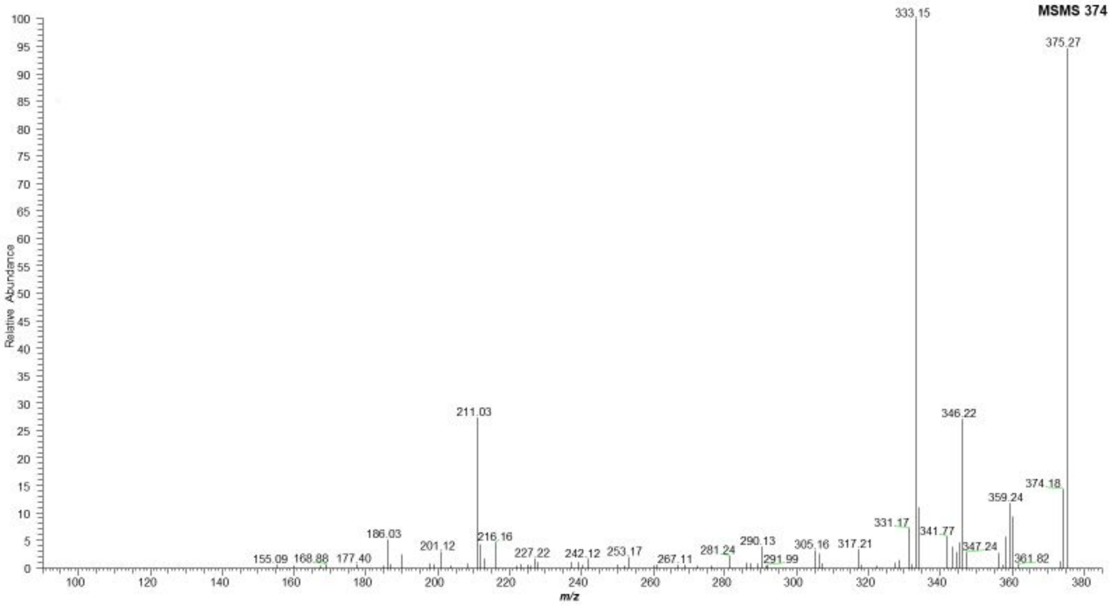

Figure S78. MS-MS spectrum of 4-((4-allyl-2-methoxyphenoxy)methyl)-1-(4-methoxyphenyl)-1*H*-1,2,3-triazole (**2o**).

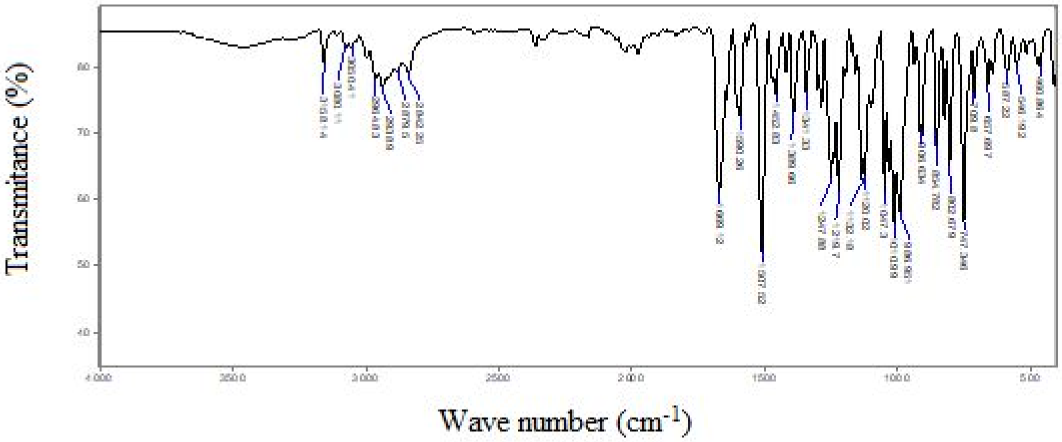

Figure S79. FTIR spectrum (ATR) of 4-((4-allyl-2-methoxyphenoxy)methyl)-1-(2-methoxyphenyl)-1*H*-1,2,3-triazole (**2p**).

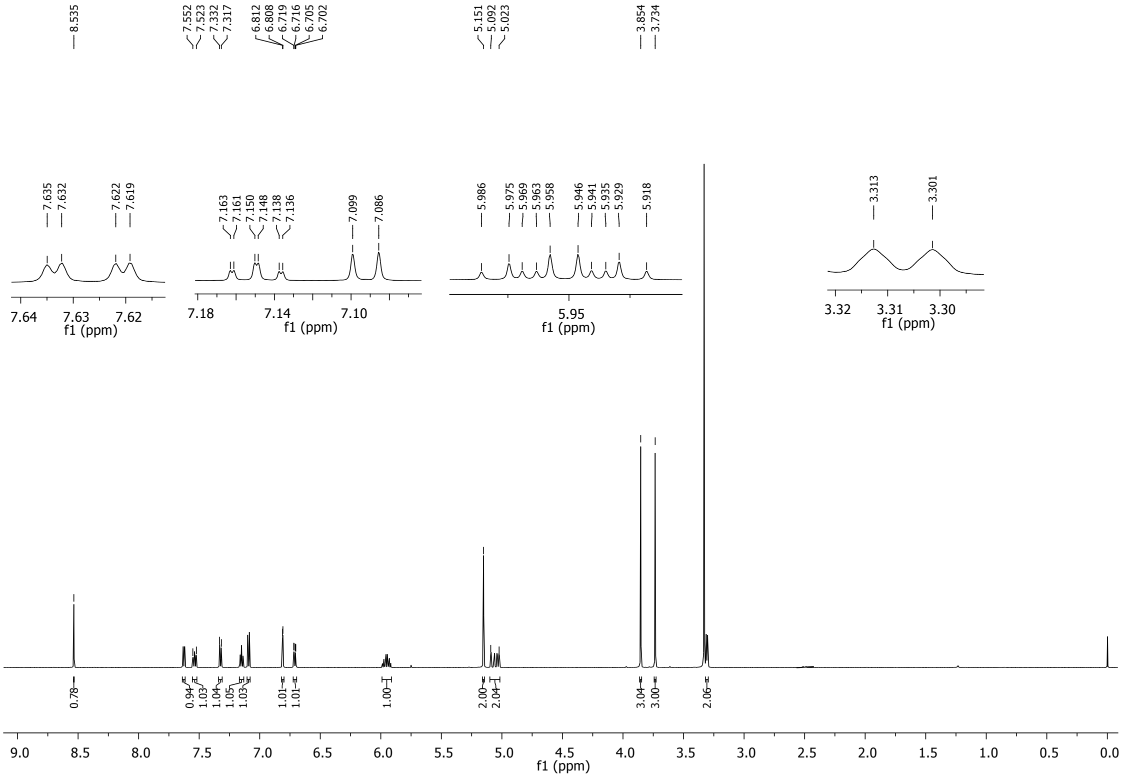

Figure S80. ^{1}H NMR spectrum (600 MHz, DMSO-d_6) of 4-((4-allyl-2-methoxyphenoxy)methyl)-1-(2-methoxyphenyl)-1*H*-1,2,3-triazole (**2p**).

δ

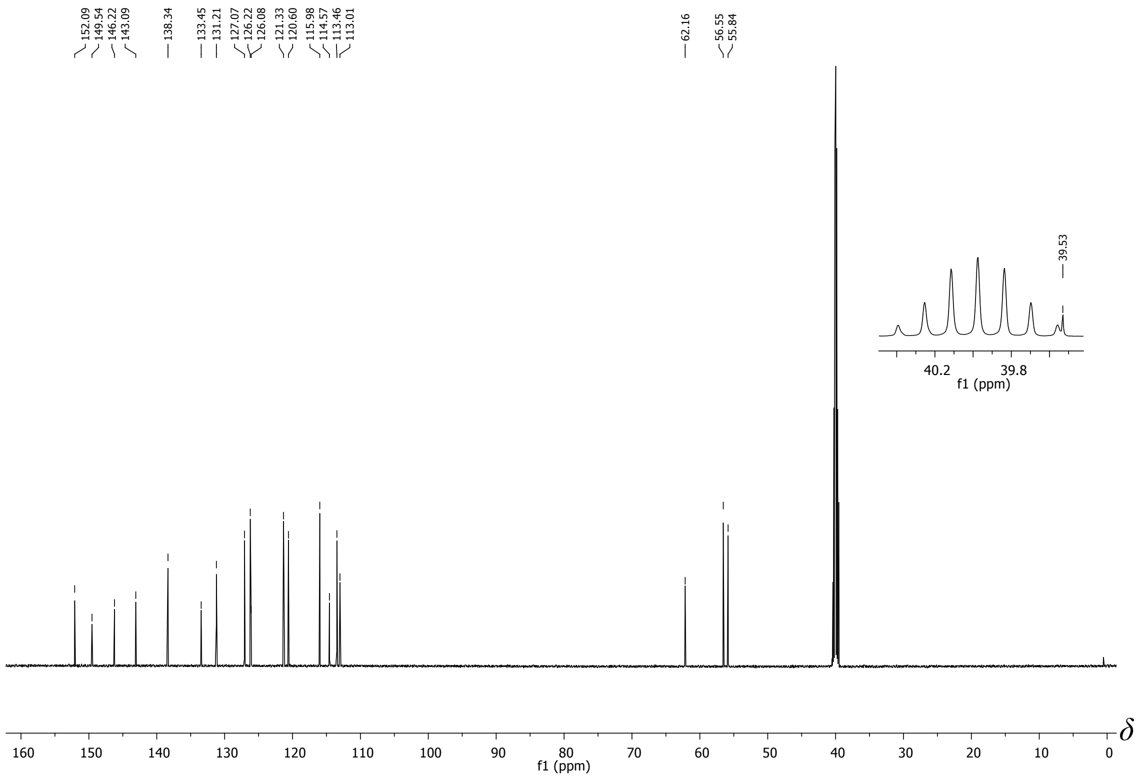

Figure S81. ^{13}C NMR spectrum (150 MHz, DMSO-d_6) of 4-((4-allyl-2-methoxyphenoxy)methyl)-1-(2-methoxyphenyl)-1*H*-1,2,3-triazole (**2p**).

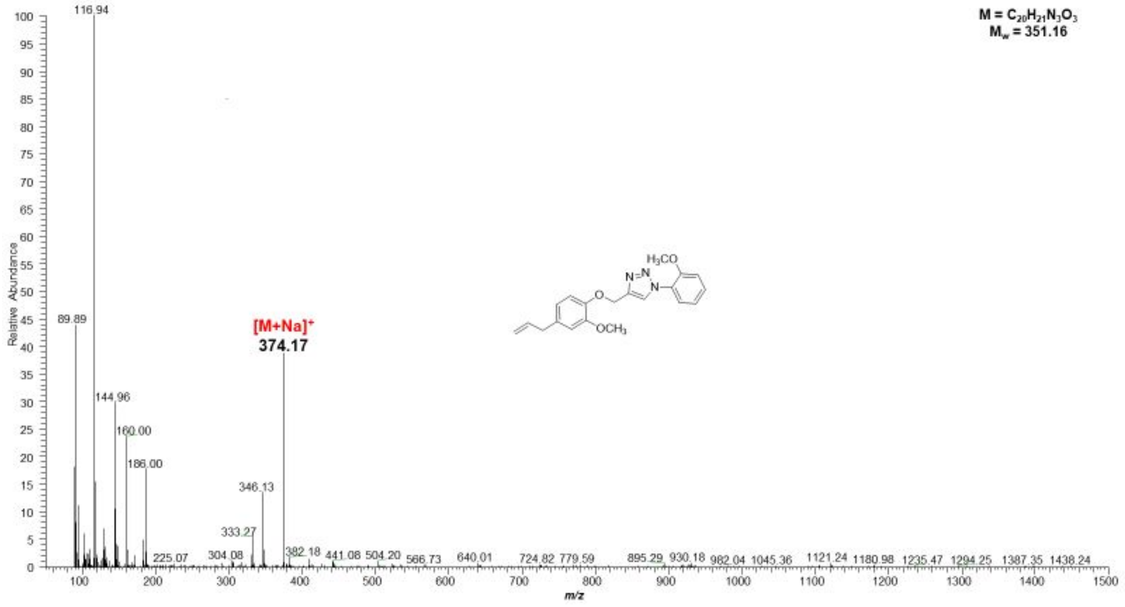

Figure S82. LC-MS spectrum of 4-((4-allyl-2-methoxyphenoxy)methyl)-1-(2-methoxyphenyl)-1*H*-1,2,3-triazole (**2p**).

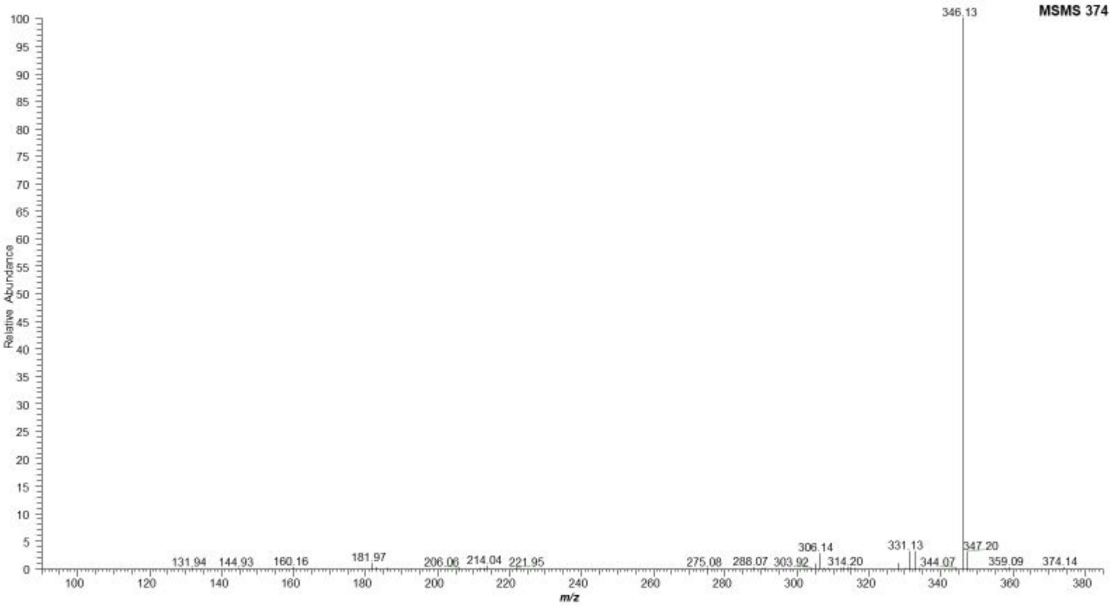

Figure S83. MS-MS spectrum of 4-((4-allyl-2-methoxyphenoxy)methyl)-1-(2-methoxyphenyl)-1*H*-1,2,3-triazole (**2p**).

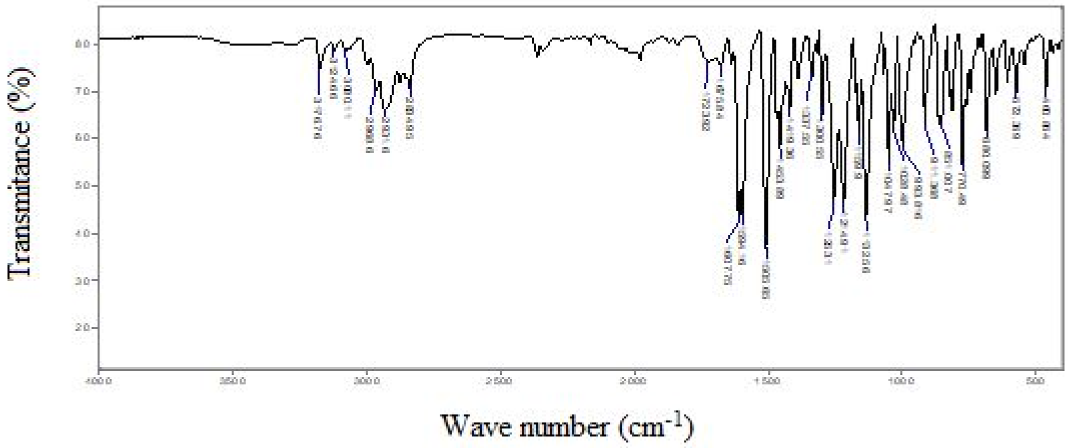

Figure S84. FTIR spectrum (ATR) of 4-((4-allyl-2-methoxyphenoxy)methyl)-1-(3-methoxyphenyl)-1*H*-1,2,3-triazole (**2q**).

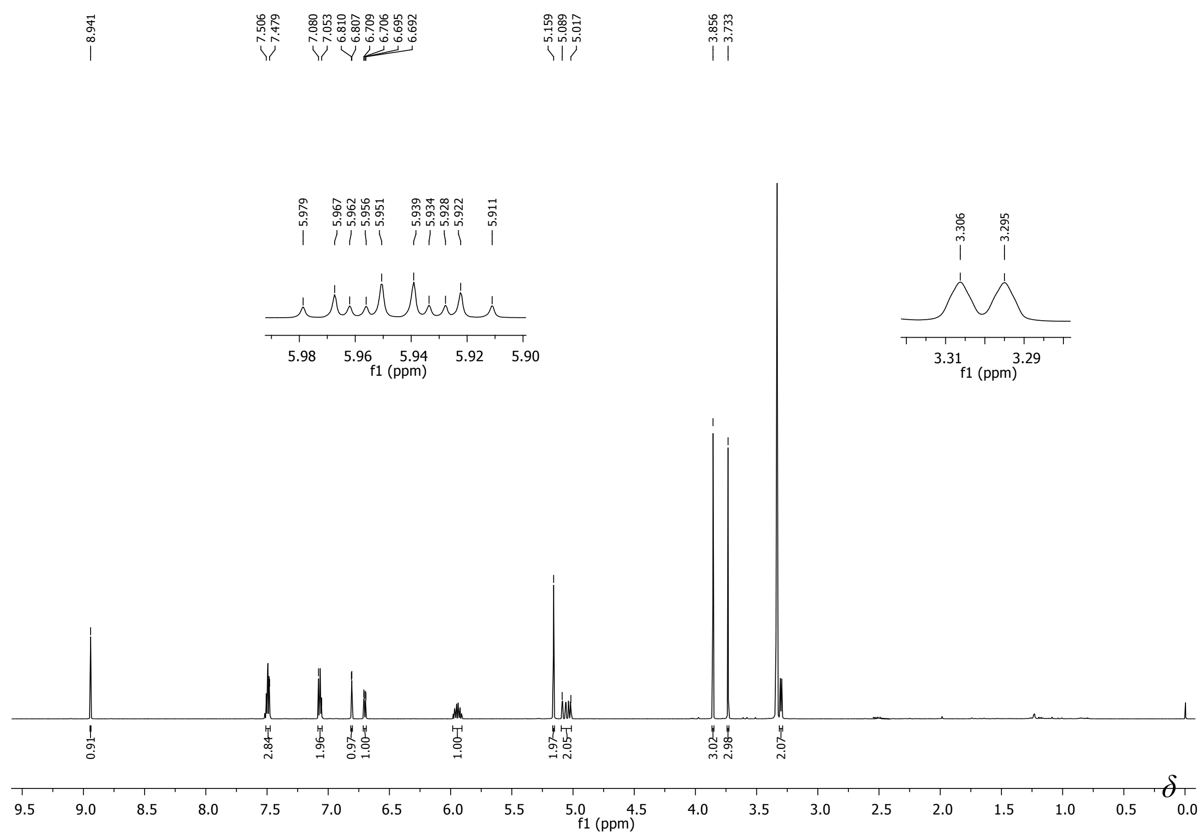

Figure S85. ^{1}H NMR spectrum (600 MHz, DMSO-d_6) of 4-((4-allyl-2-methoxyphenoxy)methyl)-1-(3-methoxyphenyl)-1*H*-1,2,3-triazole (**2q**).

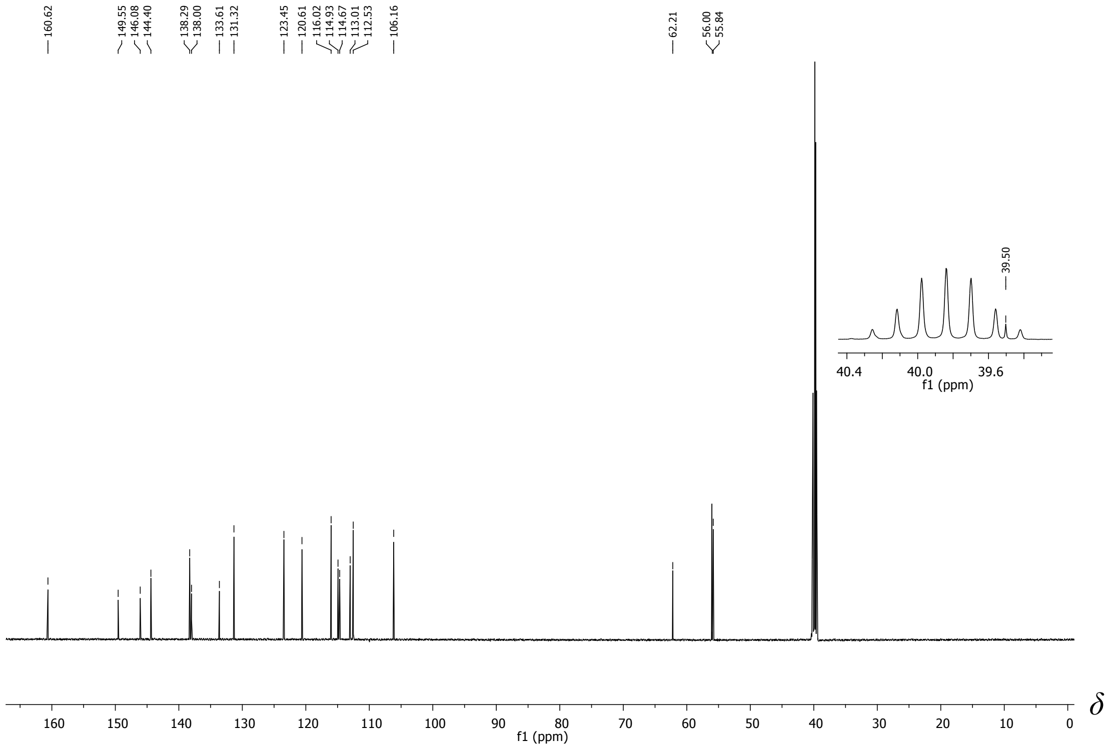

Figure S86. ^{13}C NMR spectrum (150 MHz, DMSO-d_6) of 4-((4-allyl-2-methoxyphenoxy)methyl)-1-(3-methoxyphenyl)-1*H*-1,2,3-triazole (**2q**).

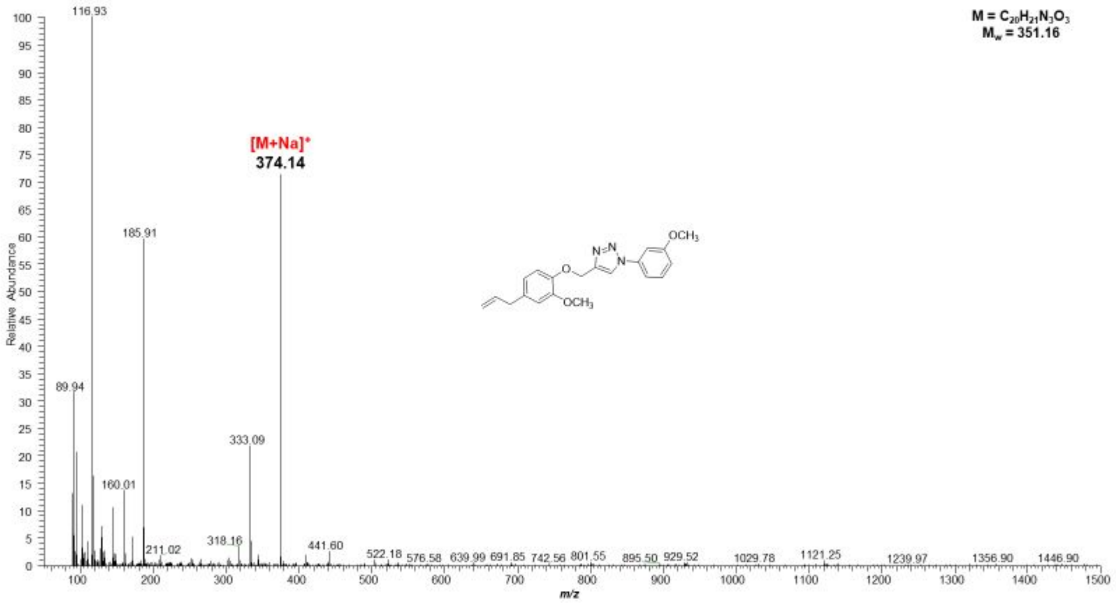

Figure S87. LC-MS spectrum of 4-((4-allyl-2-methoxyphenoxy)methyl)-1-(3-methoxyphenyl)-1*H*-1,2,3-triazole (**2q**).

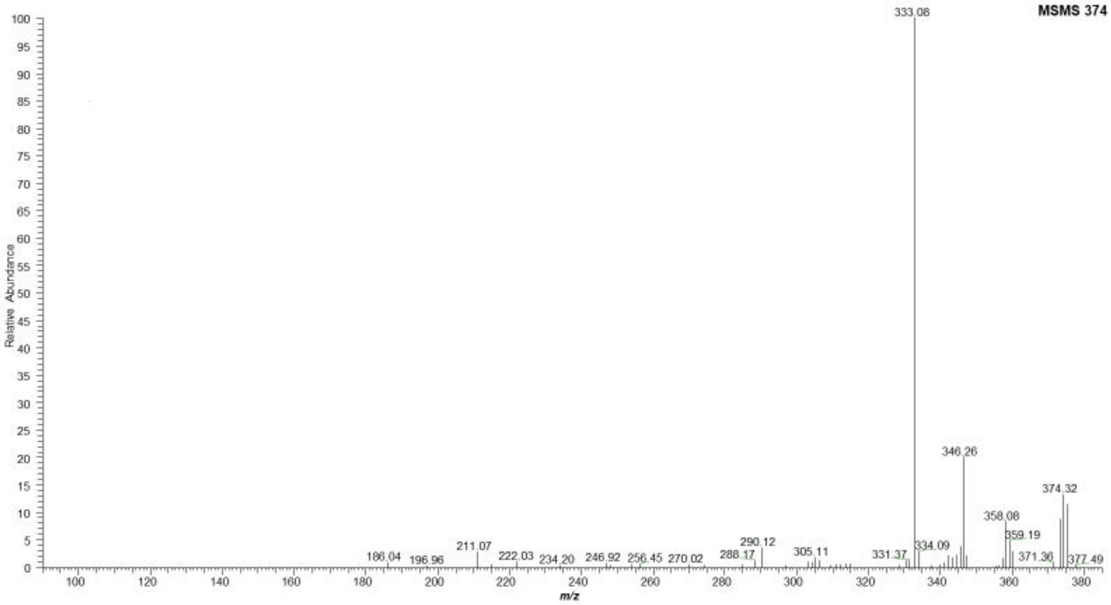

Figure S88. MS-MS spectrum of 4-((4-allyl-2-methoxyphenoxy)methyl)-1-(3-methoxyphenyl)-1*H*-1,2,3-triazole (**2q**).

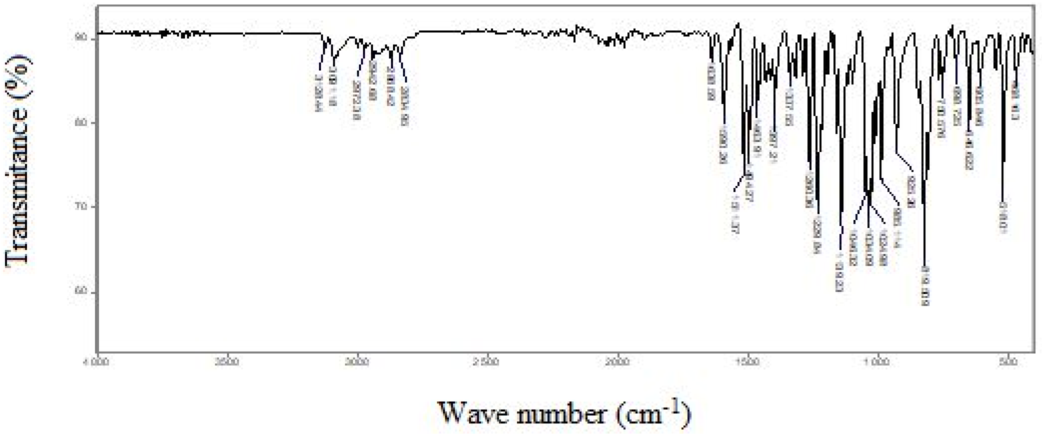

Figure S89. FTIR spectrum (ATR) of 4-((4-allyl-2-methoxyphenoxy)methyl)-1-(4-iodophenyl)-1*H*-1,2,3-triazole (**2r**).

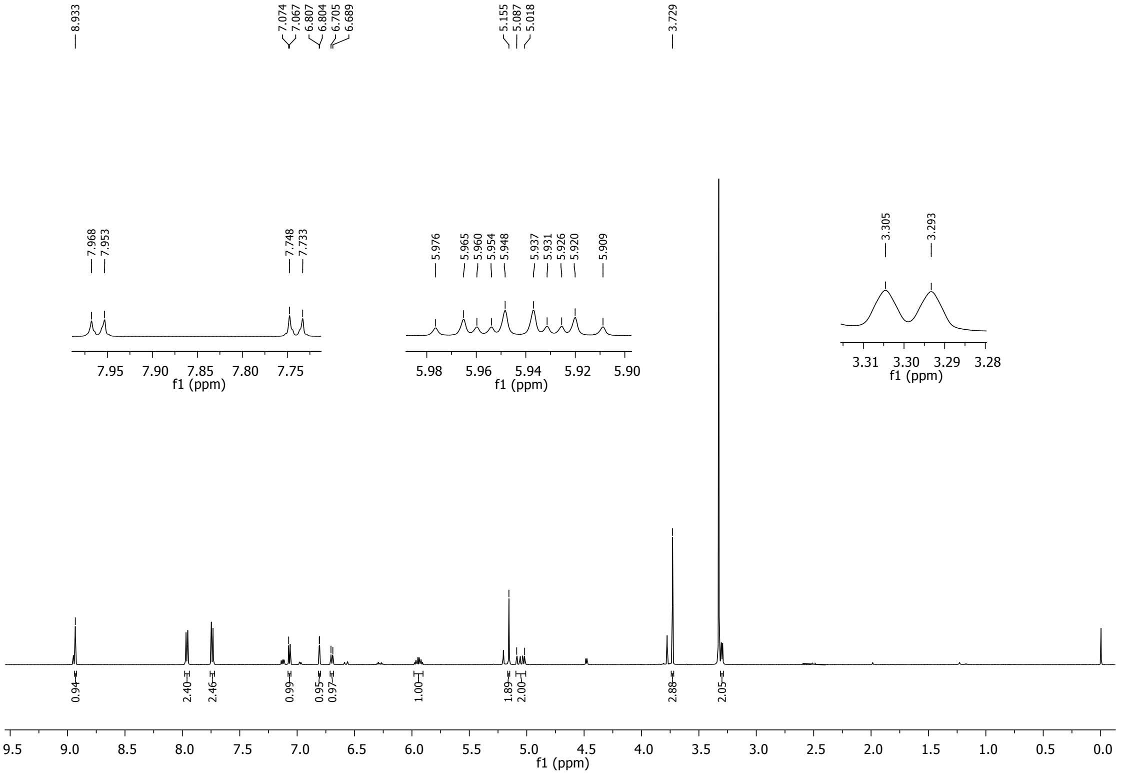

Figure S90. ^{1}H NMR spectrum (600 MHz, DMSO-d_6) of 4-((4-allyl-2-methoxyphenoxy)methyl)-1-(4-iodophenyl)-1*H*-1,2,3-triazole (**2r**).

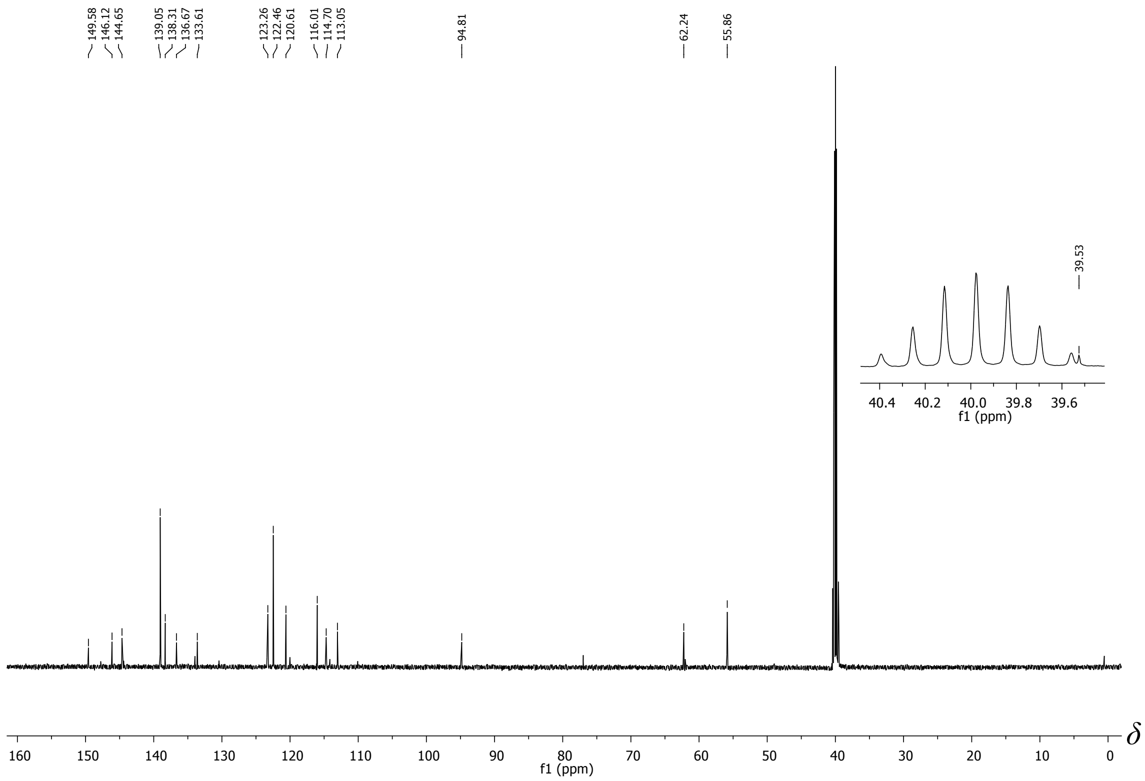

Figure S91. ^{13}C NMR spectrum (150 MHz, DMSO-d_6) of 4-((4-allyl-2-methoxyphenoxy)methyl)-1-(4-iodophenyl)-1*H*-1,2,3-triazole (**2r**).

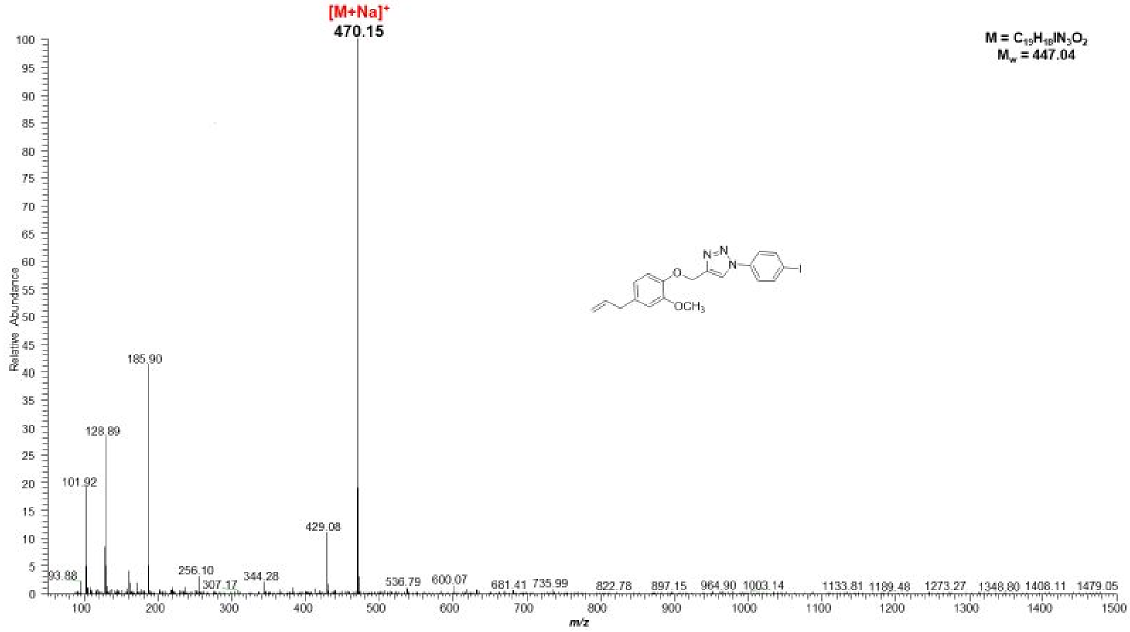

Figure S92. LC-MS spectrum of 4-((4-allyl-2-methoxyphenoxy)methyl)-1-(4-iodophenyl)-1*H*-1,2,3-triazole (**2r**).

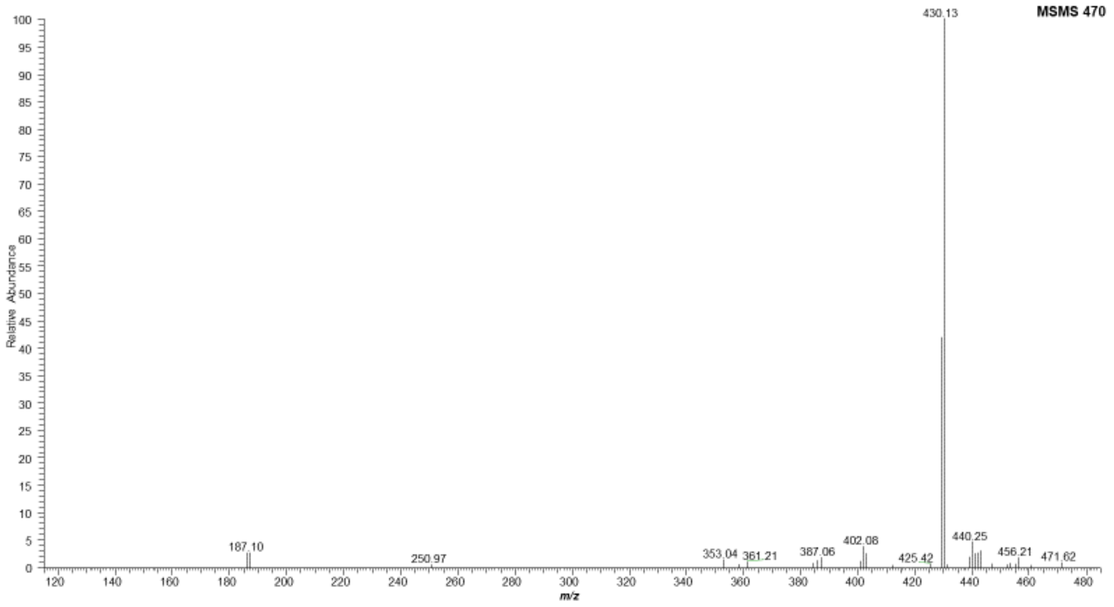

Figure S93. MS-MS spectrum of 4-((4-allyl-2-methoxyphenoxy)methyl)-1-(4-iodophenyl)-1*H*-1,2,3-triazole (**2r**).

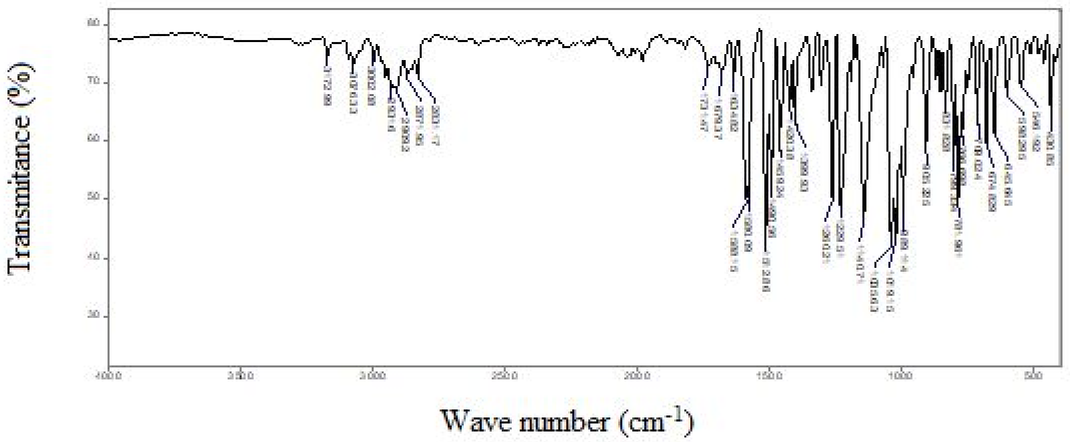

Figure S94. FTIR spectrum (ATR) of 4-((4-allyl-2-methoxyphenoxy)methyl)-1-(3-iodophenyl)-1*H*-1,2,3-triazole (**2s**).

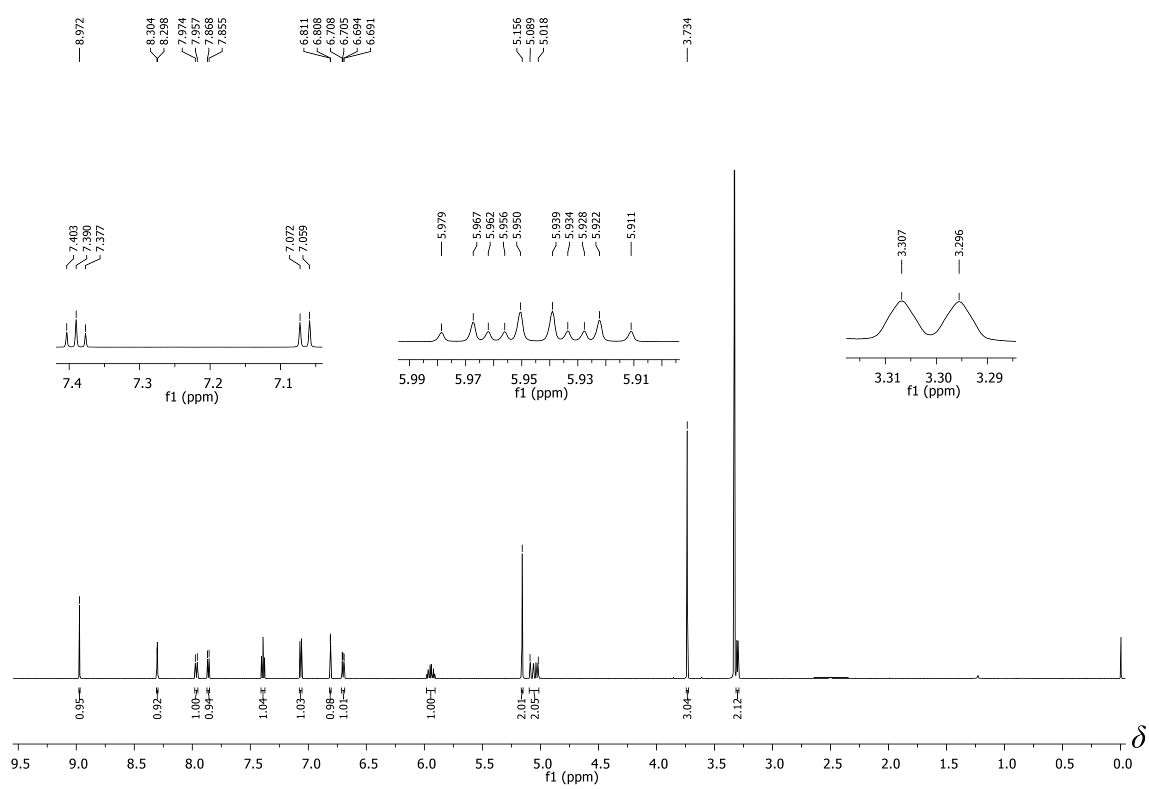

Figure S95. ^{1}H NMR spectrum (600 MHz, DMSO-d_6) of 4-((4-allyl-2-methoxyphenoxy)methyl)-1-(3-iodophenyl)-1*H*-1,2,3-triazole (**2s**).

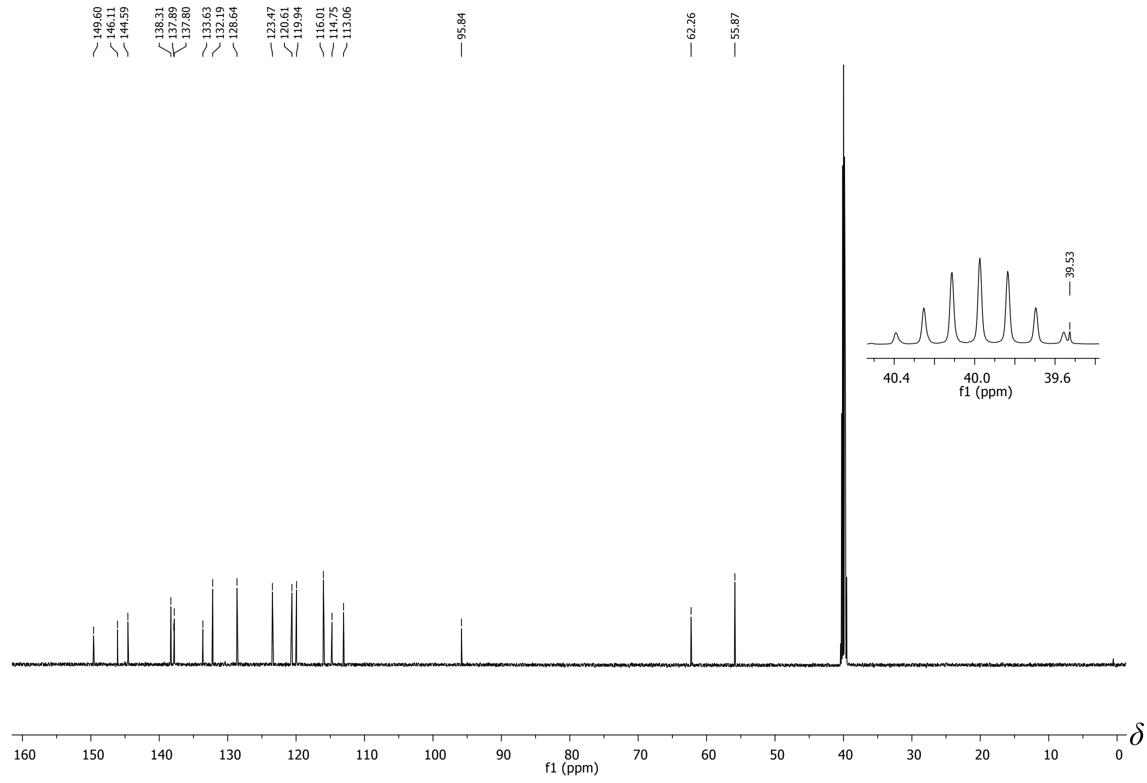

Figure S96. ^{13}C NMR spectrum (150 MHz, DMSO-d_6) of 4-((4-allyl-2-methoxyphenoxy)methyl)-1-(3-iodophenyl)-1*H*-1,2,3-triazole (**2s**).

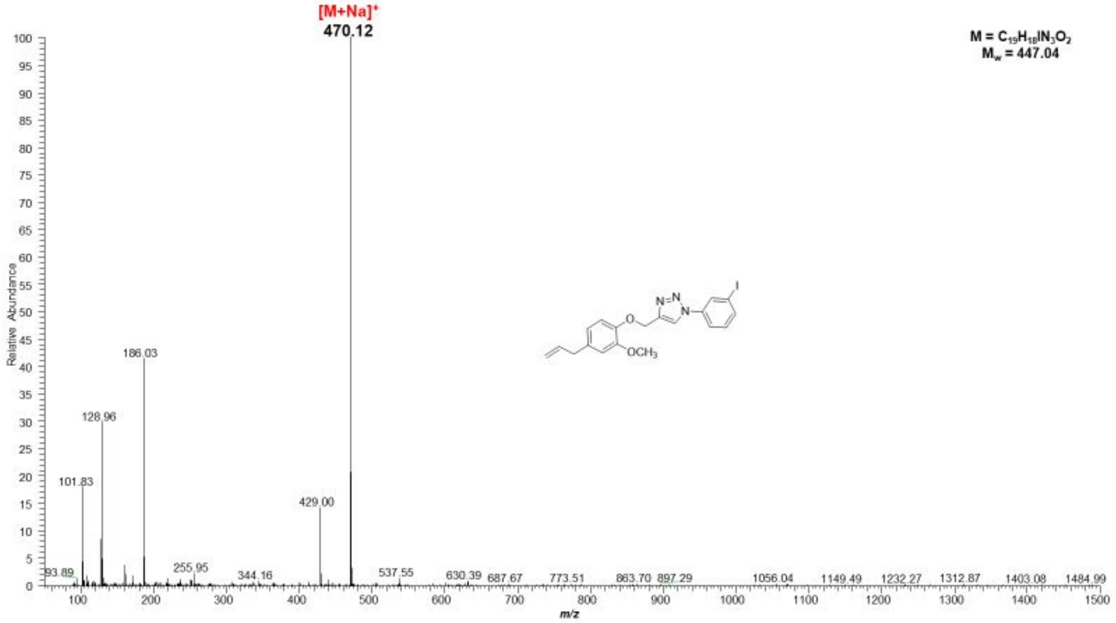

Figure S97. LC-MS spectrum of 4-((4-allyl-2-methoxyphenoxy)methyl)-1-(3-iodophenyl)-1*H*-1,2,3-triazole (**2s**).

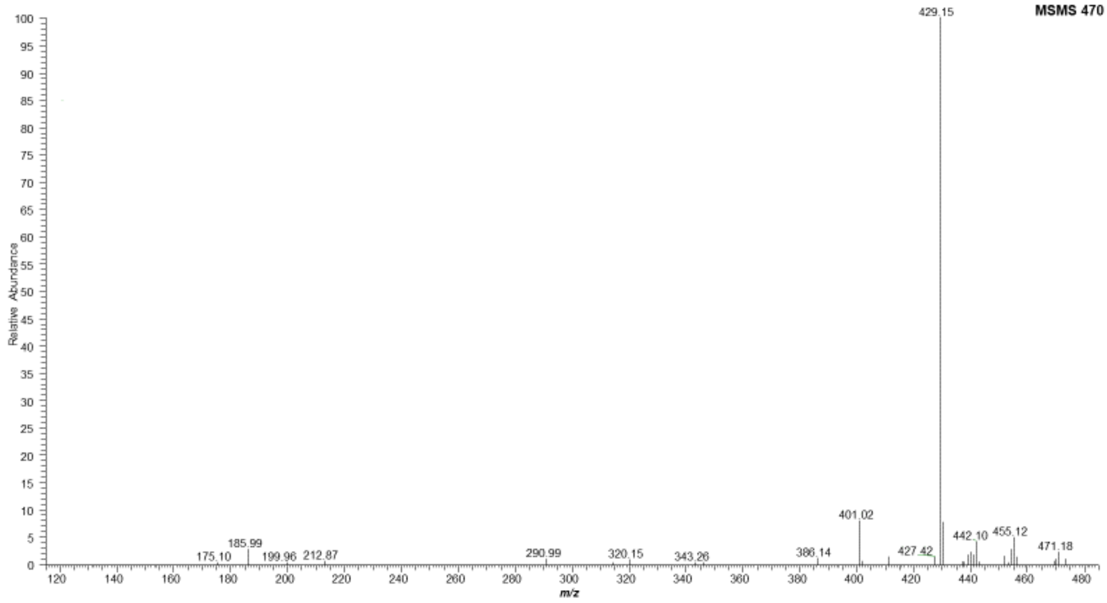

Figure S98. MS-MS spectrum of 4-((4-allyl-2-methoxyphenoxy)methyl)-1-(3-iodophenyl)-1*H*-1,2,3-triazole (**2s**).

3. COMPLEMENTARY MOLECULAR DOCKING DATA

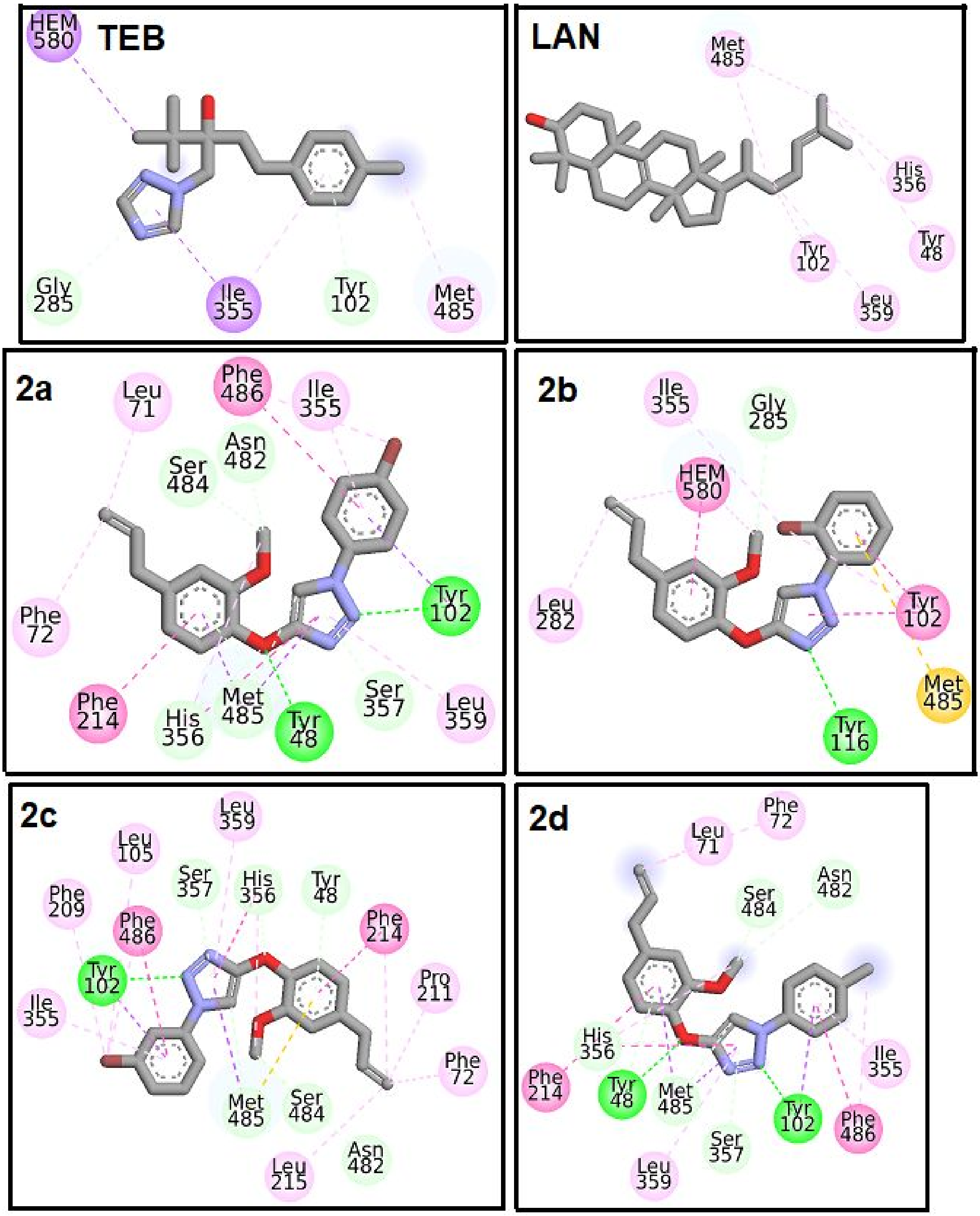

Figure S99. 2D ligand interaction diagram for all best-docked compounds into *C. gloeosporioides* CYP51 (CgCYP51A).

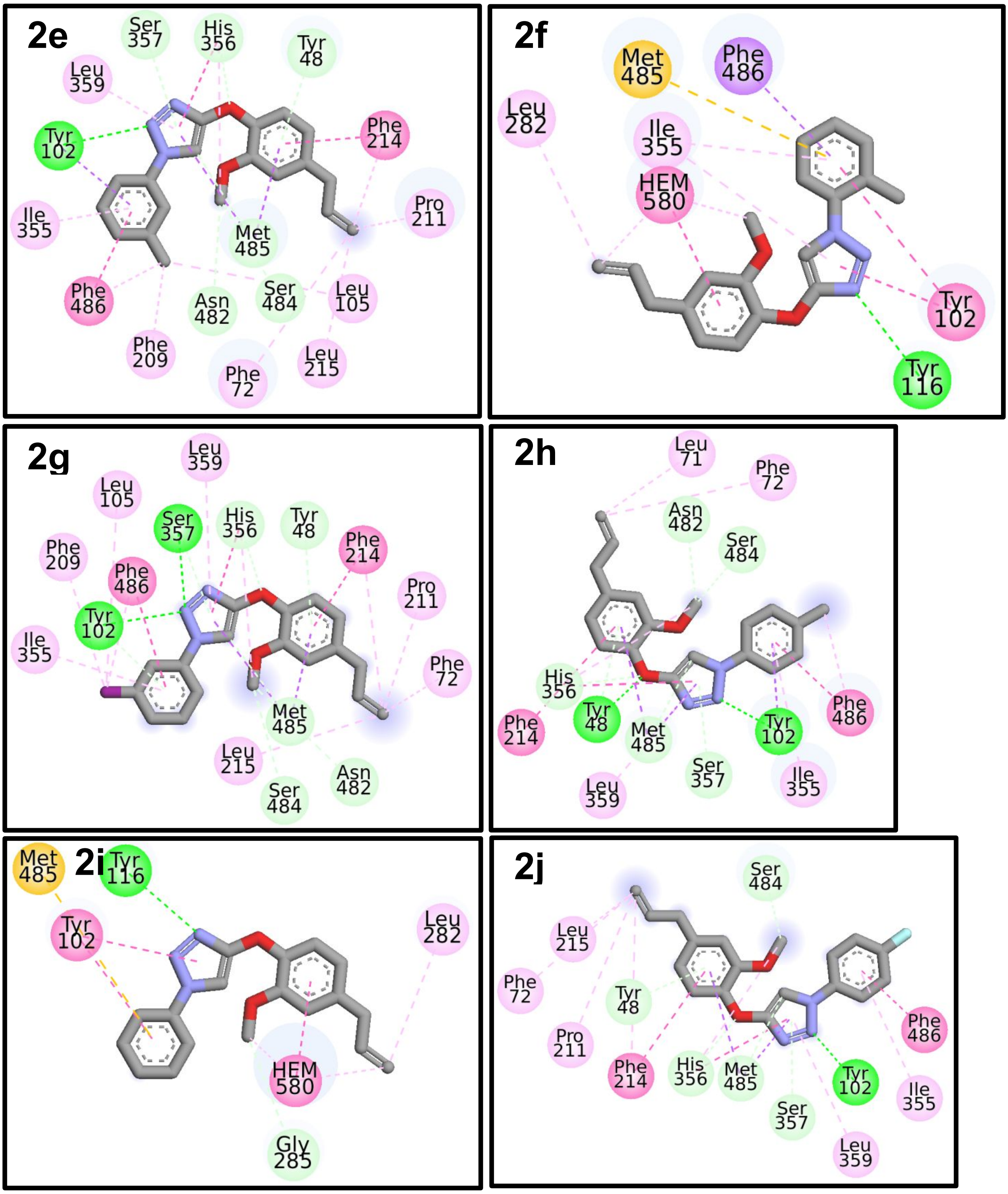

Figure S99. Continued.

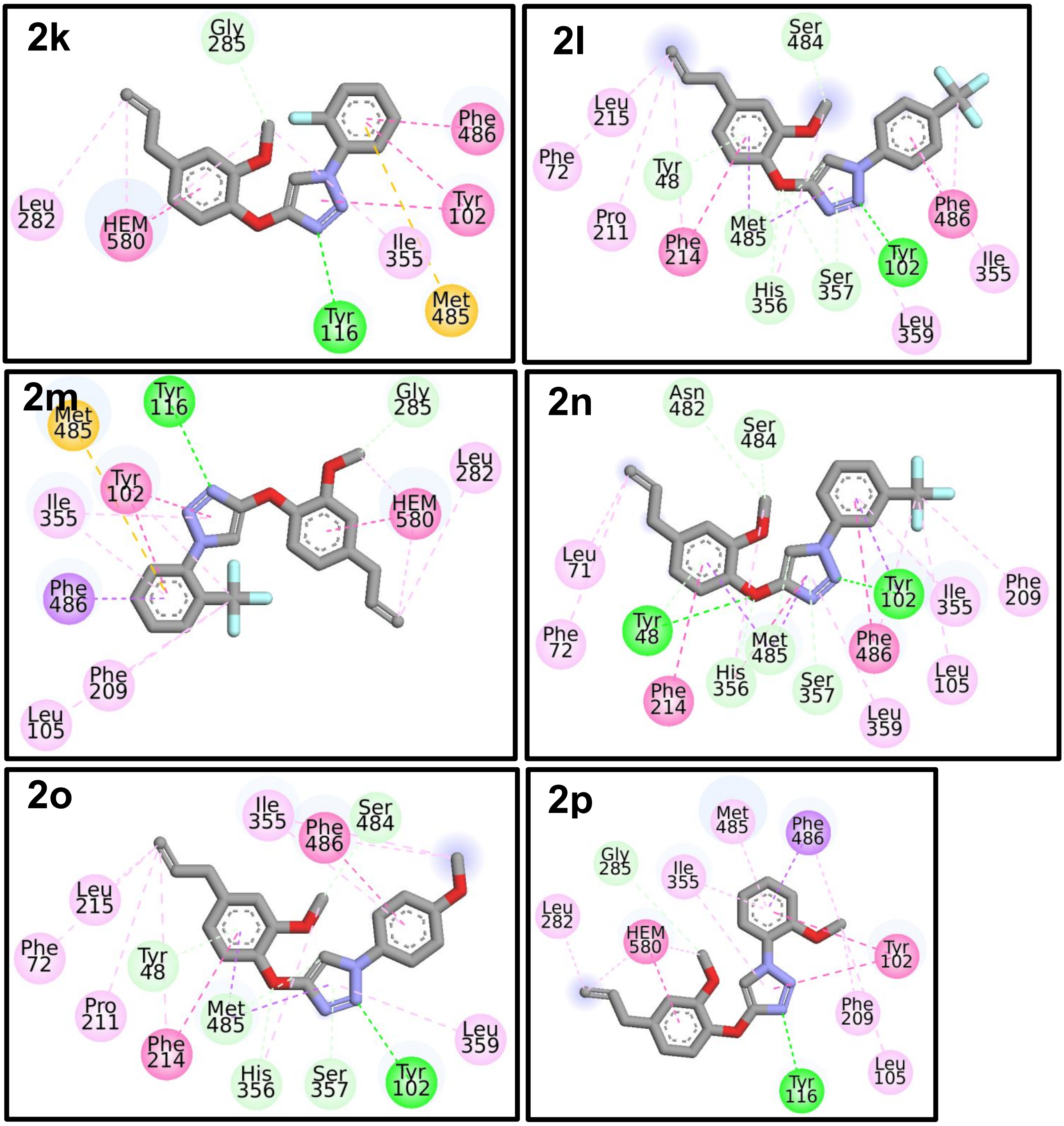

Figure S99. Continued.

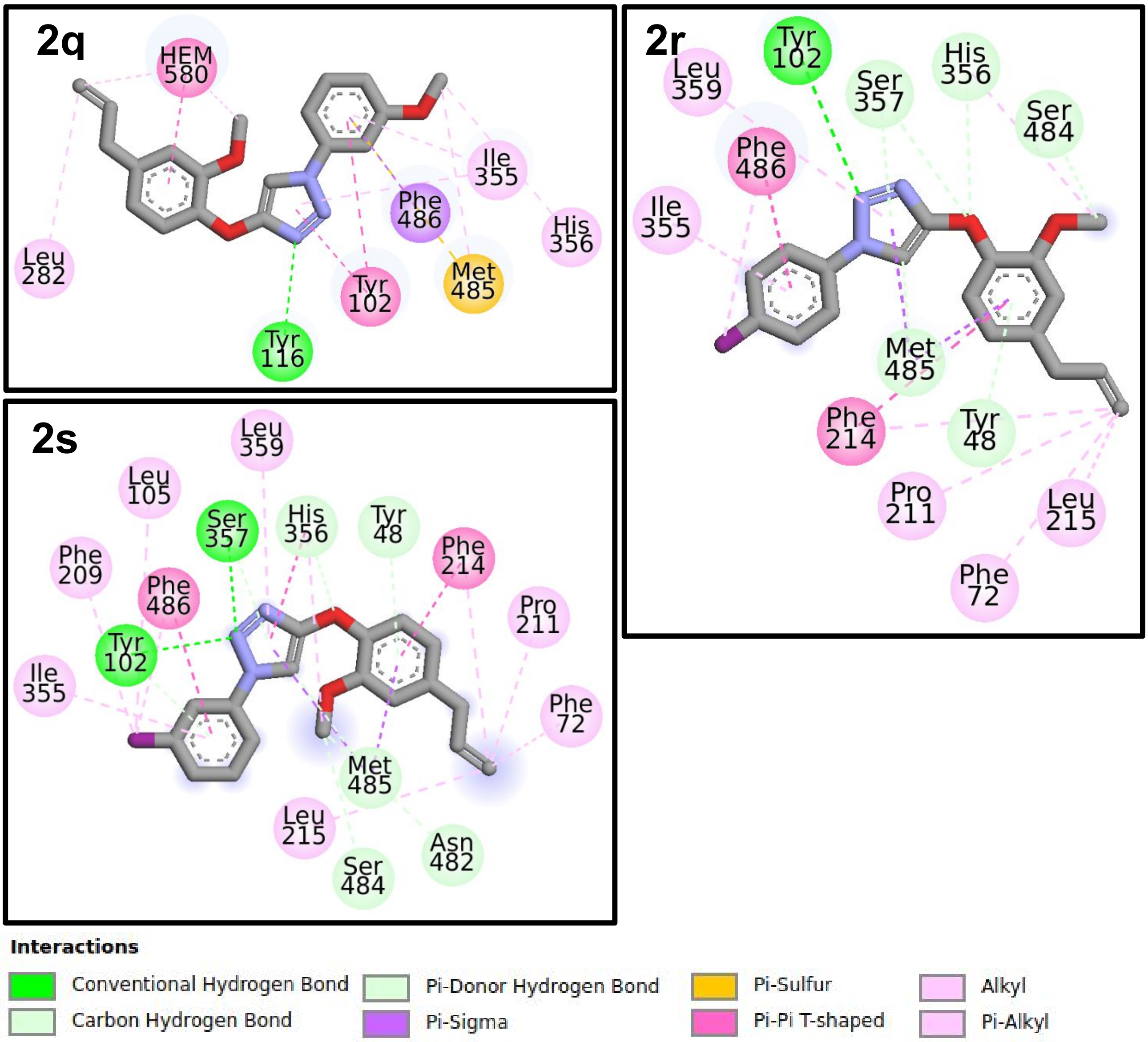

Figure S99. Continued.

Table S1. Binding energy (E_b, kcal mol^{-1}) obtained from molecular docking calculations and interacting residues with the triazole derivatives and CgCYP51A enzyme[a]

Compound	E_b	Residues	
		Non polar	Polar
**2a**	–9.1	Leu71, Phe72, Phe214, Ile355, Leu359, Phe486	Tyr48, Tyr102, His356, Ser357, Asn482, Ser484, Met485
**2b***	–9.3	Leu282, Gly285, Ile355,	Tyr102, Tyr116, Met485
**2c**	–9.4	Phe72, Leu105, Phe209, Pro211, Phe214, Leu215, Ile355, Leu359, Phe486	Tyr48, Tyr102, His356, Ser357, Asn482, Ser484, Met485
**2d**	–9.2	Leu71, Phe72, Phe214, Ile355, Leu359, Phe486	Tyr48, Tyr102, His356, Ser357, Asn482, Ser484, Met485
**2e**	–9.6	Phe72, Leu105, Phe209, Pro211, Phe214, Leu215, Ile355, Leu359, Phe486	Tyr48, Tyr102, His356, Ser357, Asn482, Ser484, Met485
**2f***	–9.6	Leu282, Ile355, Phe486	Tyr102, Tyr116, Met485
**2g**	–9.6	Phe72, Leu105, Phe209, Pro211, Phe214, Leu215, Ile355, Leu359, Phe486	Tyr48, Tyr102, His356, Ser357, Asn482, Ser484, Met485
**2h**	–9.2	Leu71, Phe72, Phe214, Ile355, Leu359, Phe486	Tyr48, Tyr102, His356, Ser357, Asn482, Ser484, Met485
**2i***	–9.1	Gly285, Leu282	Tyr102, Tyr116, Met485
**2j**	–9.3	Phe72, Pro211, Phe214, Leu215, Ile355, Leu359, Phe486	Tyr48, Tyr102, His356, Ser357, Ser484, Met485
**2k***	–9.2	Leu282, Gly285, Ile355, Phe486	Tyr102, Tyr116, Met485
**2l**	–9.8	Phe72, Pro211, Phe214, Leu215, Ile355, Leu359, Phe486	Tyr48, Tyr102, His356, Ser357, Ser484, Met485
**2m***	–10.1	Leu105, Phe209, Leu282, Gly285, Ile355, Phe486	Tyr102, Tyr116, Met485
**2n**	–10.1	Leu71, Phe72, Leu105, Phe209, Phe214, Ile355, Leu359, Phe486	Tyr48, Tyr102, His356, Ser357, Asn482, Ser484, Met485

Table S1. continued

**2o**	–9.2	Phe72, Pro211, Phe214, Leu215, Ile355, Leu359, Phe486	Tyr48, Tyr102, His356, Ser357, Ser484, Met485
**2p***	–9.2	Leu105, Phe209, Leu282, Gly285, Ile355, Phe486	Tyr102, Tyr116, Met485,
**2q***	–9.1	Leu286, Ile355, Phe486	Tyr102, Tyr116, His356, Met485
**2r**	–9.3	Phe72, Pro211, Phe214, Leu215, Ile355, Leu359, Phe486	Tyr48, Tyr102, His356, Ser357, Ser484, Met485
**2s**	–9.5	Phe72, Leu105, Phe209, Pro211, Phe214, Leu215, Ile355, Leu359, Phe486	Tyr48, Tyr102, His356, Ser357, Asn482, Ser484, Met485
Teb*	–8.0	Gly285, Ile355	Tyr102, Met485
LAN	–10.8	Leu359	Tyr48, Tyr102, His356, Met485

*G1 group; Teb = Tebuconazole; LAN = Lanosterol

4. MECHANISM OF THE CuAAC REACTION

The mechanism depicted below (**Figure S100**) is based on the investigation entitled 'Direct Evidence of a Dinuclear Copper Intermediate in Cu(I)-Catalyzed Azide-Alkyne Cycloadditions,' reported by B. T. Worrell, J. A. Malik, and V. V. Fokin in *Science* (2013, 340, 457-460). DOI: 10.1126/science.1229506. We illustrate the formation of compound **2a** from the reaction involving terminal alkyne **1** and 4-bromophenyl azide to clarify the steps involved in the mechanism. Initially, there is coordination between the terminal alkyne and the Cu(I) species resulting in the formation of intermediate (**i**), followed by the *in-situ* formation of the σ-bound copper acetylide (**ii**). Subsequently, a second π-bound copper atom is recruited, forming the catalytically active complex (**iii**). This is followed by the reversible coordination of the organic azide to the π-bound copper complex (structure **iv**). Finally, stepwise annulation events involving structures (**v**) and (**vi**) lead to the formation of 1,2,3-triazole-1,4-disubstituted products and regeneration of the catalytic species.

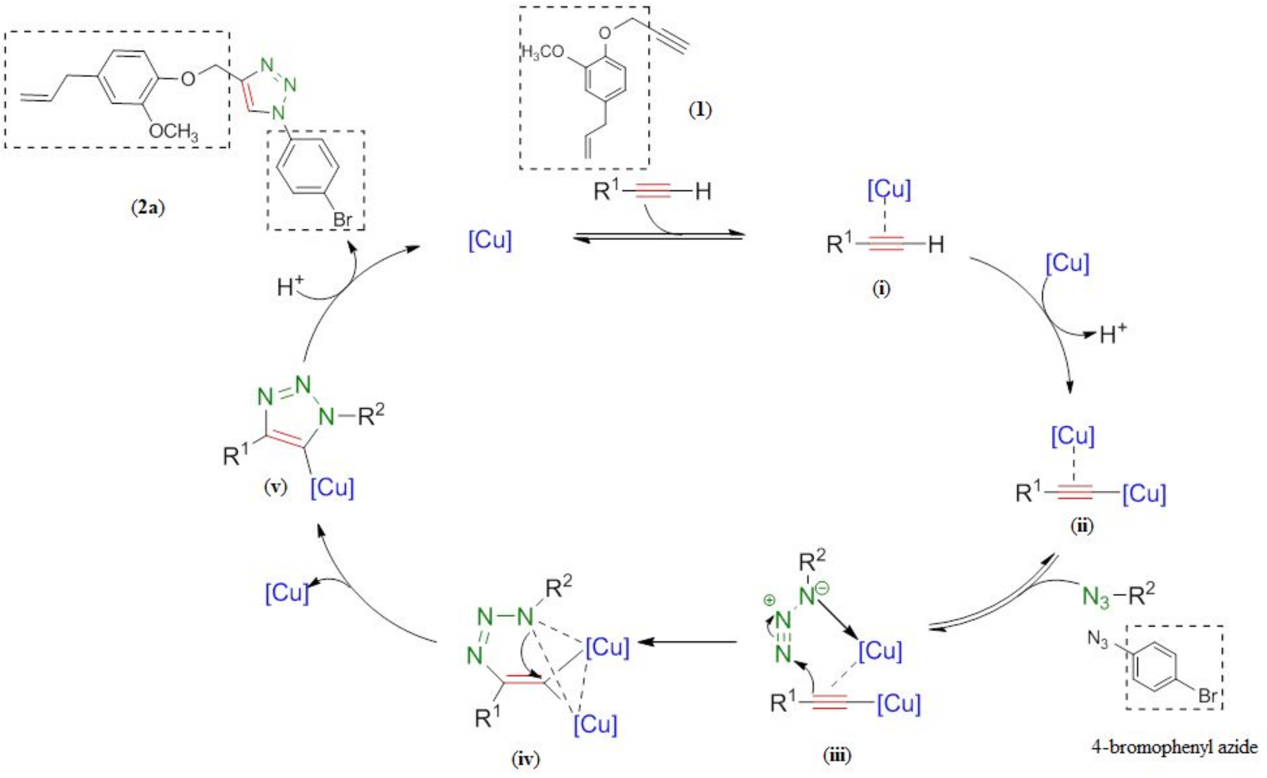

Figure S100. Mechanism for the CuAAC reaction highlighting the formation of compound **2a**.
